# Multiple invasions of *Gypsy *and *Micropia *retroelements in genus *Zaprionus *and *melanogaster *subgroup of the genus *Drosophila*

**DOI:** 10.1186/1471-2148-9-279

**Published:** 2009-12-02

**Authors:** Nathalia de Setta, Marie-Anne Van Sluys, Pierre Capy, Claudia MA Carareto

**Affiliations:** 1 UNESP - São Paulo State University, Department of Biology, São José do Rio Preto, SP, Brazil; 2 USP - São Paulo University, Department of Botany, São Paulo, SP, Brazil; 3 Laboratoire Evolution, Génomes et Spéciation UPR9034, CNRS, 91198 Gif-sur-Yvette, and Université Paris-Sud 11, 91405 Orsay, France

## Abstract

**Background:**

The *Zaprionus *genus shares evolutionary features with the *melanogaster *subgroup, such as space and time of origin. Although little information about the transposable element content in the *Zaprionus *genus had been accumulated, some of their elements appear to be more closely related with those of the *melanogaster *subgroup, indicating that these two groups of species were involved in horizontal transfer events during their evolution. Among these elements, the *Gypsy *and the *Micropia *retroelements were chosen for screening in seven species of the two *Zaprionus *subgenera, *Anaprionus *and *Zaprionus*.

**Results:**

Screening allowed the identification of diverse *Gypsy *and *Micropia *retroelements only in species of the *Zaprionus *subgenus, showing that they are transcriptionally active in the sampled species. The sequences of each retroelement were closely related to those of the *melanogaster *species subgroup, and the most parsimonious hypothesis would be that 15 horizontal transfer events shaped their evolution. The *Gypsy *retroelement of the *melanogaster *subgroup probably invaded the *Zaprionus *genomes about 11 MYA. In contrast, the *Micropia *retroelement may have been introduced into the *Zaprionus *subgenus and the *melanogaster *subgroup from an unknown donor more recently (~3 MYA).

**Conclusion:**

*Gypsy *and *Micropia *of *Zaprionus *and *melanogaster *species share similar evolutionary patterns. The sharing of evolutionary, ecological and ethological features probably allowed these species to pass through a permissive period of transposable element invasion, explaining the proposed waves of horizontal transfers.

## Background

**Figure 1:**
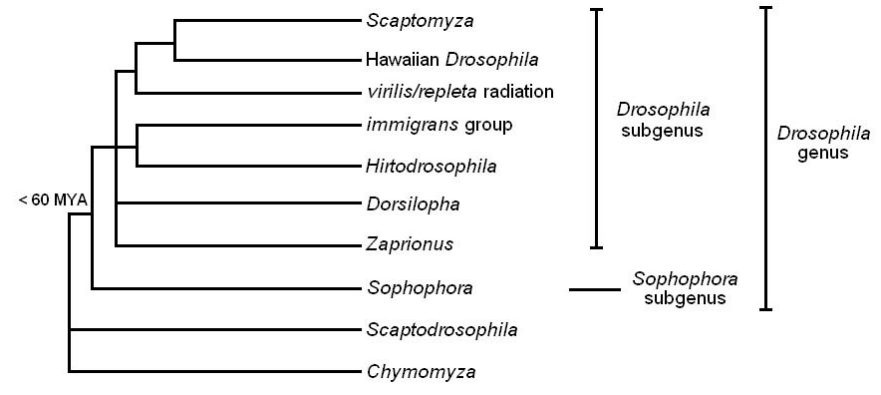
**Phylogenetic tree of the Drosophilidae family**. Phylogenetic relationships between the main Drosophilidae species groups, evidencing the taxonomic positioning of the *Zaprionus *genus inside the *Drosophila *subgenus and the divergence time between *Drosophila *and *Sophophora *subgenus [redrawn from reference [7]].

The *Zaprionus *genus (Diptera, Drosophilidae) is composed by two subgenera (*Anaprionus *s.s. and *Zaprionus *s.s.) which seem to be originated in Asia about 14 MYA [1]. The *Anaprionus *subgenus has first diversified in the Oriental biogeographic region, being relatively less studied. However, the *Zaprionus *subgenus ancestor(s) originated during the Middle to Early Miocene in Oriental regions before diversifying in Tropical Africa, sharing space and time with the best studied drosophilid species, the *melanogaster *subgroup [1,2]. Due to its evolutionary history, ecological and morphological diversity, the *Zaprionus *genus seems to be a good model for comparative studies with the *melanogaster *subgroup. Although the phylogenetic relationships within the *Zaprionus *genus had been recently proposed [1], its taxonomic positioning in the Drosophilidae family remains a matter for discussion. Currently, most reports agree that *Zaprionus *belongs to the *Drosophila *genus, and that it would be more closely related to *Drosophila *than to *Sophophora *subgenera [3-10], to which the species of the *melanogaster *subgroup belong (Figure 1). Hence, *Zaprionus *and *melanogaster *species would share a last common ancestor at least as old as the divergence of the *Sophophora *and *Drosophila *subgenera, i.e., between 35 and 60 MYA [5,11].

While the *melanogaster *species subgroup - mainly the two sibling species *D. melanogaster *and *D. simulans *- presents the most well known dipteran mobilomes, knowledge about the transposable elements (TEs) content of the *Zaprionus *species is scant. Only seven TE families have been identified in all *Zaprionus *genus. The elements *412 *of *Z. tuberculatus*, *731 *of *Z. ornatus*, *Bari-1 *of *Z. tuberculatus *and, *Mariner *of 12 *Zaprionus *species could only be detected by Southern blot hybridization using probes derived from *D. melanogaster *[12-17]. Furthermore, partial sequences were produced for *Gypsy *of *Z. indianus*, *Copia *of *Z. tuberculatus*, *Mariner *of *Z. tuberculatus *and *Z. verruca*, and *Rover *of *Z. indianus *[17-20]. Interestingly, evolutionary analyses demonstrated that four of these seven TEs of *Zaprionus *are highly related to those the *melanogaster *subgroup, sharing their common ancestor more recently than the species ones. *Gypsy*, *Copia*, *Mariner *and *Rover *were therefore proposed to have been horizontally transferred between species of the *Zaprionus *subgenus and the *melanogaster *subgroup of the genus *Drosophila *[15-18,20,21]. Horizontal transfer (HT) of TEs has been inferred when three criteria are met: (a) high sequence conservation between TE sequences of distantly related species; (b) incongruence between TE and host phylogenies and, (c) discontinuous distribution of a TE across a species group. Additionally, requirements like geographic, temporal and ecological overlapping and, exclusion of alternative hypothesis as ancestral polymorphism promoting differential subfamily fixation and high selective constraints over the sequences need to be considered [22].

In a previous study, we have performed a search of *D. melanogaster *TEs in the American continent invasive species *Z. indianus *by Dot blot methodology, aiming to broaden our understanding of TE occurrence in species of the *Zaprionus *genus [23]. All the 10 TEs analyzed were present in the *Z. indianus *genome, but only five had strong hybridization signals with the probes that were used: *Roo/B104*, *Doc*, *MDG-1*, *Micropia *and *Gypsy*. Among them, we selected *Gypsy *and *Micropia *retroelements to expand our evolutionary analyses in the *Zaprionus *genus. This choice was due to the evidence of the close relationship of these *Z. indianus *elements with others already described in the literature, inferred by the strong hybridization signals using heterologous probes, the easy amplification with the primers available in the literature and the proposition of a *Gypsy *HT of between *Z. indianus *and *D. simulans *[18].

*Gypsy *and *Micropia *are classified as *Ty3/Gypsy *elements because of their gene arrangement in the *pol *coding region - Reverse transcriptase/RNAseH/Integrase [24]. *Ty3/Gypsy *retroelements, which are similar to vertebrate retroviruses in both sequence and genomic structure, are broadly distributed among eukaryotes [25]. They differ from vertebrate retroviruses mainly because they do not have an *env *gene necessary to complete the infectious cycle [24]. *Gypsy *is an exception to the typical *Ty3*/*Gypsy *structure because an active *env *is present, potentially capable of maintaining infectivity [26]. Hence, *Gypsy *was classified as an *Errantivirus *by the International Committee on Taxonomy of Viruses (ICTV) [27].

The *Micropia *retroelement had already been studied in *D. melanogaster*, and several species of the *repleta *and the *cardini *groups of the subgenus *Drosophila *[28-30]. Evolutionary analyses showed the existence of at least two *Micropia *families that have ~30% divergence in DNA sequence [29]. The first family, present in the *repleta *group, comprises two subfamilies than were differentiated by nucleotide divergence and antisense RNA expression. The *repleta *species group seems to have transmitted one of its elements to the *D. cardini *group through HT [30]. The second family is represented by the *D. melanogaster *element that also transcribes antisense RNAs responsible for control of its expression [31]. The data suggest that the *Micropia *retroelement is an ancient component of *Drosophila *genomes [29].

In contrast to *Micropia*, the *Gypsy *retroelement has been analyzed in the 12 *Drosophila *genomes [32], and individual sequences from several Drosophilidae species are available, such as the *virilis *and *repleta *groups of subgenus *Drosophila*; the *willistoni *and *melanogaster *groups of the subgenus *Sophophora*; and the *Scaptodrosophila latifasciaeformis *and the *Z. indianus species *[18,32-34]. Two *Gypsy *families have been identified, one exclusive to *D. willistoni *and the other widely distributed among Drosophilidae species [18]. The latter is divided into 10 subfamilies, seven of which have been implicated in HT events, demonstrating that inheritance of the *Gypsy *retroelement is complex [18,32]. HT events involving *Gypsy *could have been facilitated by the presence of *env*, assumed to confer autonomous infectivity on the retroelement, thereby allowing it to cross species barriers [18,32,35]. Specifically in the *melanogaster *species subgroup, two *Gypsy *subfamilies have been identified, and seven HTs have been proposed among *melanogaster *species subgroups, or between one species of that subgroup and *S. latifasciaeformis *and *Z. indianus *[18,32]. These inter-genera HTs are assumed to have occurred because synonymous sites of *Gypsy env *gene are three to five times more highly conserved than those of host genes in species that diverged longer than 40 MYA [18].

We have now surveyed the *Gypsy *and *Micropia *retroelements in seven species of the *Zaprionus *genus, comprising one species of the *Anaprionus *subgenus and six of the *Zaprionus *subgenus. Our objective was to investigate the distribution and evolutionary history of these two elements in this scarcely studied genus by comparing their sequences with those of the 12 *Drosophila *genomes and other sequences available in the nucleotide database. The results show that the *Zaprionus Gypsy *and *Micropia *retroelements are present and transcriptionally active only in the subgenus *Zaprionus*, to which they were introduced by more than one horizontal transfer event from different donor species.

## Results

### Distribution of *Gypsy *and *Micropia *in the genus *Zaprionus*

In an attempt to search for the presence of *Gypsy *and *Micropia *retroelements in seven species of the *Zaprionus *genus (Table 1), PCR reactions using primers that amplify *Gypsy env *and *Micropia *RNAseH sequences were carried out (see Materials and Methods). This revealed the presence of these elements in the six species of the *Zaprionus *subgenus, but not in *Z. multistriatus *(*Anaprionus *subgenus). Three clones of each *Gypsy *and *Micropia *PCR fragments were sequenced and used in the evolutionary analyses. Additionally, RT-PCR reactions and Southern blot hybridization showed that all *Zaprionus *subgenus species analyzed harbor a few transcriptionally active insertions of *Gypsy *and *Micropia *elements, varying from one (*Z. gabonicus*) to seven (*Z. indianus *and *Z. davidi*) insertions for *Gypsy*, and from two (*Z. tuberculatus *and *Z. africanus*) to seven (*Z. indianus*) for *Micropia *[Additional files 1 and 2].

**Table 1:** *Zaprionus *species used in this study, taxonomic classification, divergence time, geographic origin of the strains and GenBank sequence accession numbers.

			GenBank accessions	
			
Species	**Divergence time **[1] (MYA)	Strain origin	*Gypsy*	*Micropia*
Genus *Zaprionus*	13.8 (10.9-14.9)			
Subgenus *Anaprionus*	10.6 (7.7-12.1)			
*Z. multistriatus*	nd	Bangalore (India)	Absent	Absent
Subgenus *Zaprionus*	7.4 (6.7-9.0)			
Group *inermis*	7.0			
*tuberculatus *complex	3.0 (2.9-18.0)			
*Z. tuberculatus*	1.1 (0.6-1.6)	Ithala (South Africa)	FJ710406 to FJ710408	FJ710423 to FJ710425
Group *armatus*	nd			
*lachaisei *complex	0.9 (0.4-1.1)			
*Z. camerounensis*	nd	Amani (Tanzania)	FJ710409 to FJ710411	FJ710426 to FJ710428
*davidi *complex	2.2 (1.4-2.4)			
*Z. davidi*	2.2 (1.4-2.4)	São Tomé (São Tomé e Príncipe)	FJ710412 to FJ710414	FJ710429 to FJ710431
*indianus *complex	3.1 (2.6-4.1)			
*Z. gabonicus*	nd	Makokou (Gabon)	FJ710415 to FJ710417	FJ710432 to FJ710434
*Z. africanus*	nd	Kibale (Uganda)	FJ710418 to FJ710420	FJ710435 to FJ710437
*Z. indianus*	nd	Brasília (Brazil)	FJ710421 to FJ710422	FJ710438 to FJ710440

nd: not determined.

### Evolutionary analyses of the *Gypsy *retroelement

To infer the evolutionary history of *Gypsy *retroelement in the *Zaprionus *subgenus, we carried out genomic searches for *Zaprionus *homologous sequences in the 12 *Drosophila *genomes and the GenBank database. The *in silico *search retrieved genomic sequences only from the *melanogaster *subgroup, indicating that the other seven *Drosophila *genomes (*D. ananassae*, *D. pseudoobscura*, *D. persimilis*, *D. willistoni*, *D. mojavensis*, *D. virilis *and *D. grimshawi*) do not harbor *Gypsy *sequences closely related to those of the subgenus *Zaprionus*. Eight sequences were identified in *D. melanogaster*, nine in *D. simulans*, 19 in *D. sechellia*, six in *D. yakuba*, and 13 in *D. erecta *[Additional file 3]. A BLAST-based analysis of the structure sequence indicated that only *D. melanogaster *genome presents putatively full-length insertions [Additional file 4].

#### Inferring phylogenetic relationships and divergence

**Figure 2:**
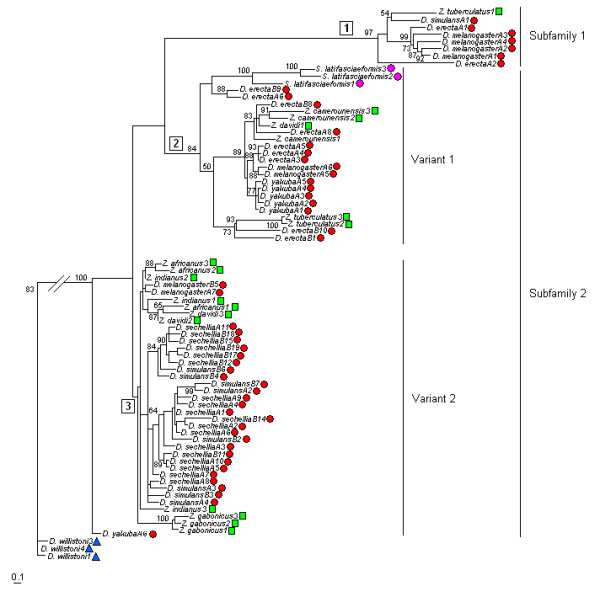
**Phylogenetic relationships between *Gypsy *retroelements**. Phylogeny of *Gypsy *sequences of genus *Zaprionus *(green squares), the *melanogaster *subgroup (red circles), *D. willistoni *(blue triangles) and *S. latifasciaeformis *(pink circles). Numbers in squares indicate the clade number mentioned in the text. The tree was reconstructed using the maximum-likelihood method (HKY85 distance), as implemented in the PhyML program. The branch support was calculated using bootstrap test (1000 replications). Numbers next to species names indicate the clone or genomic sequence identification.

The phylogenetic relationships inferred using the Maximum Parsimony (MP), Maximum Likelihood (ML) and Neighbor-joining (NJ) methods of phylogenetic reconstruction produced similar patterns with identical main branches. The analyses were performed with three sequences of each *Zaprionus *species and 52 sequences of species from the *melanogaster *subgroup. The *S. latifasciaeformis *and *D. willistoni Gypsy *sequences available in the GenBank database were included in these reconstructions because of their respectively closer and more distant evolutionary relationships to those of the *melanogaster *subgroup [18]. Figure 2 shows the tree reconstructed by ML analysis. The sequences of the genus *Zaprionus*, the *melanogaster *species subgroup and *S. latifasciaeformis *were grouped together in three clades that were not congruent with the species phylogeny. Clade 1 (bootstrap 97%) contains sequences of *D. melanogaster*, *D. erecta*, *D. simulans *and only one sequence of the genus *Zaprionus*, *Z. tuberculatus*1. Clade 2 (bootstrap 84%) includes *Z. tuberculatus*, *Z. camerounensis*, *S. latifasciaeformis*, *D. melanogaster*, *D. erecta *and all sequences of *D. yakuba*, except *D. yakubaA6*, which branches outside the 3 clades. The reported clustering of *D. erecta *and *S. latifasciaeformis *has been explained as an HT event [31]. Finally, clade 3 comprises the *Gypsy *sequences of *Z. davidi*, *Z. indianus *and the two cryptic species, *Z. gabonicus *and *Z. africanus*, as well as sequences of *D. melanogaster*, *D. simulans *and all sequences of *D. sechellia*, with no bootstrap support. Additionally, a tree was reconstructed using the *Zaprionus *sequences, the genomic sequences of *melanogaster *subgroup, and *Gypsy *sequences of 17 other *Drosophila *species (from the *guarani*, *cardini*, *pallidipennis*, *tripunctata*, *repleta*, *anulimana *and *flavopilosa *groups of the subgenus *Drosophila *and *D. busckii *[18]). A similar topology of the Figure 2 was obtained, with all *Zaprionus *sequences clustering together with *melanogaster *elements in the same clade distribution, which reinforces the close relationship between *Zaprionus *and *melanogaster Gypsy *sequences (data not shown).

Distance analysis shows that the sequences within the three clades have relatively low divergence levels, with mean values of 0.063, 0.070 and 0.042 within clades 1, 2 and 3, respectively (Table 2 and Additional file 5). The distances of the *Zaprionus *vs *melanogaster *species within each group are similar to the mean values just cited. The mean distances between the clades show that clade 2 is closer to clade 3 than to clade 1, with mean values of 0.119 (clade 2 vs clade 3), 0.239 (clade 1 vs clade 2) and 0.241 (clade 1 vs clade 3). The *Drosophila Gypsy *sequences, which vary up to 20% in nucleotide composition, were previously classified into 10 subfamilies [18,32]. Among them, the sequences of *Z. indianus *and the *melanogaster *species subgroup were included in two different subfamilies. The addition of 18 sequences belonging to six *Zaprionus *species allowed us to confirm this proposition. The species distribution and divergence of the clade 1 from 2 and 3 (0.24) indicate that it corresponds to one of those subfamilies previously proposed, hereafter called subfamily 1 (S1). On the other hand, a divergence of 0.119 between clades 2 and 3 led us to classify them as variants 1 and 2 (V1 and V2) of another subfamily (S2) that may correspond with the second subfamily already described due to its species composition.

**Table 2:** Genetic divergence between the *Gypsy *and *Micropia *retroelements

Retroelement	N	Mean	Minimum	Maximum
*Gypsy*				
clade 1 - S1	28	0.0628	0.0213	0.1112
*Zaprionus *vs *melanogaster *S1	7	0.0742	0.0529	0.1006
clade 2 - S2V1	300	0.0704	0.0021	0.1687
*Zaprionus *vs *melanogaster *V1	96	0.0641	0.0211	0.1122
clade 3 - S2V2	741	0.0422	0.0042	0.0888
*Zaprionus *vs *melanogaster *V2	308	0.0506	0.0086	0.0888
clade 1 (S1) vs clade 2 (V1)	200	0.2388	0.2080	0.2910
clade 1 (S1) vs clade 3 (V2)	312	0.2406	0.2055	0.286
clade 2 (V1) vs clade 3 (V2)	975	0.1189	0.0771	0.1814
*Micropia*				
clade 1	66	0.0091	0	0.0216
clade 2	496	0.0509	0	0.1473
clade 1 vs clade 2	384	0.0739	0.0288	0.1341
*Zaprionus *× *melanogaster *group	468	0.0727	0.0188	0.1341
*Zaprionus *genus	153	0.0328	0	0.0850
*melanogaster *group	325	0.0466	0	0.1473

Note: The MCL pairwise distance values between the *Zaprionus *subgenus and *melanogaster *species subgroup were summarized according the *Gypsy *subfamilies and variants and, species groups for *Micropia*. S1 - Subfamily 1; S2 - Subfamily 2; V1 - Variant 1; V2 - Variant 2. N: number of pairwise comparisons.

#### Testing the HT hypothesis

Three hypotheses could explain the high levels of conservation among *Gypsy *sequences within each clade (0.04-0.07) and the phylogenetic inconsistencies compared to the species tree: (a) high selective constraints conserving the sequences; (b) ancestral polymorphism promoting differential subfamily fixation in the species; and (c) the occurrence of HT. To test whether (a) can best explain the estimated conservation, the ratio of non-synonymous divergence per synonymous divergence (dN:dS) within the clades were compared to those of the *Gpdh *nuclear gene, offering a comparative measurement of the selective constraints. Although active elements might suffer selective pressure to maintain their coding sequences, *Gpdh *would be expected to be even more restricted because it plays an essential role in glycerophospholipid metabolism in *Drosophila*. If it is assumed that synonymous substitutions are under almost strictly neutral evolution, dN:dS = 1, dN:dS <1 and dN:dS >1 will represent neutral evolution, purifying selection and positive Darwinian selection, respectively. The analyses indicate that the mean *Gypsy *dN:dS ratios ranged from 0.153 to 1.002, while the mean *Gpdh *values did not exceed 0.041 (Table 3, and Additional file 6). These ratios suggest that, although dN:dS <1, purifying selection over the *Gypsy env *sequences is relaxed (dN:dS mean = 0.395) compared to the host gene. Also, a Z-test for the *Gypsy env *region indicates neutrality for most of the pairwise comparisons (62%) within the clades [Additional file 7]. Hence, high selective constraints do not explain the incongruities observed intra-clades. On the other hand, the neutrality hypothesis was refuted for all pairwise comparisons between the clades, indicating that purifying selection could plays a role in the conservation of the *env *coding sequence among the *Gypsy *subfamilies. In that case, natural selection could act at the genomic level, conserving the TE genes, since elements that transpose most efficiently, or at the highest rate are most likely to survive and propagate [36].

**Table 3:** Comparative analyses of synonymous divergence (dS), non-synonymous divergence (dN), dN:dS ratios and codon bias index (CBI) of the *Gypsy *and *Micropia *sequences and *Gpdh*.

Sequences	CBI mean (min-max)	dN mean (SE)	dS mean (SE)	dN:dS mean (SE)
*Gypsy*	0.414 (0.344-0.523)			
Subfamily 1				
*Zaprionus *× *melanogaster *spp.		0.045 (9.70 × 10^-8^)	0.128 (5.95 × 10^-4^)	0.374 (0.004)
*melanogaster *spp.		0.035 (3.88 × 10^-5^)	0.057 (6.49 × 10^-4^)	1.002 (0.465)
Subfamily 2 - V1				
*Zaprionus *× *melanogaster *spp.		0.028 (3.55 × 10^-5^)	0.176 (0.002)	0.189 (0.005)
*Zaprionus *spp.		0.032 (4.42 × 10^-5^)	0.177 (0.004)	0.310 (0.019)
*melanogaster *spp.		0.019 (4.85 × 10^-5^)	0.119 (0.002)	0.153 (0.002)
Subfamily 2 - V2				
*Zaprionus *× *melanogaster *spp.		0.028 (2.65 × 10^-5^)	0.106 (2.62 × 10^-4^)	0.291 (0.004)
*Zaprionus *spp.		0.027 (2.50 × 10^-5^)	0.088 (2.55 × 10^-4^)	0.365 (0.011)
*melanogaster *spp.		0.018 (1.10 × 10^-6^)	0.070 (1.90 × 10^-4^)	0.477 (0.013)
*Micropia*	0.467 (0.410-0.527)			
*Zaprionus *× *melanogaster *spp.		0.045 (1.44 × 10^-5^)	0.124 (1.94 × 10^-4^)	0.524 (0.015)
*Zaprionus *spp.		0.026 (3.80 × 10^-5^)	0.040 (2.45 × 10^-4^)	0.579 (0.019)
*melanogaster *spp.		0.028 (6.45 × 10^-5^)	0.068 (4.87 × 10^-4^)	0.388 (0.005)
*Gpdh*	0.602 (0.492-0.700)			
*Zaprionus *× *melanogaster *spp		0.009 (3.07 × 10^-6^)	1.012 (0.004)	0.009 (4.90 × 10^-6^)
*Zaprionus *spp.		0.005 (2.16 × 10^-5^)	0.150 (0.002)	0.041 (5.43 × 10^-4^)
*melanogaster *spp.		0	0.193 (0.003)	0

Note: Pairwise dS and dN distances in Additional files 3 and 6.

**Figure 3:**
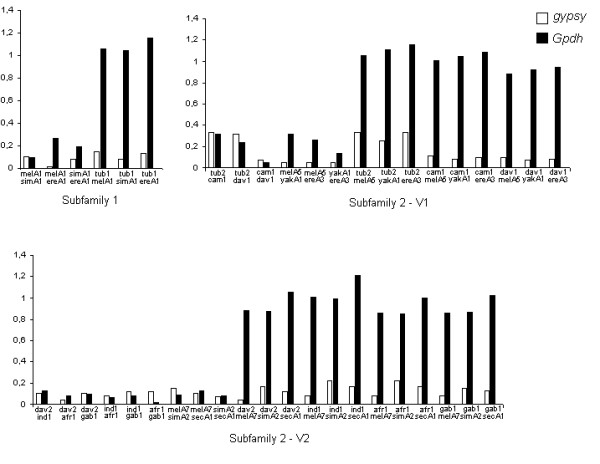
**Comparative analysis of dS values of *Gypsy *and *Gpdh***. Comparative analyses of the dS values between *Gypsy *and *Gpdh *sequences of *Zaprionus *and *melanogaster *species within the S1 subfamily, S2V1 and S2V2. tub: *Z. tuberculatus*, cam: *Z. camerounensis*, dav: *Z. davidi*, gab: *Z. gabonicus*, afr: *Z. africanus*, ind: *Z. indianus*, mel: *D. melanogaster*, sim: *D. simulans*, sec: *D. sechellia*, yak: *D. yakuba*, ere: *D. erecta*.

In the absence of intra-clade selective constraints, the comparisons between the dS values of the TE and a host gene permit evaluation of the hypothesis of ancestral polymorphism within subfamilies. If the *Gypsy *retroelement is evolving vertically and the incongruence in the phylogeny are due to differential subfamily distribution in the analyzed species, the dS pairwise comparisons between the TE and the host gene would be expected to be equivalent. But, if the dS of TE is lower than the dS of host gene under similar or higher levels of selective constraints and in the absence of strong codon usage bias, HT events can be inferred. Using this approach, the dS of *Gypsy *and *Gpdh *gene, as well as the Codon Bias Index (CBI) were compared for one clone representing each species of *Zaprionus *and the best Blastn subject of the genomic sequences in each clade. Codon usage bias is a factor that may be responsible for low dS values for TE sequences, since there is a negative correlation between codon usage bias and dS values [20]. The Codon Bias Index (CBI) indicates that the *Gypsy *and *Gpdh *sequences did not suffer high codon bias, although the mean value of 0.602 for *Gpdh *suggests that the small difference in magnitude of dS between *Gpdh *and *Gypsy *ought to be taken cautiously in inferring HT, since the variation in the *Gpdh *dS distance could reflect codon usage. Hence, comparisons between *Gypsy *and *Gpdh *dS were used to infer HT only if the dS values of *Gypsy *were at least twice as low as those of *Gpdh*. The *Gypsy *dS values between *Zaprionus *and *melanogaster *species ranged from 3 (*Z. tuberculatus*2 vs *D. melanogaster*5 and *D. erecta*3) to 20 times (*D. melanogaster*7 vs *Z. davidi*2) lower than those of *Gpdh *(Figure 3). On the other hand, comparisons of dS between the *Zaprionus *species within each clade gave *Gypsy *dS values approximately equal or higher than *Gpdh *dS, excepting for *Z. davidi2 *vs *Z. indianus1 *and *Z. africanus1 *which presented dS of *Gypsy *slightly lower than the host gene ones. These results may indicate that the *Gypsy *retroelement evolved mainly by vertical transmission within the *Zaprionus *subgenus, but there may have been HT events between the *Zaprionus *and the *melanogaster *species what could reject the hypothesis of ancestral polymorphism within the clades.

### Phylogenetic relationships of the *Micropia *retroelement

In order to evaluate the evolutionary history of *Micropia*, the same approaches of phylogeny and divergence estimations used for *Gypsy *were applied. Sequences homologous to the *Zaprionus *species were also identified only in species of the *melanogaster *subgroup, except for *D. erecta*, the unique species of the *melanogaster *subgroup for which no significant matches were obtained. *D. sechellia *had the most hits (22), followed by *D. simulans *(six), *D. yakuba *(four) and *D. melanogaster *(three) [Additional file 3]. Structure analysis indicates that the *D. melanogaster *and *D. simulans *genomes harbor putatively full-length insertions [Additional file 8].

#### Inferring phylogenetic relationships and divergence

**Figure 4:**
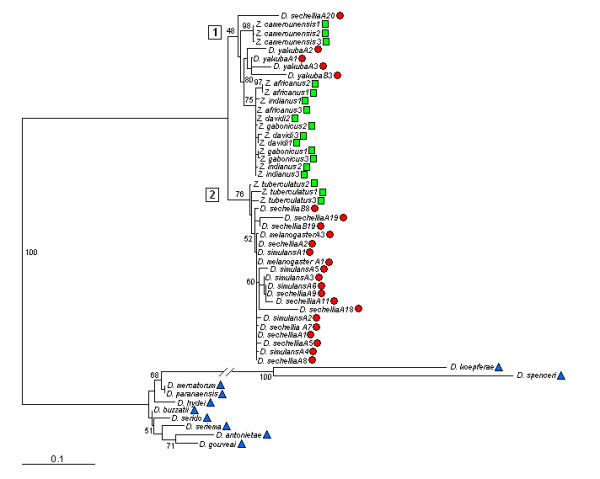
**Phylogenetic relationships between *Micropia *retroelements**. Phylogeny of *Micropia *sequences of genus *Zaprionus *(green squares) and the *melanogaster *(red circles) and *repleta *(blue triangles) groups. The trees were reconstructed using the maximum-likelihood method (HKY85 distance), as implemented in the PhyML program. Numbers in squares indicate the clade number mentioned in the text. The branch support was calculated using bootstrap test (1000 replications). Numbers next to species names indicate the clone or genomic sequence identification.

The MP, NJ and ML reconstructions of the *Micropia *sequences from the *Zaprionus *subgenus, the genomic sequences from the *melanogaster *subgroup and the GenBank sequences of the *repleta *group had similar topologies. As for *Gypsy*, only ML reconstruction is given (Figure 4). The tree exhibits several incongruences according to species phylogeny, such as grouping species in the *Zaprionus *genus together with those of the *melanogaster *subgroup. The sequences of the *Micropia *family of the *repleta *group were clustered outside a well-supported branch that harbors all sequences of the *Zaprionus *subgenus and the *melanogaster *species subgroup, constituted by two clades. Clade 1, with low support (48%), grouped the *D. sechellia*A20 sequence with three internal clades, corresponding to the *Z. camerounensis *sequences (bootstrap 98%), *D. yakuba *(no support) and the sequences of *Z. davidi*, *Z. indianus*, *Z. africanus *and *Z. gabonicus *(bootstrap 75%). This last clade did not give a clear internal species-specific clustering. Clade 2 grouped sequences of the *melanogaster *subgroup (from *D. melanogaster*, *D. simulans *and *D. sechellia*) together with *Z. tuberculatus *in 76% of the replications. Despite the clustering of all *melanogaster *sequences in clade 2, the support was not high (52%). The mean divergence within clade 1 was much lower (0.009) than in clade 2 (0.051) or between them (0.074) [Additional file 9]. Analyzing the species groups separately, the mean divergence of the *Zaprionus *sequences was 0.033; it was 0.047 within the *melanogaster *species subgroup, and 0.073 between the *Zaprionus *and the *melanogaster *sequences (Table 2). The phylogenetic tree and distance estimates <20% indicate that *Micropia *in the *Zaprionus *and *melanogaster *species belong to the same subfamily.

#### Testing the HT hypothesis

Evaluation of the selective constraints showed that the mean dN:dS values of the *Micropia *sequences were 14-58 times higher than those of *Gpdh *(Table 3 and [Additional file 10]). Between the *Zaprionus *and *melanogaster *sequences, for example, the values were 0.524 and 0.009 for *Micropia *and *Gpdh*, respectively. In addition, the Z-test failed to reject the neutrality hypothesis in ~80% of the pairwise comparisons [Additional file 11]. These results refute the hypothesis that the high similarity between the *Micropia *sequences of *Zaprionus *and *melanogaster *is due to high selective constraints conserving their nucleotide sequences.

**Figure 5:**
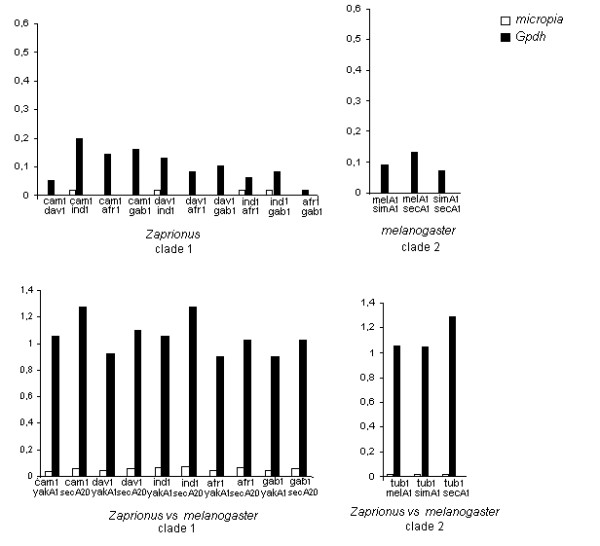
**Comparative analysis of dS values of *Micropia *and *Gpdh***. Comparative analyses of the dS values between *Micropia *and *Gpdh *of *Zaprionus *and *melanogaster *species. Comparison with only black column indicates that the synonymous sites of *Micropia *are invariable. tub: *Z. tuberculatus*, cam: *Z. camerounensis*, dav: *Z. davidi*, gab: *Z. gabonicus*, afr: *Z. africanus*, ind: *Z. indianus*, mel: *D. melanogaster*, sim: *D. simulans*, sec: *D. sechellia*, yak: *D. yakuba*.

To evaluate the ancestral polymorphism hypothesis for *Micropia*, the dS values of *Micropia *RNAseH and *Gpdh *sequences were compared between the sequences of *Zaprionus *and *melanogaster *species in each clade (Figure 5). All the dS distances for the *Micropia *were lower than those for the *Gpdh*. For instance, the dS between *Z. tuberculatus *and the *melanogaster *subgroup sequences in clade 1 were from 61 (vs *D. simulans*) to 76 (vs *D. sechellia*) times lower than that those for the *Gpdh*. Those for the *D. yakuba*A1 sequence were from 18 (vs *Z. indianus*1) to 31 (vs *Z. camerounensis*1) times lower. In addition, dS was zero in some comparisons, e.g. those among the *Z. camerounensis*, *Z. davidi, Z. africanus *and *Z. gabonicus *sequences of clade 1. The high conservation in the synonymous sites of the RNAseH sequences favors the hypothesis that their low divergence is due to recent acquisitions of *Micropia *in the *Zaprionus *and *melanogaster *genomes, and that HT has shaped its evolution, as already proposed for the *Gypsy *retroelement.

### Network trees corroborate the inference of HT

Network reconstructions were used as an additional approach to test the hypothesis that HT has shaped the evolution of *Gypsy *and *Micropia *in *Zaprionus *and *melanogaster *species. Since TE sequences can be considered as populations of sequences that share a common ancestor, this type of phylogeny lets one propose possible donors and receptors species, and to reconstruct the TE dispersion routes by the proposition of the ancestral nodes.

**Figure 6:**
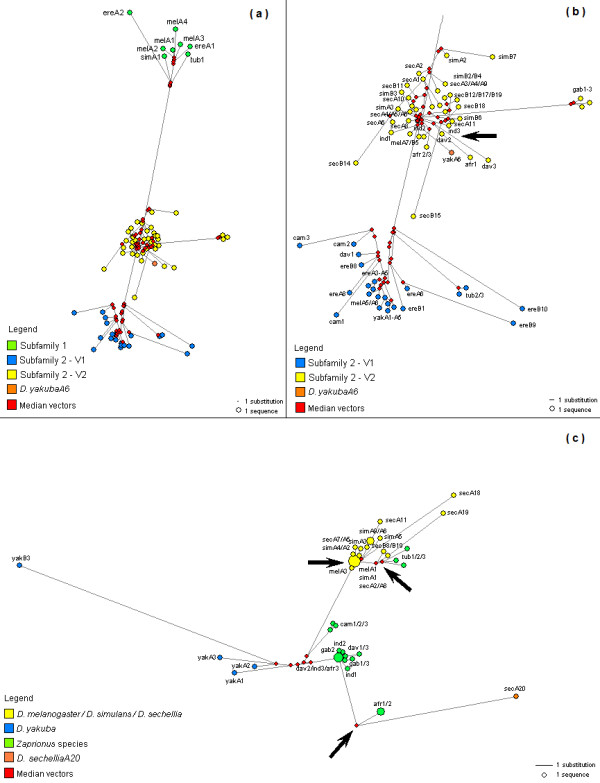
**Network reconstructions**. Median-joining network analyses for the *Zaprionus *and the *melanogaster *species. The size of each circle denotes the number of sequences grouped together. (a) Network for the *Gypsy *retroelement. Each group obtained in the phylogenetic tree is differently colored. (b) Detail of the *Gypsy *Subfamily 2. (c) Network for the *Micropia *retroelement. The sequences are colored according the clustering in the phylogenetic tree. The black arrows show the HT events evidenced by network trees.

The *Gypsy *network indicated three clusters of sequences (Figure 6a), which correspond to the S1 subfamily and the two variants of the S2 subfamily (S2V1 and S2V2). Moreover, the S1 subfamily was closer to S2V2 than to S2V1, which indicates that S2V1 diversified from S2V2. The presence of median vectors - that correspond to a hypothetical (in theory ancestral) or unsampled sequences - connecting the *Zaprionus *and *melanogaster *sequences suggests that the HT events occurred in the ancestors of the sequences sampled, indicating that the lateral transmissions were not recent. The network tree allowed us to see a possible HT not clearly showed by the traditional phylogeny, but evident from the dS comparisons. The *Z. davidi*2 sequence is the ancestral node of the *Z. africanus*1 sequence (Figure 6b), suggesting a single HT event from *Z. davidi *to *Z. africanus*. The network reconstruction also let the *D. yakuba*A6 sequence be included as a S2V2 member.

Network reconstruction for *Micropia *clustered the sequences of *D. yakuba*, *D. melanogaster*, *Z. camerounensis*, *Z. davidi*, *Z. indianus *and *Z. africanus *with central median vectors, suggesting that high sequence conservation was not due to HTs involving the species analyzed, but probably one or more donor species not sampled (Figure 6c). The *D. melanogaster*A3 sequence connects the median vectors with a large cluster that harbors *D. sechellia*, *D. simulans *and *D. melanogaster *sequences, which in turn links a median branch to the *Z. tuberculatus *sequences. This arrangement indicates at least two more recent transfers, one from *D. melanogaster *to *D. simulans *and/or *D. sechellia *or even to the ancestor of those sister species (corroborated by the low dS values), and a second from any of these species to *Z. tuberculatus*. In addition, the branch of the *D. sechellia*A20 sequence with a median vector that links the *Z. camerounensis*, *Z. davidi*, *Z. indianus *and *Z. africanus *sequences suggest an additional HT involving the ancestor of *D. sechellia*. However, the absence of geographic sharing between *D. sechellia *and *Zaprionus *species means that this proposal should be viewed cautiously. An alternative explanation could be a high rate of evolution of the *D. sechellia*A20 sequence, followed by random convergent evolution.

### HT inference and estimation of the divergence time between TE sequences

To reinforce the HT hypothesis, the divergence times of both retroelements were calculated using the molecular clock (see Material and Methods). The divergence estimates for *Gypsy *sequences showed that the HTs may have started around 11.2 MYA (Table 4). Regarding the S1 subfamily, two HTs can be proposed. The first may have occurred from a *D. melanogaster*/*D. simulans *ancestor to *Z. tuberculatus *about 3.8-8.8 MYA, prior to the time of *D. melanogaster*/*D. simulans *splitting (2-3 MYA [2]). This event was followed by a transfer between *D. melanogaster *and *D. erecta *(up to 3.1 MYA), species that share a common ancestor at 8-15 MYA [2]. Although an alternative hypothesis of HT between *D. simulans *and *D. erecta *can be formulated on the basis of the phylogeny branching, the greater difference in dS comparisons between the pair *D. erecta*/*D. melanogaster *than the pair *D. erecta*/*D. simulans *(Figure 3) reinforces the first hypothesis.

**Table 4:** Time of divergence and horizontal transfer events identified for the *Gypsy *and *Micropia *retroelements

Species 1	Species 2	MYA
*Gypsy*		
Subfamily 1		
*D. melanogaster*/D. simulans*	*Z. tuberculatus*1	3.8 - 8.8
*D. melanogaster*	*D. erecta*	0 - 3.1
Subfamily 2 - V1		
*D. erecta*	*Z. tuberculatus*	5.2 - 11.2
*D. erecta*	*Z. davidi*/Z. camerounensis*	1.2 - 6.0
*D. erecta*	*D. yakuba*	2.5 - 3.8
*D. erecta*	*D. melanogaster*	2.5 - 3.9
Subfamily 2 - V2		
*D. melanogaster*/*D. simulans*/D. sechellia*	*indianus *complex/Z. davidi*	0.6 - 10.3
*Z. davidi*	*Z. africanus*	1.9^#^
*Micropia*		
?	*D. melanogaster*	0
?	*D. yakuba*	2.3 - 3.2
?	*indianus *complex	0 - 1.6
?	*Z. davidi*	0
?	*Z. camerounensis*	0
*D. melanogaster*	*D. simulans/D. sechellia*	0^#^
*D. melanogaster *or D. simulans/D. sechellia*	*Z. tuberculatus*	0.8 - 1.5^#^

Symbols: *: Species ancestor. ^#^: Time of divergence calculated using dS pairwise comparisons between sequences connected by the network phylogeny.

The *Gypsy *S2V1 sequences seem to have been involved at least in four HT events. We suggest that *D. erecta *was the donor of S2V1 in a more ancient transfer to *Z. tuberculatus *(occurred about 5.2-11.2 MYA), followed by introduction into the *Z. camerounensis*/*Z. davidi *ancestor (1.2-6 MYA), and lately into *D. yakuba *(2.5-3.8 MYA) and *D. melanogaster *(2.5-3.9 MYA). The transfer to *Z. camerounensis*/*Z. davidi *probably occurred in their ancestor, because these species diverged around 2.2 MYA [1], but the hypothesis of two independent HTs cannot be disregarded. The donor status of *D. erecta *was based on their basal positioning in the network, the repeated clustering to all the other species of this variant in the phylogeny, the divergence times between *D. erecta *and *D. melanogaster*/*D. yakuba *(8-15 MYA [2]).

The S2V2 variant is probably involved in one HT event that happened around 0.6-10.3 MYA. It could have occurred between the ancestor of the modern sequences of *D. melanogaster*/*D. simulans*/*D. sechellia *and the *indianus*/*davidi *complexes. Alternatively, the HT could have happened in each complex separately, since the *davidi *complex diverged about 0.8 MYA [1]. The HT between the *D. melanogaster*/*D. simulans*/*D. sechellia *ancestor and the *indianus *complex ancestor is a broader scenario than the HT proposed between *Z. indianus *and *D. simulans *[18], exemplifying the importance of analyzing as many species as possible before inferring horizontal transfers [22]. Finally, the time of divergence also corroborates the HT from *Z. davidi *to *Z. africanus *S2V2 sequences, as seen in the network tree, which probably occurred ~1.9 MYA, subsequent to the divergence of these species (2.6 to 4.1 MYA [1]).

Comparing the divergence times between the *Micropia *retroelement of the *Zaprionus *and *melanogaster *sequences with those of *Gypsy*, the HTs of *Micropia *might have started more recently than those of *Gypsy *(*Micropia *HTs: 3.2 MYA, *Gypsy *HTs: 11.2 MYA). The invariance of the synonymous sites between several pairwise comparisons [Additional file 8] makes it difficult to date these events, but the highest dS levels indicate that, of the species we have analyzed, *D. yakuba *and the *indianus *complex species were the first to be invaded by *Micropia*. So, the evolutionary analyses lead to the inference that *Micropia *diverged after the splitting of their species hosts, suggesting that at least an unknown species might have transferred *Micropia *sequences independently in five HTs to *D. melanogaster*, *D. yakuba *(2.3-3.2 MYA), *Z. camerounensis*, *Z. davidi *and the *indianus *complex (up to 1.6 MYA). Species of neither the *repleta *nor the *cardini *species group are good candidates, since they harbor *Micropia *retrotransposons of a different family [29,30]. Hence, the probable donor(s) might have been a non-studied species. Based mainly on the network tree, two other HT events can be proposed. *D. melanogaster *may have been the donor of *Micropia *to the *D. simulans *and *D. sechellia*, or even to their ancestor. However, introgression cannot be excluded in this case. In turn, one of these three species might have been involved in the transmission of *Micropia *to *Z. tuberculatus*, about 0.8-1.5 MYA.

## Discussion

This survey of the *Gypsy *and *Micropia *in seven species of *Zaprionus *indicates that they are widely distributed and transcriptionally active only in species of subgenus *Zaprionus*. Additionally, the evolutionary analyses demonstrate that these two retroelements are closely related to those of the *melanogaster *species subgroup, and that their histories might have been repeatedly marked by events of inter- and intra-subgenus HTs. Several pieces of evidence have shown that the phenomenon of HT is frequent in eukaryotes [22,37-39]. It is important to note that the HTs proposed here were assumed only when the species involved in the event shared geographic, temporal and ecological environments, and when three separate pieces of evidence suggested their occurrence: (i) lower dS values between the TE sequences than the *Gpdh *sequences, (ii) incongruence between host and TE phylogenies, and (iii) last common ancestor of TE more recent than that of the species.

The studies of *Micropia *and *Gypsy *in subgenus *Zaprionus *species highlight the HT events as an alternative evolutionary mechanism of the retroelements evolution. Among the HTs inferred for *Gypsy*, four events seemed to involve the *Zaprionus *and *melanogaster *species, three involved only the *melanogaster *group species, and only one the *Zaprionus *species. For the *Micropia *retrotransposon, seven HTs were suggested: five between unknown donor(s) and the *Zaprionus *or the *melanogaster *species, one among the *melanogaster *species, and one between the *Zaprionus *and the *melanogaster *species. The 15 HTs we have proposed probably occurred during two different waves of invasions. First, the *Gypsy *retroelement of the *melanogaster *subgroup had been introduced in the *Zaprionus *subgenus ~11 MYA. After that (~3 MYA), a second HT wave involving the *Micropia *retroelement may have introduced it into both the *Zaprionus *subgenus and *melanogaster *subgroup from an unknown donor. After the introduction, the TEs could have been prone to transposition and re-introduction in other related species. For example, *Z. davidi *has donated their *Gypsy *sequence to *Z. africanus *after had received it from an ancestor of *D. melanogaster*, *D. simulans *and *D. sechellia*.

According to the TE evolutionary cycle, the TE history could start from HT events, followed by the initial transpositional burst in the new host, and then the accumulation of defective copies along with host-directed epigenetic silencing [40]. The last step could lead to the loss of mobility and, finally, to molecular erosion by random mutations. The high frequency of HTs, combined with the transcriptional activity and low insertion numbers of *Gypsy *and *Micropia *retroelements in the *Zaprionus *species subgenus indicates that these elements remain at the first or second steps of the evolutionary life history of the TEs, i.e. the invasion or the genomic spread period. A question that arises is whether these elements have been transferred in similar ways. HTs of *Gypsy *are easier to explain, since the active *Gypsy env *can enable its dispersion and expansion of the genomic territory, but this mechanism does not to apply to *Micropia*, which lacks *env*. Nevertheless, retroelements could have been transferred concomitantly with retrovirus infections, even *Gypsy*; or parasitic infestations, as for example by intracellular *Wolbachia *bacteria or mites [22]. Moreover, our data allowed us to propose there were HT of *Gypsy *and *Micropia *among sister species such as *D. melanogaster*, *D. simulans *and *D. sechellia*, although introgression could not be ruled out.

Another concern is the evolutionary process of fixation of the TEs after the horizontal introduction. These elements could share similar mechanisms of regulation, which would permit them escaping from selective forces and allow fixation after invasion. Control of *Drosophila *retroelement mobilization depends on the transcription rate, which is directly related to the presence of specific regulatory proteins [41-45] and the rate of RNA degradation, mediated by RNAi systems [46]. However, genetic information about the species of the genus *Zaprionus *is scarce. Further, studies of the *Zaprionus *genes involved in controlling mobilization could lead to hypotheses about the mechanisms that allow highly related sequences to be shared in the *Zaprionus *and *melanogaster *species genomes.

## Conclusion

Our survey suggests that *Zaprionus *species have experienced waves of retroelement invasions, particularly during the last 7 million years. Species of the *melanogaster *subgroup might have donated their *Gypsy *sequences to species of the *Zaprionus *subgenus, and these two species groups could have received the *Micropia *retroelement from one or more unknown donor species. After the initial introduction, these species could share their elements. Since the *Zaprionus *genus and the *melanogaster *subgroup seem to share the same age of origin and diversification in tropical Africa, as well as ecological features, our data suggest that they passed through a permissive period of transposable element invasion during the diversification period.

## Methods

### Fly stocks and DNA extraction

All the strains of the *Zaprionus *species that were used are listed in Table 1, being derived from a single female selected randomly from a mass culture kindly provided by Drs Jean David and Amir Yassin from LEGS, CNRS, France. The *D. melanogaster *Canton-S strain was used as positive control for the molecular analyses. Genomic DNA was extracted from 10 individuals of each strain using the phenol-chloroform method [47].

### PCR reactions, cloning and sequencing

Primers 2813 (5' TTA ACT CCT AGA GTT CAT CGC TGG 3') and 2814 (5' CAT GTA CCT GGT TAA CTA CTG ACC 3') were used to amplify the equivalent 386 bp fragment of the *D. hydei Micropia *retroelement located in a highly conserved region of the RNAseH domain, between nucleotides 2813 and 3198 [31]. The *Gypsy *retroelement fragment was obtained using primers GYP3S2F (5' AAA GGC GAY TTG GTT GAC ACT CC 3') and GYP3S2R (5' CAR GTG GCT RGG TTG RGT GTG 3') and corresponds to a 485 bp sequence located in the 6491-6511 region of *env *(ORF 3) in the *D. melanogaster Gypsy *element [18]. Both PCR reactions were performed in a final volume of 25 μl, using 200 ng of genomic DNA, 0.4 μM of each primer, 160 μM of each dNTP, 2 mM MgCl_2 _and 1 U Taq Platinum polymerase (Invitrogen) in 1× PCR buffer. The *Gypsy *cycling parameters used for amplification have been described previously [18]. The *Micropia *PCR cycling parameters were: 94°C for 3 min for initial denaturation, 40 cycles of 94°C for 30 s, 58°C for 1 min, and 72°C for 1 min, followed by a final extension step at 72°C for 10 min. The fragments obtained were purified directly from the PCR product, using the GFX PCR DNA and Gel Band Purification Kit (GE Healthcare), and cloned with the TOPO TA Cloning Kit (Invitrogen). Three randomly chosen clones were automatically sequenced in an ABI PRISM 3100 Genetic Analyzer (Applied Biosystems/Hitashi) using the primer pair T7 and M13R.

### RNA extraction and RT-PCR reactions

Heads and gonads from 10 individuals of each sex were dissected in Testis Buffer (183 mM KCl, 47 mM NaCl and 10 mM Tris-HCl pH 6.8). Total RNA was isolated from the dissected tissues using the TRIZOL reagent (Invitrogen) method and genomic DNA was eliminated from the samples by RQ1 RNase-Free DNase (Promega) according to the manufacturer's instructions. The cDNA pool was generated from the total RNAs using random primers and a High Capacity cDNA Archive Kit (Applied Biosystems) under low stringency conditions (37°C). The PCR reaction conditions and cycling parameters were the same as those used for genomic DNA amplification of both retroelements. To test genomic DNA contamination in the total RNA and cDNA quality *Gpdh *constitutive gene amplifications were performed using the total RNA extract treated with DNAse and the cDNA pool as templates, respectively. The control PCR reactions were carried out using 200 ng of total cDNA, 0.1 mM of each dNTP, 0.4 μM of the primers ZapGPDHF (5' GTT CGG CAA TTG AAC CAA TG 3') and ZapGPDHR (5' AGA GAG TCC GTG TGC ATG TG 3'), 2 mM MgCl_2 _and 1 U Taq Platinum polymerase (Invitrogen) in 1× PCR buffer. The cycling parameters were: 94°C for 2 min for initial denaturation, 35 cycles of 94°C for 1 min, 60°C for 1 min and 72°C for 1 min, and an additional extension step at 72°C for 10 min. The *Gpdh *primers were designed based on the *Z. tuberculatus Gpdh *(Glycerol 3 phosphate dehydrogenase) gene ORF1 (L37039) and amplify a 337 bp sequence in this sequence.

### Phylogenetic analyses

*Zaprionus *subgenus sequences were manipulated into BioEdit [48] and aligned with Clustal W 1.81 [49]. The most divergent clones (>25%) were selected as queries for a search in the 12 *Drosophila *genomes using the flybase BLASTn tool http://flybase.bio.indiana.edu/blast/ and the genome database released on October 17^*th*^, 2008. The queries selected were *Z. tuberculatus*1, *Z. tuberculatus*2 and *Z. gabonicus*1 for *Gypsy*, and *Z. tuberculatus*2 and *Z. africanus*1 for *Micropia*. The structure of the genomic sequences were predicted using the BLAST 2 sequences and ORF Finder programs. In order to obtain only highly related and non-disrupted sequences the searches were performed with stringent parameters (e-values > e-^50 ^and 80% coverage). Redundant genomic sequences (100% identical) were not included in the phylogenetic analyses in order to minimize polytomies.

Multiple alignments of the *Zaprionus *and the genomic sequences were used to infer the phylogenetic relationships, using the maximum likelihood (ML), neighbor-joining (NJ) and maximum parsimony (MP) methods, as implemented in PhyML 3.0, MEGA 4.1 and PAUP v.4.0b10 [50-52], respectively. Branch support was calculated by bootstrap analysis consisting of 1000 replicates [53]. In the NJ and ML analyses, maximum composite likelihood (MCL) and HKY85 distances were used to estimate the divergence matrices and reconstructed trees, respectively [54,55]. A heuristic search algorithm was used for MP reconstruction. The sequences obtained were registered in the GenBank database (Table 1). *Gypsy *sequences of *S. latifaciaeformis *(AF548144, AF548153 and AF548152) and *D. willistoni *(AF548159, AF548176 and AF548143), and *Micropia *sequences of *D. buzzatii *(AY522351), *D. hydei *(AY519123), *D. paranaensis *(AY519124), *D. mercatorum *(AY519125), *D. seriema *(AY522346), *D. gouveai *(AY522353), *D. antonietae *(AY522345), *D. serido *(AY522344), *D. spenceri *(AY522347) and *D. koepferae *(AY522352) from GenBank, were also used in the phylogenetic reconstruction.

### Selection tests for *Gypsy*, *Micropia *and host gene

The number of synonymous substitutions per synonymous site (dS), non-synonymous substitutions per non-synonymous site (dN), the Codon Based Z-test and dN:dS ratios were estimated for the *Zaprionus *and *melanogaster *sequences using the Nei-Gojobori distance with the Jukes and Cantor correction, as implemented in MEGA 4.1 [51]. The *Gypsy *sequences *D. sechellia*A6/B14/B15, *D. yakuba*A6 and *D. erecta*A2/A6/B9, and the *Micropia *sequences *D. simulans*A3/A6, *D. sechellia*A9/A18/A20 and *D. yakubaA3*, were excluded from those alignments because of the presence of indels (>2 bp) disrupting the open reading frames. Point (1 bp) alignment gaps were deleted prior to estimation of dN and dS. The codon bias index (CBI, [56]) was estimated for each sequence using the DnaSP 4.50 program [57], where zero indicates no bias and 1 maximum bias. Sequences of exon 4 of *Gpdh *in *Zaprionus *and *melanogaster *subgroup species (FJ705445 to FJ705450, L37039, NM_057218, XM_002078253, XM_002089126, XM_001968825 and *D. sechellia *genomic sequence: scaffold_5/4016995-4017372) were used to compare the dS values of the *Gypsy *and *Micropia *sequences.

### Estimations of divergence time for *Gypsy *and *Micropia*

The time of divergence of *Gypsy *and *Micropia *retrotransposons was estimated according to the molecular clock equation *r = k/2T*, where *r *is the evolutionary rate (0.011 per site per MY, according to synonymous sites rate for genes with low codon bias in *Drosophila *[11]), *k *is the dS pairwise divergence, and T is the divergence time between species.

### Median-joining networks

The median-joining network trees were obtained using all sequences studied in the conventional phylogenies. The reconstructions were performed using DNA Aligment 1.3.0.1 and NETWORK 4.5.1.0 software [58], available at the Fluxus Technology Ltd. website. 'MP calculation' was applied for post-processing the networks, the characters were equally weighted, and the other median-joining parameters followed the software default.

## List of abbreviations

HT: horizontal transfer; LTR: long terminal repeat; TE: transposable element; *env*: envelope; MCL: maximum composite likelihood distance; S1: *Gypsy *subfamily 1; S2V1: *Gypsy *subfamily 2 variant 1; S2V2: *Gypsy *subfamily 2 variant 2; CBI: codon bias index; *Gpdh*: glycerol 3 phosphate dehydrogenase; dS: number of synonymous substitutions per synonymous site; dN: non-synonymous substitutions per non-synonymous site; ML: maximum likelihood; NJ: neighbor-joining; MP: maximum parsimony.

## Authors' contributions

NS designed the study and wrote the manuscript. MAVS and PC provided laboratory facilities for experimental analyses and participated in data interpretation. CMAC conceived and supervised the project, participated in data interpretation and wrote the manuscript. All authors read and approved the final manuscript.

## Supplementary Material

Additional file 1 **Transcriptional activity of *Gypsy *and *Micropia *retroelements**. RT-PCR was used to verify the transcriptional activity of the *Gypsy env *and the *Micropia *RNAseH genes in ovaries (O), testes (T) and heads (H) of *Zaprionus *species. -: negative control with ultrapure water; +: positive control with *D. melanogaster *genomic DNA. *Gpdh *amplification was used for total RNA quality control. Click here for file

Additional file 2 **Insertion numbers of *Gypsy *and *Micropia *retrotransposons in *Zaprionus *species**. The insertion numbers were indicated in the bottom of the figure. Southern blotting analyses were performed using 5 μg of genomic DNA double digested with restriction endonucleases BamHI and MluI and probes derivate from the *Gypsy env *and *Micropia *RNAseH fragments of *Z. indianus*, random primers [α-P32]dCTP labelled. The nylon membranes were hybridized at 65°C and washed twice with 2× SSC and 0.1% SDS, and once with 0.2× SSC and 0.1% SDS for 20 min at the hybridization temperature. mel: *D. melanogaster*, mul: *Z. multistriatus*, tub: *Z. tuberculatus*, cam: *Z. camerounensis*, dav: *Z. davidi*, gab: *Z. gabonicus*, afr: *Z. africanus*, ind: *Z. indianus*. Click here for file

Additional file 3 **Description of the genomic sequences from the 12 *Drosophila *genome searches**. Chromosome and nucleotide location of the *Gypsy *and *Micropia *insertions from the *Drosophila *genomes. Click here for file

Additional file 4 **Structure of the *Gypsy *retroelement in the *melanogaster *subgroup genomes**. *Gypsy *insertions in *D. melanogaster *(mel), *D. simulans *(sim), *D. sechellia *(sec), *D. yakuba *(yak) and *D. erecta *(ere). The sequences represented have at least 80% identity with the canonical element of *D. melanogaster *(AF033821), which is the first schematic representation. Black rectangles - long terminal repeats. Gray rectangles - coding regions. Asterisk - genomic sequences interrupted by the scaffold ends or Ns. Striped rectangles - region used in the phylogenetic analyses. Click here for file

Additional file 5 **Pairwise genetic distance among *Gypsy *sequences of *Zaprionus*, *melanogaster*, *D. willistoni *and *S. latisfasciaeformis***. Distances calculated by the MCL method as implemented by MEGA 4.1. The sequences were clustered according the phylogenetic clades. Click here for file

Additional file 6 **dN (below) and dS (above) values of pairwise comparisons among *Zaprionus *and *melanogaster Gypsy *sequences**. Distances calculated by Nei-Gojobori method (Jukes-Cantor's correction), as implemented by MEGA 4.1. Click here for file

Additional file 7 **Z values (above) and significance P-values (below) for the Codon-Based Z-test of neutrality between *Gypsy *sequences of *Zaprionus *and *melanogaster *species**. Test performed using alternative hypothesis of non-neutrality (dN ≠ dS) and Nei-Gojobori distance (Jukes-Cantor's correction). Gray cells correspond to significant (p < 0.05) pairwise comparisons. Click here for file

Additional file 8 **Structure of the *Micropia *retroelement in the *melanogaster *subgroup genomes**. *Micropia *insertions in *D. melanogaster *(mel), *D. simulans *(sim), *D. sechellia *(sec) and *D. yakuba *(yak). The sequences represented have at least 80% identity with the canonical element of *D. melanogaster *(X14037), which is the first schematic representation. Black rectangles - long terminal repeats. Gray rectangles - coding regions. Asterisk - genomic sequences interrupted by the scaffold ends or Ns. Striped rectangles - region used in the phylogenetic analyses. Click here for file

Additional file 9 **Pairwise genetic distance among *Micropia *sequences of *Zaprionus*, *melanogaster *and *repleta *species**. Distances calculated by the MCL method as implemented by MEGA 4.1. Click here for file

Additional file 10 **dN (below) and dS (above) values of pairwise comparisons between *Micropia *sequences of *Zaprionus *and *melanogaster *species**. Distances calculated by the Nei-Gojobori method with Jukes-Cantor's correction as implemented by MEGA 4.1. Click here for file

Additional file 11 **Z values (above) and significance P-values (below) for the Codon-Based Z-test of selection between *Micropia *sequences of *Zaprionus *and *melanogaster *species**. Test performed using alternative hypothesis of non-neutrality (dN ≠ dS) and Nei-Gojobori distance (Jukes-Cantor's correction). Gray cells correspond to significant (p < 0.05) pairwise comparisons. Click here for file
