# Supplementary figures and images for: Multiple invasions of Gypsy and Micropia retroelements in genus Zaprionus and melanogaster subgroup of the genus Drosophila
Source: BMC Evol Biol. 2009 Dec 2;9:279. doi: 10.1186/1471-2148-9-279 (PMC2797524; doi:10.1186/1471-2148-9-279)

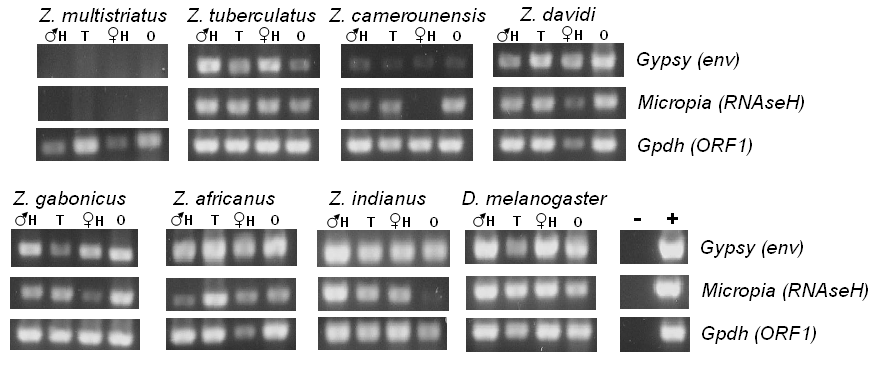

Supplement: Additional file 1 — Transcriptional activity of Gypsy and Micropia retroelements. RT-PCR was used to verify the transcriptional activity of the Gypsy env and the Micropia RNAseH genes in ovaries (O), testes (T) and heads (H) of Zaprionus species. -: negative control with ultrapure water; +: positive control with D. melanogaster genomic DNA. Gpdh amplification was used for total RNA quality control. [file 1471-2148-9-279-S1.TIFF]

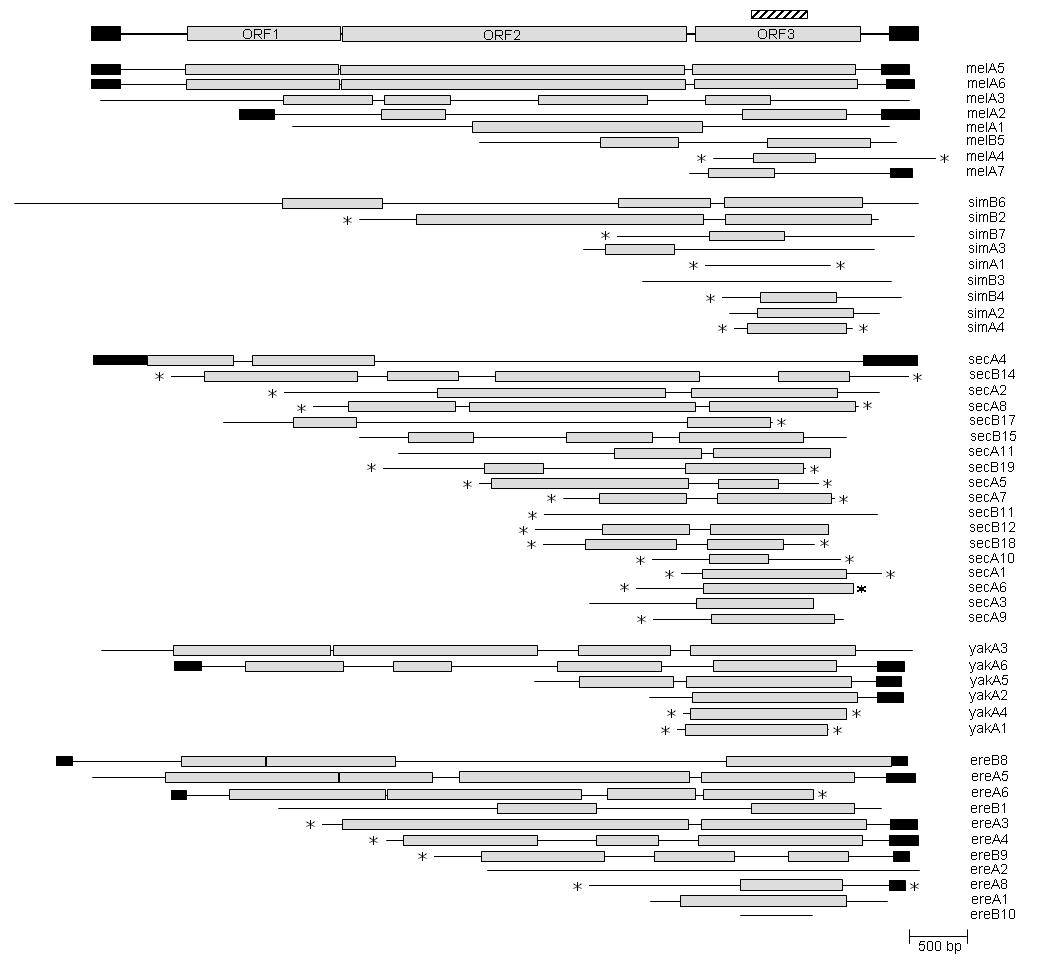

Supplement: Additional file 4 — Structure of the Gypsy retroelement in the melanogaster subgroup genomes. Gypsy insertions in D. melanogaster (mel), D. simulans (sim), D. sechellia (sec), D. yakuba (yak) and D. erecta (ere). The sequences represented have at least 80% identity with the canonical element of D. melanogaster (AF033821), which is the first schematic representation. Black rectangles - long terminal repeats. Gray rectangles - coding regions. Asterisk - genomic sequences interrupted by the scaffold ends or Ns. Striped rectangles - region used in the phylogenetic analyses. [file 1471-2148-9-279-S4.TIFF]

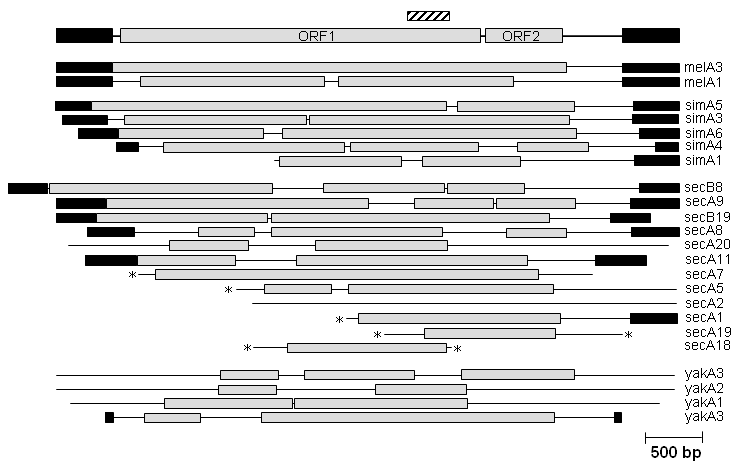

Supplement: Additional file 8 — Structure of the Micropia retroelement in the melanogaster subgroup genomes. Micropia insertions in D. melanogaster (mel), D. simulans (sim), D. sechellia (sec) and D. yakuba (yak). The sequences represented have at least 80% identity with the canonical element of D. melanogaster (X14037), which is the first schematic representation. Black rectangles - long terminal repeats. Gray rectangles - coding regions. Asterisk - genomic sequences interrupted by the scaffold ends or Ns. Striped rectangles - region used in the phylogenetic analyses. [file 1471-2148-9-279-S8.TIFF]
