# Supplementary material for: Multiple invasions of Gypsy and Micropia retroelements in genus Zaprionus and melanogaster subgroup of the genus Drosophila
Source: BMC Evol Biol. 2009 Dec 2;9:279. doi: 10.1186/1471-2148-9-279 (PMC2797524; doi:10.1186/1471-2148-9-279)
Supplement: Additional file 3 — Description of the genomic sequences from the 12 Drosophila genome searches. Chromosome and nucleotide location of the Gypsy and Micropia insertions from the Drosophila genomes. [file 1471-2148-9-279-S3.DOC]

**Additional file 3. Description of the genomic sequences of the *Gypsy* and *Micropia* retroelements from the 12 *Drosophila* genome searches.**

| Species | Symbol | Region | Position | Sequences used in the phylogenies |
| --- | --- | --- | --- | --- |
| *Gypsy* |  |  |  |  |
| *D. melanogaster* | A1 | 3RHet | 1969099 - 1974357 | 1969693 - 1970168 |
|  | A2 | 2RHet | 397182 - 403167 | 401863 - 402339 |
|  | A3 | 3LHet | 1598017 - 1605156 | 1599041 - 1599517 |
|  | A4 | U | 4044279 - 4046477 | 4045342 - 4045802 |
|  | A5 | 3L | 24456926 - 24464149 | 24462706 - 24463191 |
|  | A6 | 2R | 936722 - 943950 | 937680 - 938165 |
|  | A7 | X | 21541058 - 21543024 | 21541979 - 21542452 |
|  | B5 | 3LHet | 1511049 - 1521737 | 1520386 - 1520867 |
|  |  |  |  |  |
| *D. simulans* | A1 | chr3h_Mrandom_053 | 1 - 1113 | 512 - 983 |
|  | A2 | chrX_Mrandom_1054 | 502 - 1828 | 1181 - 1662 |
|  | A3 | chr2h_Mrandom_019 | 36818 - 39383 | 37683 - 38164 |
|  | A4 | chrX_Mrandom_1056 | 1 - 1062 | 464 - 945 |
|  | B2 | chr2h_Mrandom_019 | 1 - 4582 | 3424 - 3905 |
|  | B3 | chrX_Mrandom_012 | 1520 - 3713 | 2361 - 2840 |
|  | B4 | chrX_Mrandom_1055 | 3230 - 4825 | 4088 - 4569 |
|  | B6 | X | 16608932 - 16616905 | 16613272 - 16613752 |
|  | B7 | chrX_Mrandom_1057 | 706 - 3354 | 1701 - 2174 |
|  |  |  |  |  |
| *D. sechellia* | A1 | scaffold_6727 | 1 - 1802 | 553 - 1034 |
|  | A2 | scaffold_217 | 37661 - 42942 | 38501 - 38982 |
|  | A3 | scaffold_2010 | 2302 - 4273 | 2401 - 2882 |
|  | A4 | scaffold_157 | 54187 - 62931 | 60762 - 61242 |
|  | A5 | scaffold_2705 | 1 - 3051 | 2505 - 2985 |
|  | A6 | scaffold_5941 | 1 - 1936 | 390 - 835 |
|  | A7 | scaffold_3971 | 1 - 2395 | 1615 - 2096 |
|  | A8 | scaffold_1311 | 1 - 4864 | 3875 - 4356 |
|  | A9 | scaffold_2671 | 2597 - 4308 | 2966 - 3447 |
|  | A10 | scaffold_7315 | 1 - 1715 | 931 - 1411 |
|  | A11 | scaffold_753 | 1702 - 5512 | 1956 - 2436 |
|  | B11 | scaffold_267 | 14048 - 17332 | 15475 - 15952 |
|  | B12 | scaffold_1749 | 5 - 2615 | 1881 - 2361 |
|  | B14 | scaffold_865 | 1 - 6550 | 5141 - 5579 |
|  | B15 | scaffold_205 | 10373 - 14669 | 11155 - 11595 |
|  | B17 | scaffold_1410 | 309 - 1221 | 563 - 1043 |
|  | B18 | scaffold_3135 | 331 - 2766 | 436 - 917 |
|  | B19 | scaffold_905 | 1332 - 5080 | 4346 - 4826 |
|  |  |  |  |  |
| *D. yakuba* | A1 | v2_chrUn_1037 | 1 - 1361 | 262 - 747 |
|  | A2 | v2_chrUn_120 | 94276 - 96507 | 95206 - 95691 |
|  | A3 | v2_chrUn_002 | 139343 - 146491 | 145016 - 145501 |
|  | A4 | v2_chrUn_838 | 1 - 1441 | 395 - 880 |
|  | A5 | v2_chrX_random_058 | 72050 - 75265 | 72970 - 73455 |
|  | A6 | v2_chrUn_884 | 10253 - 16672 | 11187 - 11668 |
|  |  |  |  |  |
| *D. erecta* | A1 | scaffold_4929 | 24203241 - 24205331 | 24204122 - 24204592 |
|  | A2 | scaffold_4845 | 1720865 - 1724669 | 1721676 - 1722133 |
|  | A3 | scaffold_1379 | 3713 - 8945 | 4655 - 5140 |
|  | A4 | scaffold_1371 | 4262 - 8935 | 5196 - 5681 |
|  | A5 | scaffold_4690 | 18092571 - 18100562 | 18093512 - 18093997 |
|  | A6 | scaffold_3351 | 9448 - 15310 | 14812 - 15287 |
|  | A8 | scaffold_603 | 671 - 3457 | 1478 - 1954 |
|  | B1 | scaffold_4784 | 24495181 - 24500501 | 24499320 - 24499797 |
|  | B8 | scaffold_3414 | 4024­ - 12349 | 11250 - 11731 |
|  | B9 | scaffold_1907 | 20322 - 24525 | 20931 - 21360 |
|  | B10 | scaffold_3160 | 6829 - 7462 | 6841 - 7304 |
|  |  |  |  |  |
| *Micropia* |  |  |  |  |
| *D. melanogaster* | A1 | 3RHet | 1080745 - 1086226 | 1083774 - 1084145 |
|  | A3 | 3R | 3175534 - 3180956 | 3177510 - 3177881 |
|  |  |  |  |  |
| *D. simulans* | A1 | chr2h_Mrandom_027 | 4300 - 8964 | 6515 - 6886 |
|  | A2 | chrU_M_786 | 730 - 4270 | 2638 - 3009 |
|  | A3 | chrU_M_6079 | 5082 - 10439 | 7173 - 7536 |
|  | A4 | 2R | 395130 - 399969 | 396614 - 396982 |
|  | A5 | chr2h_Mrandom_009 | 173724 - 179132 | 176810 - 177181 |
|  | A6 | chrU_M_2288 | 164 - 5444 | 3144 - 3507 |
|  |  |  |  |  |
| *D. sechellia* | A1 | scaffold_540 | 1 - 2953 | 515 - 886 |
|  | A2 | scaffold_89 | 1 - 3742 | 156 - 527 |
|  | A5 | scaffold_1378 | 818 - 4697 | 3013 - 3384 |
|  | A7 | scaffold_935 | 1 - 4015 | 1478 - 1849 |
|  | A8 | scaffold_72 | 66411 - 71638 | 69278 - 69650 |
|  | A9 | scaffold_28 | 676092 - 679680 | 679168 - 679531 |
|  | A11 | scaffold_13 | 1012422 - 1017366 | 1014547 - 1014918 |
|  | A18 | scaffold_2140 | 2 - 1748 | 50 - 417 |
|  | A19 | scaffold_5109 | 1 - 2096 | 119 - 456 |
|  | A20 | scaffold_57 | 125910 - 131203 | 128729 - 129077 |
|  | B8 | scaffold_218 | 9744 - 15671 | 12547 - 12926 |
|  | B19 | scaffold_62 | 138796 - 144237 | 141878 - 142228 |
|  |  |  |  |  |
| *D. yakuba* | A1 | v2_chr2h_random_005 | 699043 - 704244 | 701807 - 702178 |
|  | A2 | v2_chrX_random_046 | 254554 - 260003 | 257548 - 256648 |
|  | A3 | v2_chrUn_975 | 12077 - 16547 | 14667 - 15015 |
|  | B3 | v2_chrUn_1883 | 3493 - 8952 | 5436 - 5787 |
