# Supplementary material for: Multiple invasions of Gypsy and Micropia retroelements in genus Zaprionus and melanogaster subgroup of the genus Drosophila
Source: BMC Evol Biol. 2009 Dec 2;9:279. doi: 10.1186/1471-2148-9-279 (PMC2797524; doi:10.1186/1471-2148-9-279)
Supplement: Additional file 5 — Pairwise genetic distance among Gypsy sequences of Zaprionus, melanogaster, D. willistoni and S. latisfasciaeformis. Distances calculated by the MCL method as implemented by MEGA 4.1. The sequences were clustered according the phylogenetic clades. [file 1471-2148-9-279-S5.DOC]

**Additional file 5. Pairwise genetic distance among *Gypsy* sequences of *Zaprionus*, *melanogaster*, *D. willistoni* and *S. latisfasciaeformis*.**

|  | 1 | 2 | 3 | 4 | 5 | 6 | 7 | 8 | 9 | 10 | 11 | 12 | 13 | 14 | 15 | 16 | 17 | 18 | 19 | 20 | 21 | 22 | 23 | 24 | 25 | 26 | 27 | 28 | 29 | 30 | 31 | 32 | 33 | 34 | 35 | 36 | 37 |
| --- | --- | --- | --- | --- | --- | --- | --- | --- | --- | --- | --- | --- | --- | --- | --- | --- | --- | --- | --- | --- | --- | --- | --- | --- | --- | --- | --- | --- | --- | --- | --- | --- | --- | --- | --- | --- | --- |
| 1. Dwil |  |  |  |  |  |  |  |  |  |  |  |  |  |  |  |  |  |  |  |  |  |  |  |  |  |  |  |  |  |  |  |  |  |  |  |  |  |
| 2. Dwil | .014 |  |  |  |  |  |  |  |  |  |  |  |  |  |  |  |  |  |  |  |  |  |  |  |  |  |  |  |  |  |  |  |  |  |  |  |  |
| 3. Dwil | .006 | .008 |  |  |  |  |  |  |  |  |  |  |  |  |  |  |  |  |  |  |  |  |  |  |  |  |  |  |  |  |  |  |  |  |  |  |  |
| 4. Dyak6 | 1.111 | 1.091 | 1.089 |  |  |  |  |  |  |  |  |  |  |  |  |  |  |  |  |  |  |  |  |  |  |  |  |  |  |  |  |  |  |  |  |  |  |
| 5. Zdav2 | 1.060 | 1.041 | 1.040 | .038 |  |  |  |  |  |  |  |  |  |  |  |  |  |  |  |  |  |  |  |  |  |  |  |  |  |  |  |  |  |  |  |  |  |
| 6. Zdav3 | 1.114 | 1.108 | 1.093 | .059 | .023 |  |  |  |  |  |  |  |  |  |  |  |  |  |  |  |  |  |  |  |  |  |  |  |  |  |  |  |  |  |  |  |  |
| 7. Zgab1 | 1.168 | 1.134 | 1.131 | .055 | .047 | .064 |  |  |  |  |  |  |  |  |  |  |  |  |  |  |  |  |  |  |  |  |  |  |  |  |  |  |  |  |  |  |  |
| 8. Zgab2 | 1.174 | 1.154 | 1.151 | .057 | .040 | .062 | .011 |  |  |  |  |  |  |  |  |  |  |  |  |  |  |  |  |  |  |  |  |  |  |  |  |  |  |  |  |  |  |
| 9. Zgab3 | 1.190 | 1.185 | 1.166 | .06 | .048 | .064 | .018 | .015 |  |  |  |  |  |  |  |  |  |  |  |  |  |  |  |  |  |  |  |  |  |  |  |  |  |  |  |  |  |
| 10. Zafr1 | 1.147 | 1.128 | 1.126 | .059 | .019 | .036 | .059 | .052 | .064 |  |  |  |  |  |  |  |  |  |  |  |  |  |  |  |  |  |  |  |  |  |  |  |  |  |  |  |  |
| 11. Zafr2 | 1.085 | 1.040 | 1.052 | .047 | .029 | .053 | .054 | .057 | .064 | .045 |  |  |  |  |  |  |  |  |  |  |  |  |  |  |  |  |  |  |  |  |  |  |  |  |  |  |  |
| 12. Zafr3 | 1.087 | 1.068 | 1.053 | .041 | .023 | .042 | .048 | .050 | .050 | .038 | .015 |  |  |  |  |  |  |  |  |  |  |  |  |  |  |  |  |  |  |  |  |  |  |  |  |  |  |
| 13. Zind1 | 1.089 | 1.073 | 1.055 | .047 | .027 | .047 | .050 | .052 | .060 | .038 | .040 | .029 |  |  |  |  |  |  |  |  |  |  |  |  |  |  |  |  |  |  |  |  |  |  |  |  |  |
| 14. Zind2 | 1.071 | 1.051 | 1.050 | .028 | .015 | .034 | .038 | .040 | .043 | .029 | .019 | .012 | .025 |  |  |  |  |  |  |  |  |  |  |  |  |  |  |  |  |  |  |  |  |  |  |  |  |
| 15. Zind3 | 1.078 | 1.040 | 1.045 | .043 | .029 | .053 | .045 | .047 | .059 | .043 | .032 | .029 | .036 | .021 |  |  |  |  |  |  |  |  |  |  |  |  |  |  |  |  |  |  |  |  |  |  |  |
| 16. DmelA7 | 1.120 | 1.107 | 1.098 | .032 | .017 | .035 | .044 | .044 | .047 | .037 | .028 | .019 | .028 | .008 | .028 |  |  |  |  |  |  |  |  |  |  |  |  |  |  |  |  |  |  |  |  |  |  |
| 17. DmelB5 | 1.070 | 1.050 | 1.049 | .034 | .021 | .041 | .043 | .046 | .048 | .041 | .030 | .023 | .032 | .010 | .030 | .011 |  |  |  |  |  |  |  |  |  |  |  |  |  |  |  |  |  |  |  |  |  |
| 18. DsimA2 | 1.112 | 1.092 | 1.090 | .061 | .056 | .075 | .067 | .069 | .072 | .075 | .061 | .054 | .068 | .045 | .061 | .050 | .052 |  |  |  |  |  |  |  |  |  |  |  |  |  |  |  |  |  |  |  |  |
| 19. DsimA3 | 1.128 | 1.109 | 1.106 | .045 | .036 | .056 | .055 | .057 | .060 | .056 | .045 | .039 | .047 | .025 | .041 | .030 | .032 | .041 |  |  |  |  |  |  |  |  |  |  |  |  |  |  |  |  |  |  |  |
| 20. DsimA4 | 1.080 | 1.060 | 1.059 | .038 | .025 | .045 | .050 | .053 | .055 | .045 | .038 | .032 | .041 | .019 | .034 | .021 | .025 | .043 | .023 |  |  |  |  |  |  |  |  |  |  |  |  |  |  |  |  |  |  |
| 21. DsimB2 | 1.046 | 1.026 | 1.025 | .065 | .056 | .077 | .077 | .079 | .082 | .077 | .061 | .054 | .066 | .045 | .061 | .048 | .052 | .034 | .041 | .041 |  |  |  |  |  |  |  |  |  |  |  |  |  |  |  |  |  |
| 22. DsimB3 | 1.060 | 1.040 | 1.039 | .043 | .032 | .052 | .053 | .055 | .058 | .052 | .043 | .036 | .045 | .023 | .038 | .028 | .030 | .048 | .028 | .019 | .048 |  |  |  |  |  |  |  |  |  |  |  |  |  |  |  |  |
| 23. DsimB4 | 1.110 | 1.097 | 1.095 | .047 | .034 | .052 | .060 | .062 | .065 | .052 | .043 | .036 | .043 | .023 | .043 | .026 | .025 | .052 | .032 | .025 | .052 | .030 |  |  |  |  |  |  |  |  |  |  |  |  |  |  |  |
| 24. DsimB6 | 1.106 | 1.092 | 1.090 | .049 | .040 | .058 | .062 | .064 | .067 | .058 | .049 | .043 | .049 | .030 | .045 | .035 | .032 | .045 | .034 | .028 | .050 | .032 | .023 |  |  |  |  |  |  |  |  |  |  |  |  |  |  |
| 25. DsimB7 | 1.087 | 1.067 | 1.066 | .064 | .064 | .083 | .076 | .078 | .081 | .083 | .064 | .062 | .076 | .053 | .069 | .056 | .057 | .017 | .048 | .050 | .039 | .053 | .057 | .057 |  |  |  |  |  |  |  |  |  |  |  |  |  |
| 26. DsecA1 | 1.075 | 1.055 | 1.054 | .047 | .038 | .059 | .060 | .062 | .065 | .059 | .043 | .036 | .050 | .028 | .043 | .032 | .034 | .021 | .028 | .030 | .017 | .034 | .039 | .036 | .028 |  |  |  |  |  |  |  |  |  |  |  |  |
| 27. DsecA2 | 1.083 | 1.063 | 1.062 | .052 | .045 | .065 | .067 | .070 | .072 | .065 | .049 | .043 | .056 | .034 | .049 | .039 | .041 | .023 | .034 | .036 | .028 | .041 | .045 | .043 | .030 | .010 |  |  |  |  |  |  |  |  |  |  |  |
| 28. DsecA3 | 1.100 | 1.080 | 1.078 | .050 | .041 | .059 | .060 | .062 | .065 | .059 | .045 | .038 | .052 | .030 | .045 | .035 | .036 | .032 | .028 | .030 | .030 | .034 | .039 | .039 | .039 | .017 | .023 |  |  |  |  |  |  |  |  |  |  |
| 29. DsecA4 | 1.104 | 1.089 | 1.087 | .054 | .045 | .063 | .065 | .067 | .070 | .066 | .050 | .043 | .057 | .034 | .050 | .039 | .039 | .028 | .034 | .036 | .025 | .041 | .041 | .039 | .035 | .010 | .017 | .028 |  |  |  |  |  |  |  |  |  |
| 30. DsecA5 | 1.131 | 1.117 | 1.114 | .041 | .032 | .052 | .053 | .055 | .058 | .052 | .036 | .030 | .043 | .021 | .036 | .026 | .028 | .039 | .032 | .026 | .034 | .030 | .034 | .034 | .046 | .017 | .028 | .017 | .028 |  |  |  |  |  |  |  |  |
| 31. DsecA6 | 1.215 | 1.203 | 1.190 | .049 | .042 | .061 | .063 | .063 | .063 | .059 | .046 | .037 | .054 | .030 | .046 | .037 | .039 | .025 | .035 | .037 | .023 | .042 | .047 | .044 | .033 | .004 | .011 | .016 | .016 | .021 |  |  |  |  |  |  |  |
| 32. DsecA7 | 1.113 | 1.093 | 1.091 | .038 | .030 | .050 | .050 | .053 | .055 | .050 | .034 | .028 | .041 | .019 | .034 | .024 | .025 | .034 | .023 | .017 | .034 | .021 | .025 | .025 | .041 | .021 | .028 | .021 | .028 | .017 | .028 |  |  |  |  |  |  |
| 33. DsecA8 | 1.100 | 1.080 | 1.078 | .038 | .030 | .050 | .050 | .053 | .055 | .050 | .038 | .032 | .041 | .019 | .034 | .024 | .025 | .039 | .019 | .017 | .039 | .021 | .025 | .028 | .044 | .025 | .032 | .025 | .032 | .026 | .032 | .017 |  |  |  |  |  |
| 34. DsecA9 | 1.116 | 1.096 | 1.094 | .045 | .036 | .057 | .058 | .060 | .063 | .057 | .045 | .039 | .048 | .026 | .041 | .030 | .032 | .028 | .026 | .028 | .028 | .032 | .037 | .034 | .035 | .013 | .019 | .023 | .019 | .026 | .016 | .023 | .023 |  |  |  |  |
| 35. DsecA10 | 1.119 | 1.104 | 1.102 | .043 | .034 | .052 | .055 | .058 | .060 | .054 | .039 | .032 | .045 | .023 | .039 | .028 | .030 | .039 | .032 | .026 | .034 | .030 | .034 | .034 | .046 | .017 | .028 | .021 | .028 | .004 | .023 | .017 | .026 | .030 |  |  |  |
| 36. DsecA11 | 1.123 | 1.109 | 1.106 | .047 | .038 | .056 | .062 | .065 | .067 | .056 | .047 | .041 | .047 | .028 | .047 | .032 | .030 | .057 | .034 | .030 | .057 | .034 | .021 | .023 | .064 | .043 | .050 | .043 | .045 | .039 | .052 | .030 | .030 | .041 | .039 |  |  |
| 37. DsecB11 | 1.130 | 1.116 | 1.113 | .046 | .037 | .055 | .058 | .061 | .063 | .057 | .041 | .034 | .048 | .026 | .041 | .030 | .032 | .041 | .035 | .028 | .037 | .032 | .037 | .037 | .046 | .019 | .030 | .024 | .030 | .011 | .023 | .019 | .028 | .032 | .011 | .041 |  |
| 38. DsecB12 | 1.116 | 1.102 | 1.099 | .054 | .041 | .059 | .065 | .067 | .070 | .059 | .050 | .043 | .050 | .030 | .050 | .035 | .032 | .059 | .037 | .032 | .059 | .037 | .023 | .025 | .067 | .045 | .052 | .045 | .043 | .041 | .054 | .032 | .032 | .043 | .041 | .015 | .044 |
| 39. DsecB14 | 1.253 | 1.240 | 1.226 | .065 | .065 | .085 | .088 | .088 | .089 | .083 | .070 | .060 | .078 | .052 | .067 | .060 | .062 | .038 | .053 | .055 | .040 | .060 | .065 | .062 | .046 | .026 | .023 | .038 | .038 | .043 | .030 | .045 | .050 | .035 | .045 | .070 | .045 |
| 40. DsecB15 | 1.197 | 1.170 | 1.189 | .049 | .037 | .059 | .061 | .063 | .072 | .054 | .044 | .040 | .040 | .028 | .040 | .033 | .030 | .057 | .038 | .030 | .057 | .035 | .021 | .023 | .063 | .042 | .050 | .045 | .045 | .040 | .051 | .030 | .030 | .040 | .040 | .011 | .040 |
| 41. DsecB17 | 1.119 | 1.104 | 1.102 | .059 | .045 | .063 | .070 | .072 | .075 | .063 | .054 | .047 | .054 | .034 | .054 | .039 | .036 | .064 | .041 | .037 | .061 | .039 | .028 | .030 | .072 | .050 | .057 | .050 | .048 | .046 | .059 | .037 | .037 | .048 | .046 | .015 | .048 |
| 42. DsecB18 | 1.124 | 1.110 | 1.108 | .056 | .043 | .061 | .067 | .069 | .072 | .061 | .051 | .045 | .052 | .032 | .052 | .037 | .034 | .059 | .039 | .034 | .059 | .039 | .025 | .027 | .067 | .045 | .052 | .047 | .048 | .043 | .051 | .034 | .034 | .043 | .043 | .013 | .041 |
| 43. DsecB19 | 1.119 | 1.104 | 1.102 | .061 | .047 | .063 | .067 | .070 | .072 | .063 | .056 | .050 | .057 | .036 | .056 | .041 | .039 | .063 | .043 | .039 | .064 | .043 | .030 | .032 | .071 | .052 | .059 | .048 | .050 | .048 | .062 | .039 | .039 | .050 | .048 | .017 | .050 |
| 44. Ztub1 | 1.136 | 1.119 | 1.115 | .231 | .21 | .225 | .243 | .240 | .250 | .222 | .226 | .218 | .225 | .212 | .229 | .221 | .223 | .225 | .232 | .231 | .235 | .238 | .238 | .242 | .238 | .217 | .223 | .223 | .224 | .224 | .220 | .225 | .225 | .227 | .227 | .230 | .232 |
| 45. DmelA1 | 1.271 | 1.239 | 1.247 | .236 | .227 | .237 | .256 | .260 | .268 | .240 | .241 | .233 | .240 | .224 | .244 | .229 | .233 | .236 | .242 | .241 | .237 | .242 | .248 | .253 | .246 | .227 | .233 | .233 | .228 | .234 | .228 | .236 | .230 | .237 | .237 | .243 | .240 |
| 46. DmelA2 | 1.279 | 1.246 | 1.254 | .236 | .227 | .237 | .260 | .260 | .268 | .240 | .238 | .233 | .240 | .225 | .244 | .229 | .233 | .236 | .242 | .242 | .237 | .249 | .248 | .253 | .244 | .228 | .233 | .233 | .229 | .235 | .231 | .236 | .230 | .237 | .238 | .244 | .240 |
| 47. DmelA3 | 1.249 | 1.231 | 1.225 | .246 | .236 | .247 | .267 | .271 | .278 | .249 | .251 | .242 | .250 | .234 | .254 | .238 | .242 | .248 | .252 | .251 | .249 | .258 | .258 | .263 | .256 | .237 | .243 | .243 | .244 | .244 | .238 | .245 | .239 | .247 | .247 | .253 | .249 |
| 48. DmelA4 | 1.406 | 1.397 | 1.395 | .256 | .246 | .257 | .272 | .279 | .291 | .256 | .258 | .249 | .254 | .243 | .258 | .248 | .252 | .255 | .255 | .261 | .259 | .266 | .268 | .270 | .263 | .246 | .252 | .252 | .253 | .253 | .249 | .255 | .249 | .256 | .256 | .259 | .259 |
| 49. DsimA1 | 1.275 | 1.258 | 1.251 | .237 | .228 | .238 | .255 | .258 | .262 | .241 | .242 | .234 | .241 | .225 | .245 | .227 | .231 | .234 | .240 | .239 | .225 | .250 | .240 | .249 | .244 | .225 | .231 | .231 | .229 | .232 | .222 | .233 | .231 | .235 | .235 | .242 | .237 |
| 50. DereA1 | 1.288 | 1.270 | 1.263 | .225 | .215 | .231 | .244 | .248 | .255 | .228 | .227 | .218 | .228 | .213 | .233 | .217 | .221 | .224 | .230 | .230 | .228 | .237 | .236 | .241 | .234 | .216 | .222 | .222 | .222 | .223 | .216 | .224 | .218 | .225 | .226 | .232 | .228 |
| 51. DereA2 | 1.370 | 1.360 | 1.351 | .251 | .238 | .252 | .263 | .267 | .268 | .248 | .250 | .241 | .255 | .232 | .253 | .238 | .241 | .244 | .251 | .250 | .248 | .258 | .257 | .262 | .249 | .235 | .241 | .241 | .242 | .242 | .235 | .244 | .238 | .245 | .245 | .252 | .239 |
| 52. Ztub2 | 1.170 | 1.130 | 1.148 | .126 | .119 | .138 | .157 | .154 | .163 | .132 | .112 | .114 | .134 | .110 | .122 | .123 | .123 | .135 | .133 | .128 | .130 | .128 | .133 | .138 | .132 | .115 | .125 | .128 | .126 | .123 | .118 | .125 | .130 | .123 | .123 | .138 | .127 |
| 53. Ztub3 | 1.164 | 1.152 | 1.142 | .128 | .121 | .136 | .160 | .157 | .160 | .135 | .119 | .112 | .132 | .112 | .129 | .123 | .125 | .138 | .136 | .130 | .133 | .131 | .136 | .140 | .135 | .118 | .128 | .130 | .128 | .126 | .118 | .128 | .133 | .126 | .126 | .141 | .129 |
| 54. Zcam1 | 1.114 | 1.101 | 1.092 | .106 | .111 | .125 | .137 | .140 | .143 | .129 | .111 | .109 | .119 | .100 | .114 | .107 | .108 | .120 | .118 | .110 | .125 | .115 | .113 | .117 | .117 | .105 | .115 | .120 | .115 | .111 | .112 | .110 | .110 | .110 | .108 | .118 | .111 |
| 55. Zcam2 | 1.100 | 1.099 | 1.091 | .115 | .111 | .130 | .147 | .139 | .148 | .128 | .121 | .113 | .123 | .109 | .118 | .114 | .114 | .129 | .127 | .119 | .134 | .125 | .122 | .124 | .126 | .114 | .124 | .124 | .122 | .117 | .119 | .119 | .119 | .120 | .115 | .125 | .118 |
| 56. Zcam3 | 1.163 | 1.151 | 1.141 | .126 | .119 | .139 | .160 | .152 | .160 | .137 | .132 | .124 | .135 | .119 | .129 | .123 | .125 | .143 | .139 | .128 | .146 | .133 | .133 | .138 | .141 | .125 | .136 | .136 | .136 | .129 | .131 | .131 | .131 | .131 | .126 | .139 | .132 |
| 57. Zdav1 | 1.090 | 1.063 | 1.069 | .098 | .099 | .118 | .130 | .127 | .135 | .117 | .104 | .102 | .112 | .093 | .102 | .100 | .100 | .113 | .111 | .103 | .118 | .108 | .106 | .110 | .110 | .098 | .108 | .108 | .108 | .103 | .104 | .103 | .103 | .103 | .098 | .111 | .104 |
| 58. DmelA5 | 1.112 | 1.099 | 1.090 | .101 | .106 | .120 | .127 | .129 | .132 | .124 | .111 | .104 | .114 | .095 | .109 | .102 | .103 | .115 | .113 | .105 | .120 | .110 | .108 | .112 | .112 | .100 | .110 | .115 | .110 | .106 | .107 | .105 | .105 | .106 | .103 | .113 | .106 |

**Additional file 5, continuation.**

|  | 1 | 2 | 3 | 4 | 5 | 6 | 7 | 8 | 9 | 10 | 11 | 12 | 13 | 14 | 15 | 16 | 17 | 18 | 19 | 20 | 21 | 22 | 23 | 24 | 25 | 26 | 27 | 28 | 29 | 30 | 31 | 32 | 33 | 34 | 35 | 36 | 37 |
| --- | --- | --- | --- | --- | --- | --- | --- | --- | --- | --- | --- | --- | --- | --- | --- | --- | --- | --- | --- | --- | --- | --- | --- | --- | --- | --- | --- | --- | --- | --- | --- | --- | --- | --- | --- | --- | --- |
| 59. DmelA6 | 1.105 | 1.092 | 1.084 | .103 | .109 | .123 | .129 | .132 | .135 | .126 | .114 | .107 | .116 | .097 | .111 | .104 | .105 | .108 | .106 | .103 | .113 | .108 | .106 | .110 | .105 | .098 | .108 | .108 | .108 | .108 | .104 | .103 | .103 | .101 | .105 | .111 | .109 |
| 60. DyakA1 | 1.096 | 1.083 | 1.075 | .094 | .099 | .113 | .122 | .124 | .127 | .112 | .099 | .092 | .107 | .083 | .099 | .095 | .096 | .108 | .106 | .098 | .112 | .103 | .101 | .105 | .110 | .093 | .103 | .108 | .103 | .098 | .096 | .098 | .098 | .098 | .096 | .106 | .099 |
| 61. DyakA2 | 1.120 | 1.107 | 1.098 | .101 | .107 | .121 | .127 | .129 | .133 | .119 | .107 | .100 | .114 | .090 | .107 | .102 | .103 | .115 | .113 | .105 | .120 | .111 | .108 | .113 | .117 | .101 | .110 | .115 | .111 | .106 | .104 | .105 | .106 | .106 | .103 | .113 | .107 |
| 62. DyakA3 | 1.108 | 1.095 | 1.086 | .096 | .102 | .116 | .124 | .127 | .130 | .114 | .102 | .095 | .109 | .086 | .102 | .097 | .098 | .110 | .108 | .101 | .115 | .106 | .103 | .108 | .112 | .096 | .105 | .110 | .106 | .101 | .099 | .101 | .101 | .101 | .098 | .108 | .102 |
| 63. DyakA4 | 1.108 | 1.095 | 1.086 | .096 | .102 | .116 | .124 | .127 | .130 | .114 | .102 | .095 | .109 | .086 | .102 | .097 | .098 | .110 | .108 | .101 | .115 | .106 | .103 | .108 | .112 | .096 | .105 | .110 | .106 | .101 | .099 | .101 | .101 | .101 | .098 | .108 | .102 |
| 64. DyakA5 | 1.090 | 1.077 | 1.069 | .101 | .106 | .120 | .129 | .132 | .135 | .119 | .106 | .099 | .114 | .090 | .106 | .102 | .103 | .115 | .113 | .105 | .120 | .110 | .103 | .112 | .114 | .100 | .110 | .115 | .110 | .106 | .104 | .105 | .105 | .105 | .103 | .113 | .106 |
| 65. DereA3 | 1.112 | 1.099 | 1.090 | .096 | .102 | .116 | .124 | .127 | .130 | .119 | .107 | .100 | .109 | .090 | .104 | .097 | .098 | .110 | .108 | .100 | .115 | .106 | .103 | .108 | .107 | .096 | .105 | .110 | .106 | .101 | .101 | .101 | .101 | .101 | .098 | .108 | .102 |
| 66. DereA4 | 1.124 | 1.112 | 1.102 | .098 | .104 | .118 | .127 | .129 | .127 | .122 | .109 | .102 | .112 | .093 | .107 | .100 | .101 | .113 | .111 | .103 | .117 | .108 | .106 | .110 | .110 | .098 | .108 | .113 | .108 | .103 | .101 | .103 | .103 | .103 | .101 | .111 | .104 |
| 67. DereA5 | 1.120 | 1.107 | 1.098 | .101 | .107 | .121 | .130 | .132 | .135 | .124 | .112 | .105 | .114 | .095 | .109 | .102 | .103 | .115 | .113 | .105 | .120 | .111 | .108 | .113 | .112 | .101 | .110 | .115 | .111 | .106 | .107 | .106 | .106 | .106 | .103 | .113 | .107 |
| 68. DereA6 | 1.164 | 1.144 | 1.141 | .093 | .092 | .099 | .125 | .125 | .128 | .107 | .109 | .100 | .105 | .088 | .102 | .093 | .095 | .107 | .105 | .092 | .114 | .100 | .100 | .104 | .106 | .097 | .102 | .107 | .098 | .098 | .099 | .097 | .097 | .102 | .095 | .105 | .098 |
| 69. DereA8 | 1.173 | 1.154 | 1.149 | .112 | .116 | .123 | .146 | .146 | .149 | .132 | .124 | .114 | .124 | .106 | .121 | .112 | .114 | .126 | .124 | .116 | .134 | .122 | .119 | .121 | .123 | .114 | .121 | .124 | .122 | .119 | .117 | .116 | .116 | .117 | .117 | .124 | .119 |
| 70. DereB1 | 1.239 | 1.233 | 1.236 | .112 | .121 | .128 | .152 | .154 | .157 | .137 | .129 | .121 | .132 | .112 | .126 | .118 | .119 | .126 | .129 | .119 | .134 | .124 | .124 | .129 | .126 | .114 | .122 | .129 | .122 | .119 | .118 | .121 | .122 | .122 | .117 | .129 | .120 |
| 71. DereB8 | 1.112 | 1.099 | 1.090 | .103 | .110 | .124 | .131 | .133 | .136 | .127 | .115 | .108 | .118 | .098 | .112 | .105 | .105 | .117 | .115 | .108 | .122 | .113 | .110 | .115 | .114 | .103 | .112 | .112 | .113 | .108 | .109 | .108 | .108 | .108 | .105 | .115 | .109 |
| 72. DereB9 | 1.354 | 1.334 | 1.325 | .084 | .084 | .100 | .122 | .122 | .123 | .092 | .100 | .089 | .095 | .076 | .092 | .082 | .087 | .100 | .095 | .079 | .097 | .090 | .090 | .095 | .099 | .084 | .092 | .092 | .092 | .082 | .089 | .087 | .087 | .090 | .082 | .095 | .082 |
| 73. DereB10 | 1.173 | 1.145 | 1.150 | .129 | .133 | .143 | .157 | .160 | .164 | .147 | .136 | .128 | .144 | .118 | .136 | .125 | .131 | .144 | .147 | .133 | .147 | .142 | .139 | .144 | .142 | .133 | .144 | .147 | .142 | .137 | .137 | .139 | .139 | .137 | .136 | .142 | .140 |
| 74. Slat1 | 1.180 | 1.140 | 1.144 | .117 | .106 | .133 | .140 | .139 | .150 | .124 | .114 | .111 | .121 | .106 | .116 | .114 | .115 | .129 | .127 | .119 | .124 | .125 | .127 | .129 | .124 | .105 | .110 | .107 | .117 | .105 | .101 | .112 | .120 | .117 | .107 | .127 | .108 |
| 75. Slat2 | 1.230 | 1.203 | 1.191 | .150 | .133 | .162 | .174 | .171 | .180 | .149 | .143 | .141 | .152 | .141 | .151 | .147 | .150 | .163 | .158 | .150 | .160 | .153 | .164 | .166 | .155 | .142 | .150 | .147 | .155 | .140 | .147 | .147 | .155 | .155 | .142 | .156 | .143 |
| 76. Slat3 | 1.204 | 1.171 | 1.164 | .129 | .117 | .138 | .152 | .149 | .158 | .136 | .125 | .120 | .131 | .120 | .130 | .126 | .129 | .139 | .134 | .134 | .136 | .134 | .143 | .145 | .130 | .115 | .126 | .123 | .129 | .118 | .120 | .126 | .134 | .129 | .121 | .135 | .121 |

**Additional file 5, continuation.**

|  | 38 | 39 | 40 | 41 | 42 | 43 | 44 | 45 | 46 | 47 | 48 | 49 | 50 | 51 | 52 | 53 | 54 | 55 | 56 | 57 | 58 | 59 | 60 | 61 | 62 | 63 | 64 | 65 | 66 | 67 | 68 | 69 | 70 | 71 | 72 | 73 | 74 | 75 |
| --- | --- | --- | --- | --- | --- | --- | --- | --- | --- | --- | --- | --- | --- | --- | --- | --- | --- | --- | --- | --- | --- | --- | --- | --- | --- | --- | --- | --- | --- | --- | --- | --- | --- | --- | --- | --- | --- | --- |
| 39. DsecB14 | .073 |  |  |  |  |  |  |  |  |  |  |  |  |  |  |  |  |  |  |  |  |  |  |  |  |  |  |  |  |  |  |  |  |  |  |  |  |  |
| 40. DsecB15 | .018 | .069 |  |  |  |  |  |  |  |  |  |  |  |  |  |  |  |  |  |  |  |  |  |  |  |  |  |  |  |  |  |  |  |  |  |  |  |  |
| 41. DsecB17 | .017 | .078 | .023 |  |  |  |  |  |  |  |  |  |  |  |  |  |  |  |  |  |  |  |  |  |  |  |  |  |  |  |  |  |  |  |  |  |  |  |
| 42. DsecB18 | .019 | .070 | .014 | .023 |  |  |  |  |  |  |  |  |  |  |  |  |  |  |  |  |  |  |  |  |  |  |  |  |  |  |  |  |  |  |  |  |  |  |
| 43. DsecB19 | .019 | .081 | .026 | .011 | .025 |  |  |  |  |  |  |  |  |  |  |  |  |  |  |  |  |  |  |  |  |  |  |  |  |  |  |  |  |  |  |  |  |  |
| 44. Ztub1 | .233 | .247 | .228 | .242 | .232 | .242 |  |  |  |  |  |  |  |  |  |  |  |  |  |  |  |  |  |  |  |  |  |  |  |  |  |  |  |  |  |  |  |  |
| 45. DmelA1 | .243 | .246 | .235 | .250 | .243 | .250 | .069 |  |  |  |  |  |  |  |  |  |  |  |  |  |  |  |  |  |  |  |  |  |  |  |  |  |  |  |  |  |  |  |
| 46. DmelA2 | .246 | .250 | .236 | .250 | .243 | .250 | .078 | .021 |  |  |  |  |  |  |  |  |  |  |  |  |  |  |  |  |  |  |  |  |  |  |  |  |  |  |  |  |  |  |
| 47. DmelA3 | .253 | .257 | .246 | .266 | .253 | .266 | .081 | .035 | .044 |  |  |  |  |  |  |  |  |  |  |  |  |  |  |  |  |  |  |  |  |  |  |  |  |  |  |  |  |  |
| 48. DmelA4 | .263 | .265 | .249 | .272 | .262 | .272 | .076 | .027 | .036 | .041 |  |  |  |  |  |  |  |  |  |  |  |  |  |  |  |  |  |  |  |  |  |  |  |  |  |  |  |  |
| 49. DsimA1 | .242 | .244 | .237 | .251 | .241 | .251 | .053 | .056 | .065 | .067 | .067 |  |  |  |  |  |  |  |  |  |  |  |  |  |  |  |  |  |  |  |  |  |  |  |  |  |  |  |
| 50. DereA1 | .232 | .237 | .226 | .244 | .231 | .244 | .065 | .026 | .035 | .033 | .034 | .056 |  |  |  |  |  |  |  |  |  |  |  |  |  |  |  |  |  |  |  |  |  |  |  |  |  |  |
| 51. DereA2 | .252 | .260 | .237 | .265 | .242 | .262 | .102 | .099 | .104 | .112 | .111 | .088 | .092 |  |  |  |  |  |  |  |  |  |  |  |  |  |  |  |  |  |  |  |  |  |  |  |  |  |
| 52. Ztub2 | .136 | .134 | .130 | .144 | .141 | .147 | .231 | .247 | .241 | .256 | .263 | .244 | .232 | .252 |  |  |  |  |  |  |  |  |  |  |  |  |  |  |  |  |  |  |  |  |  |  |  |  |
| 53. Ztub3 | .138 | .134 | .133 | .147 | .143 | .149 | .233 | .250 | .244 | .259 | .266 | .247 | .234 | .249 | .006 |  |  |  |  |  |  |  |  |  |  |  |  |  |  |  |  |  |  |  |  |  |  |  |
| 54. Zcam1 | .121 | .128 | .116 | .123 | .115 | .126 | .234 | .237 | .233 | .248 | .252 | .242 | .232 | .256 | .092 | .094 |  |  |  |  |  |  |  |  |  |  |  |  |  |  |  |  |  |  |  |  |  |  |
| 55. Zcam2 | .127 | .135 | .121 | .130 | .122 | .133 | .236 | .247 | .244 | .259 | .260 | .250 | .243 | .267 | .096 | .099 | .038 |  |  |  |  |  |  |  |  |  |  |  |  |  |  |  |  |  |  |  |  |  |
| 56. Zcam3 | .142 | .148 | .130 | .144 | .136 | .147 | .253 | .258 | .254 | .270 | .274 | .268 | .254 | .283 | .099 | .102 | .049 | .040 |  |  |  |  |  |  |  |  |  |  |  |  |  |  |  |  |  |  |  |  |
| 57. Zdav1 | .113 | .117 | .106 | .116 | .108 | .118 | .231 | .239 | .236 | .251 | .255 | .242 | .232 | .249 | .083 | .090 | .030 | .029 | .041 |  |  |  |  |  |  |  |  |  |  |  |  |  |  |  |  |  |  |  |
| 58. DmelA5 | .116 | .122 | .105 | .118 | .110 | .121 | .225 | .229 | .229 | .244 | .248 | .232 | .228 | .239 | .087 | .089 | .038 | .047 | .056 | .034 |  |  |  |  |  |  |  |  |  |  |  |  |  |  |  |  |  |  |
| 59. DmelA6 | .113 | .112 | .105 | .116 | .108 | .118 | .225 | .229 | .229 | .244 | .248 | .232 | .228 | .243 | .085 | .087 | .040 | .049 | .058 | .036 | .010 |  |  |  |  |  |  |  |  |  |  |  |  |  |  |  |  |  |
| 60. DyakA1 | .108 | .111 | .100 | .111 | .103 | .114 | .208 | .212 | .209 | .224 | .227 | .215 | .208 | .227 | .078 | .080 | .034 | .042 | .051 | .029 | .021 | .023 |  |  |  |  |  |  |  |  |  |  |  |  |  |  |  |  |
| 61. DyakA2 | .116 | .120 | .108 | .119 | .111 | .121 | .214 | .219 | .215 | .230 | .234 | .221 | .215 | .231 | .078 | .080 | .038 | .047 | .056 | .034 | .021 | .023 | .008 |  |  |  |  |  |  |  |  |  |  |  |  |  |  |  |
| 62. DyakA3 | .111 | .114 | .103 | .114 | .106 | .116 | .214 | .218 | .215 | .230 | .233 | .221 | .214 | .230 | .078 | .080 | .034 | .042 | .051 | .030 | .017 | .019 | .004 | .004 |  |  |  |  |  |  |  |  |  |  |  |  |  |  |
| 63. DyakA4 | .111 | .114 | .103 | .114 | .106 | .116 | .216 | .221 | .218 | .233 | .236 | .224 | .217 | .230 | .080 | .082 | .036 | .044 | .054 | .032 | .019 | .021 | .006 | .006 | .002 |  |  |  |  |  |  |  |  |  |  |  |  |  |
| 64. DyakA5 | .116 | .119 | .108 | .118 | .110 | .121 | .216 | .221 | .217 | .232 | .235 | .223 | .214 | .233 | .080 | .082 | .036 | .044 | .054 | .027 | .023 | .025 | .006 | .010 | .006 | .008 |  |  |  |  |  |  |  |  |  |  |  |  |
| 65. DereA3 | .111 | .117 | .103 | .114 | .106 | .116 | .219 | .223 | .224 | .238 | .242 | .227 | .223 | .240 | .082 | .085 | .032 | .040 | .047 | .027 | .012 | .015 | .017 | .021 | .017 | .019 | .019 |  |  |  |  |  |  |  |  |  |  |  |
| 66. DereA4 | .114 | .117 | .105 | .116 | .108 | .119 | .222 | .227 | .227 | .242 | .246 | .226 | .226 | .240 | .085 | .087 | .034 | .042 | .049 | .030 | .015 | .017 | .019 | .023 | .019 | .021 | .021 | .002 |  |  |  |  |  |  |  |  |  |  |
| 67. DereA5 | .116 | .123 | .108 | .119 | .111 | .121 | .225 | .230 | .230 | .245 | .249 | .233 | .229 | .246 | .087 | .090 | .036 | .045 | .052 | .032 | .017 | .019 | .021 | .025 | .021 | .023 | .023 | .004 | .006 |  |  |  |  |  |  |  |  |  |
| 68. DereA6 | .108 | .112 | .105 | .105 | .107 | .108 | .227 | .227 | .224 | .247 | .248 | .232 | .229 | .236 | .087 | .087 | .082 | .089 | .094 | .075 | .073 | .075 | .066 | .073 | .068 | .070 | .072 | .070 | .073 | .075 |  |  |  |  |  |  |  |  |
| 69. DereA8 | .127 | .133 | .120 | .130 | .122 | .132 | .237 | .233 | .236 | .252 | .249 | .240 | .236 | .257 | .111 | .111 | .046 | .048 | .059 | .037 | .050 | .052 | .041 | .050 | .046 | .048 | .048 | .043 | .046 | .048 | .066 |  |  |  |  |  |  |  |
| 70. DereB1 | .132 | .132 | .120 | .135 | .132 | .137 | .239 | .243 | .240 | .258 | .258 | .246 | .239 | .245 | .070 | .068 | .086 | .093 | .098 | .082 | .081 | .084 | .072 | .077 | .077 | .079 | .079 | .077 | .079 | .082 | .044 | .071 |  |  |  |  |  |  |
| 71. DereB8 | .118 | .125 | .113 | .121 | .113 | .121 | .242 | .245 | .241 | .257 | .261 | .248 | .241 | .256 | .093 | .095 | .030 | .034 | .045 | .021 | .034 | .036 | .030 | .034 | .030 | .032 | .032 | .028 | .030 | .032 | .080 | .037 | .086 |  |  |  |  |  |
| 72. DereB9 | .098 | .103 | .094 | .101 | .095 | .104 | .242 | .248 | .241 | .261 | .266 | .238 | .234 | .241 | .079 | .079 | .086 | .094 | .100 | .078 | .076 | .076 | .065 | .073 | .068 | .070 | .073 | .073 | .073 | .078 | .017 | .076 | .051 | .084 |  |  |  |  |
| 73. DereB10 | .151 | .149 | .131 | .151 | .145 | .150 | .267 | .262 | .259 | .275 | .276 | .262 | .255 | .272 | .070 | .068 | .092 | .101 | .107 | .087 | .087 | .089 | .075 | .077 | .077 | .079 | .079 | .082 | .084 | .087 | .088 | .107 | .068 | .089 | .090 |  |  |  |
| 74. Slat1 | .128 | .119 | .124 | .135 | .130 | .138 | .236 | .244 | .240 | .255 | .257 | .252 | .234 | .257 | .104 | .111 | .109 | .113 | .117 | .100 | .097 | .099 | .092 | .099 | .095 | .097 | .099 | .097 | .099 | .102 | .078 | .116 | .101 | .108 | .071 | .122 |  |  |
| 75. Slat2 | .159 | .159 | .161 | .168 | .161 | .168 | .256 | .262 | .255 | .272 | .263 | .264 | .258 | .273 | .146 | .149 | .144 | .149 | .155 | .139 | .138 | .141 | .130 | .138 | .133 | .135 | .138 | .136 | .138 | .141 | .111 | .155 | .144 | .142 | .106 | .166 | .079 |  |
| 76. Slat3 | .136 | .129 | .137 | .144 | .140 | .149 | .261 | .274 | .267 | .291 | .275 | .277 | .267 | .288 | .120 | .122 | .130 | .133 | .136 | .123 | .119 | .119 | .114 | .119 | .117 | .119 | .122 | .117 | .119 | .122 | .096 | .144 | .127 | .129 | .090 | .146 | .071 | .028 |

Symbols for species names: Dwil: *D. willistoni*; Ztub: *Z. tuberculatus*; Zcam: *Z. camerounensis*; Zdav: *Z. davidi*; Zgab: *Z. gabonicus*; Zafr: *Z. africanus*; Zind: *Z. indianus*; Dmel: *D. melanogaster*; Dsim: *D. simulans*; Dsec: *D. sechellia*; Dyak: *D. yakuba*; Dere: *D. erecta*.
