# Supplementary material for: Multiple invasions of Gypsy and Micropia retroelements in genus Zaprionus and melanogaster subgroup of the genus Drosophila
Source: BMC Evol Biol. 2009 Dec 2;9:279. doi: 10.1186/1471-2148-9-279 (PMC2797524; doi:10.1186/1471-2148-9-279)
Supplement: Additional file 6 — dN (below) and dS (above) values of pairwise comparisons among Zaprionus and melanogaster Gypsy sequences. Distances calculated by Nei-Gojobori method (Jukes-Cantor's correction), as implemented by MEGA 4.1. [file 1471-2148-9-279-S6.DOC]

**Additional file 6. dN (below) and dS (above) values of pairwise comparisons among *Zaprionus* and *melanogaster* *Gypsy* sequences.**

|  | 1 | 2 | 3 | 4 | 5 | 6 | 7 | 8 | 9 | 10 | 11 | 12 | 13 | 14 | 15 | 16 | 17 | 18 | 19 | 20 | 21 | 22 | 23 | 24 | 25 | 26 | 27 | 28 | 29 | 30 | 31 | 32 |
| --- | --- | --- | --- | --- | --- | --- | --- | --- | --- | --- | --- | --- | --- | --- | --- | --- | --- | --- | --- | --- | --- | --- | --- | --- | --- | --- | --- | --- | --- | --- | --- | --- |
| 1. DmelA7 | - | .014 | .152 | .080 | .057 | .151 | .043 | .028 | .072 | .152 | .103 | .118 | .119 | .087 | .072 | .073 | .088 | .058 | .072 | .042 | .072 | .043 | .028 | .042 | .028 | .043 | .028 | .087 | .028 | .072 | .088 | .057 |
| 2. DmelB5 | .000 | - | .136 | .065 | .042 | .135 | .028 | .028 | .057 | .136 | .087 | .102 | .103 | .072 | .057 | .057 | .072 | .043 | .057 | .028 | .057 | .028 | .014 | .028 | .014 | .028 | .014 | .072 | .014 | .057 | .073 | .042 |
| 3. DsimA2 | .013 | .013 | - | .081 | .119 | .088 | .105 | .136 | .104 | .058 | .072 | .087 | .058 | .057 | .103 | .104 | .120 | .073 | .103 | .136 | .103 | .137 | .120 | .136 | .120 | .171 | .154 | .223 | .152 | .170 | .226 | .151 |
| 4. DsimA3 | .015 | .015 | .020 | - | .050 | .080 | .036 | .065 | .066 | .081 | .065 | .080 | .050 | .050 | .096 | .066 | .050 | .007 | .096 | .065 | .096 | .066 | .050 | .065 | .050 | .097 | .081 | .145 | .080 | .096 | .147 | .111 |
| 5. DsimA4 | .009 | .009 | .013 | .015 | - | .119 | .014 | .042 | .043 | .119 | .103 | .118 | .088 | .087 | .072 | .043 | .057 | .043 | .072 | .042 | .072 | .043 | .028 | .042 | .028 | .072 | .058 | .118 | .057 | .072 | .120 | .087 |
| 6. DsimB2 | .009 | .009 | .013 | .015 | .009 | - | .104 | .144 | .144 | .088 | .042 | .087 | .028 | .057 | .072 | .104 | .120 | .073 | .072 | .143 | .072 | .145 | .127 | .143 | .127 | .170 | .153 | .204 | .151 | .168 | .225 | .151 |
| 7. DsimB3 | .018 | .018 | .022 | .024 | .018 | .018 | - | .028 | .029 | .105 | .088 | .104 | .073 | .073 | .058 | .029 | .043 | .029 | .058 | .028 | .058 | .029 | .014 | .028 | .014 | .058 | .043 | .104 | .043 | .058 | .105 | .072 |
| 8. DsimB4 | .018 | .018 | .022 | .024 | .018 | .015 | .026 | - | .058 | .136 | .119 | .135 | .104 | .103 | .087 | .058 | .073 | .058 | .087 | .028 | .087 | .028 | .014 | .028 | .014 | .058 | .043 | .087 | .042 | .087 | .088 | .072 |
| 9. DsimB6 | .013 | .013 | .018 | .020 | .013 | .011 | .022 | .013 | - | .137 | .119 | .135 | .104 | .103 | .088 | .058 | .073 | .058 | .088 | .057 | .088 | .058 | .043 | .057 | .043 | .088 | .073 | .119 | .072 | .088 | .121 | .103 |
| 10. DsimB7 | .018 | .018 | .009 | .024 | .018 | .018 | .026 | .026 | .022 | - | .072 | .087 | .058 | .057 | .103 | .104 | .101 | .073 | .103 | .136 | .103 | .137 | .120 | .136 | .120 | .171 | .154 | .223 | .152 | .170 | .226 | .151 |
| 11. DsecA1 | .004 | .004 | .009 | .011 | .004 | .004 | .013 | .013 | .009 | .013 | - | .042 | .014 | .014 | .028 | .088 | .103 | .043 | .028 | .119 | .028 | .120 | .103 | .119 | .103 | .119 | .104 | .168 | .103 | .118 | .170 | .102 |
| 12. DsecA2 | .009 | .009 | .013 | .015 | .009 | .009 | .018 | .018 | .013 | .018 | .004 | - | .057 | .028 | .072 | .103 | .119 | .058 | .072 | .135 | .072 | .136 | .118 | .135 | .118 | .135 | .120 | .185 | .118 | .134 | .187 | .117 |
| 13. DsecA3 | .013 | .013 | .018 | .020 | .013 | .013 | .022 | .022 | .018 | .022 | .009 | .004 | - | .028 | .043 | .073 | .088 | .043 | .043 | .104 | .043 | .104 | .088 | .104 | .088 | .137 | .121 | .187 | .119 | .136 | .189 | .119 |
| 14. DsecA4 | .009 | .009 | .013 | .015 | .009 | .009 | .018 | .018 | .013 | .018 | .004 | .009 | .013 | - | .042 | .073 | .088 | .028 | .042 | .103 | .042 | .104 | .087 | .103 | .087 | .103 | .088 | .151 | .087 | .103 | .153 | .087 |
| 15. DsecA5 | .004 | .004 | .009 | .011 | .004 | .004 | .013 | .013 | .009 | .013 | .000 | .004 | .009 | .004 | - | .057 | .103 | .073 | 0 | .087 | 0 | .088 | .072 | .087 | .072 | .088 | .073 | .135 | .072 | .087 | .136 | .071 |
| 16. DsecA7 | .009 | .009 | .013 | .015 | .009 | .009 | .017 | .018 | .013 | .018 | .004 | .009 | .013 | .009 | .004 | - | .073 | .058 | .057 | .058 | .057 | .058 | .043 | .058 | .043 | .089 | .073 | .136 | .073 | .088 | .138 | .072 |
| 17. DsecA8 | .009 | .009 | .013 | .015 | .009 | .009 | .017 | .018 | .013 | .018 | .004 | .009 | .013 | .009 | .004 | .009 | - | .043 | .103 | .073 | .103 | .073 | .057 | .073 | .057 | .104 | .089 | .152 | .088 | .103 | .154 | .119 |
| 18. DsecA9 | .013 | .013 | .017 | .020 | .013 | .013 | .022 | .022 | .017 | .022 | .009 | .013 | .017 | .013 | .009 | .013 | .013 | - | .073 | .058 | .073 | .058 | .043 | .058 | .043 | .073 | .058 | .120 | .058 | .073 | .122 | .088 |
| 19. DsecA10 | .004 | .004 | .009 | .011 | .004 | .004 | .013 | .013 | .009 | .013 | .000 | .004 | .009 | .004 | .000 | .004 | .004 | .009 | - | .087 | 0 | .088 | .072 | .087 | .072 | .088 | .073 | .135 | .072 | .087 | .136 | .071 |
| 20. DsecA11 | .022 | .022 | .026 | .024 | .022 | .020 | .031 | .022 | .009 | .031 | .018 | .022 | .026 | .022 | .018 | .022 | .022 | .026 | .018 | - | .087 | .028 | .014 | 0 | .014 | .057 | .043 | .087 | .042 | .087 | .088 | .072 |
| 21. DsecB11 | .009 | .009 | .013 | .015 | .009 | .009 | .018 | .018 | .013 | .018 | .004 | .009 | .013 | .009 | .004 | .009 | .009 | .013 | .004 | .022 | - | .088 | .072 | .087 | .072 | .088 | .073 | .135 | .072 | .087 | .136 | .071 |
| 22. DsecB12 | .031 | .031 | .035 | .033 | .031 | .029 | .040 | .031 | .018 | .040 | .026 | .031 | .035 | .022 | .026 | .031 | .031 | .035 | .026 | .018 | .031 | - | .014 | .028 | .014 | .058 | .043 | .088 | .043 | .088 | .089 | .072 |
| 23. DsecB17 | .031 | .031 | .036 | .033 | .031 | .029 | .040 | .031 | .018 | .040 | .027 | .031 | .036 | .031 | .027 | .031 | .031 | .035 | .027 | .009 | .031 | .018 | - | .014 | 0 | .043 | .028 | .072 | .028 | .072 | .073 | .057 |
| 24. DsecB18 | .022 | .022 | .026 | .024 | .022 | .020 | .031 | .022 | .009 | .031 | .018 | .022 | .026 | .022 | .018 | .022 | .022 | .026 | .018 | .009 | .022 | .018 | .018 | - | .014 | .057 | .043 | .087 | .042 | .087 | .096 | .072 |
| 25. DsecB19 | .027 | .027 | .031 | .029 | .027 | .024 | .035 | .027 | .013 | .036 | .022 | .027 | .031 | .027 | .022 | .026 | .026 | .031 | .022 | .004 | .027 | .013 | .004 | .013 | - | .043 | .028 | .072 | .028 | .072 | .073 | .057 |
| 26. Zdav2 | .004 | .004 | .018 | .020 | .013 | .013 | .022 | .022 | .018 | .022 | .009 | .013 | .018 | .013 | .009 | .013 | .013 | .017 | .009 | .026 | .013 | .035 | .036 | .026 | .031 | - | .014 | .103 | .043 | .057 | .043 | .042 |
| 27. Zdav3 | .018 | .018 | .031 | .033 | .026 | .026 | .035 | .035 | .031 | .035 | .022 | .026 | .031 | .026 | .022 | .026 | .026 | .031 | .022 | .040 | .026 | .049 | .049 | .040 | .045 | .013 | - | .088 | .028 | .073 | .058 | .057 |
| 28. Zind1 | .013 | .013 | .026 | .029 | .022 | .022 | .031 | .031 | .026 | .031 | .018 | .022 | .026 | .022 | .018 | .022 | .022 | .026 | .018 | .036 | .022 | .045 | .045 | .036 | .040 | .018 | .031 | - | .087 | .103 | .088 | .118 |
| 29. Zind2 | .000 | .000 | .013 | .015 | .009 | .009 | .018 | .018 | .013 | .018 | .004 | .009 | .013 | .009 | .004 | .009 | .009 | .013 | .004 | .022 | .009 | .031 | .031 | .022 | .027 | .004 | .018 | .013 | - | .057 | .058 | .028 |
| 30. Zind3 | .013 | .013 | .026 | .029 | .022 | .022 | .031 | .031 | .026 | .031 | .018 | .022 | .026 | .022 | .018 | .022 | .022 | .026 | .018 | .036 | .022 | .045 | .045 | .036 | .040 | .018 | .031 | .027 | .013 | - | .058 | .057 |
| 31. Zafr1 | .013 | .013 | .026 | .029 | .022 | .022 | .031 | .031 | .026 | .031 | .018 | .022 | .026 | .022 | .018 | .022 | .022 | .026 | .018 | .035 | .022 | .044 | .045 | .033 | .040 | .009 | .022 | .026 | .013 | .026 | - | .057 |
| 32. Zafr2 | .013 | .013 | .027 | .029 | .022 | .022 | .031 | .031 | .027 | .031 | .018 | .022 | .027 | .022 | .018 | .022 | .022 | .026 | .018 | .036 | .022 | .045 | .045 | .036 | .040 | .018 | .031 | .027 | .013 | .027 | .027 | - |
| 33. Zafr3 | .009 | .009 | .022 | .024 | .018 | .018 | .026 | .027 | .022 | .027 | .013 | .018 | .022 | .018 | .013 | .018 | .018 | .022 | .013 | .031 | .018 | .040 | .040 | .031 | .036 | .013 | .026 | .022 | .009 | .022 | .022 | .013 |
| 34. Zgab1 | .022 | .022 | .036 | .038 | .031 | .031 | .040 | .040 | .036 | .040 | .027 | .031 | .036 | .031 | .027 | .031 | .031 | .038 | .027 | .045 | .031 | .054 | .054 | .045 | .049 | .026 | .040 | .036 | .022 | .036 | .035 | .036 |
| 35. Zgab2 | .022 | .022 | .036 | .038 | .031 | .031 | .040 | .040 | .036 | .040 | .027 | .031 | .036 | .031 | .027 | .031 | .031 | .038 | .027 | .045 | .031 | .054 | .054 | .045 | .049 | .026 | .040 | .036 | .022 | .036 | .035 | .036 |
| 36. Zgab3 | .036 | .036 | .049 | .052 | .045 | .045 | .054 | .054 | .049 | .054 | .040 | .045 | .049 | .045 | .040 | .045 | .045 | .051 | .040 | .059 | .045 | .068 | .068 | .059 | .063 | .040 | .054 | .049 | .036 | .049 | .049 | .049 |
| 37. DmelA1 | .076 | .076 | .091 | .093 | .086 | .086 | .088 | .096 | .091 | .096 | .081 | .084 | .088 | .086 | .081 | .086 | .086 | .093 | .081 | .101 | .086 | .111 | .111 | .101 | .106 | .083 | .088 | .091 | .076 | .093 | .096 | .091 |
| 38. DmelA2 | .074 | .074 | .091 | .093 | .086 | .086 | .098 | .096 | .091 | .095 | .081 | .083 | .088 | .086 | .081 | .086 | .086 | .093 | .081 | .101 | .086 | .110 | .111 | .101 | .106 | .081 | .085 | .088 | .074 | .091 | .093 | .088 |
| 39. DmelA3 | .088 | .088 | .103 | .105 | .098 | .098 | .110 | .108 | .103 | .108 | .093 | .096 | .100 | .098 | .093 | .098 | .098 | .105 | .093 | .113 | .098 | .123 | .123 | .113 | .118 | .095 | .100 | .103 | .088 | .106 | .108 | .103 |
| 40. DsimA1 | .064 | .064 | .078 | .081 | .074 | .074 | .085 | .083 | .078 | .083 | .069 | .071 | .076 | .074 | .069 | .073 | .073 | .081 | .069 | .088 | .074 | .098 | .098 | .088 | .093 | .071 | .076 | .078 | .064 | .081 | .083 | .079 |
| 41. DereA1 | .064 | .064 | .078 | .081 | .074 | .074 | .085 | .083 | .078 | .083 | .069 | .071 | .076 | .074 | .069 | .073 | .073 | .081 | .069 | .088 | .074 | .098 | .098 | .088 | .093 | .071 | .085 | .078 | .064 | .081 | .083 | .079 |
| 42. Ztub1 | .078 | .078 | .093 | .095 | .088 | .088 | .100 | .098 | .093 | .098 | .083 | .086 | .090 | .088 | .083 | .088 | .088 | .095 | .083 | .098 | .088 | .113 | .108 | .103 | .103 | .085 | .090 | .093 | .078 | .096 | .098 | .093 |
| 43. DmelA5 | .009 | .009 | .013 | .024 | .018 | .018 | .027 | .027 | .022 | .018 | .013 | .018 | .022 | .018 | .013 | .018 | .018 | .022 | .013 | .031 | .018 | .040 | .040 | .031 | .036 | .015 | .029 | .022 | .009 | .024 | .024 | .013 |
| 44. DmelA6 | .009 | .009 | .013 | .024 | .018 | .018 | .027 | .027 | .022 | .018 | .013 | .018 | .022 | .018 | .013 | .018 | .018 | .022 | .013 | .031 | .018 | .040 | .040 | .031 | .036 | .015 | .029 | .022 | .009 | .024 | .024 | .013 |
| 45. DyakA1 | .013 | .013 | .018 | .029 | .022 | .022 | .031 | .031 | .027 | .022 | .018 | .022 | .027 | .022 | .018 | .022 | .022 | .027 | .018 | .036 | .022 | .045 | .045 | .036 | .040 | .020 | .033 | .027 | .013 | .029 | .029 | .018 |
| 46. DyakA2 | .013 | .013 | .018 | .029 | .022 | .022 | .031 | .031 | .027 | .022 | .018 | .022 | .027 | .022 | .018 | .022 | .022 | .027 | .018 | .036 | .022 | .045 | .045 | .036 | .040 | .020 | .033 | .027 | .013 | .029 | .029 | .018 |
| 47. DyakA3 | .013 | .013 | .018 | .029 | .022 | .022 | .031 | .031 | .027 | .022 | .018 | .022 | .027 | .022 | .018 | .022 | .022 | .027 | .018 | .036 | .022 | .045 | .045 | .036 | .040 | .020 | .033 | .027 | .013 | .029 | .029 | .018 |
| 48. DyakA4 | .013 | .013 | .018 | .029 | .022 | .022 | .031 | .031 | .027 | .022 | .018 | .022 | .027 | .022 | .018 | .022 | .022 | .027 | .018 | .036 | .022 | .045 | .045 | .036 | .040 | .020 | .033 | .027 | .013 | .029 | .029 | .018 |
| 49. DyakA5 | .013 | .013 | .018 | .029 | .022 | .022 | .031 | .031 | .027 | .022 | .018 | .022 | .027 | .022 | .018 | .022 | .022 | .027 | .018 | .036 | .022 | .045 | .045 | .036 | .040 | .020 | .033 | .027 | .013 | .029 | .029 | .018 |
| 50. DereA3 | .009 | .009 | .013 | .024 | .018 | .018 | .027 | .027 | .022 | .018 | .013 | .018 | .022 | .018 | .013 | .018 | .018 | .022 | .013 | .031 | .018 | .040 | .040 | .031 | .036 | .015 | .029 | .022 | .009 | .024 | .024 | .013 |
| 51. DereA4 | .013 | .013 | .018 | .029 | .022 | .022 | .031 | .031 | .027 | .022 | .018 | .022 | .027 | .022 | .018 | .022 | .022 | .027 | .018 | .036 | .022 | .045 | .045 | .036 | .040 | .020 | .033 | .027 | .013 | .029 | .029 | .018 |
| 52. DereA5 | .009 | .009 | .013 | .024 | .018 | .018 | .027 | .027 | .022 | .018 | .013 | .018 | .022 | .018 | .013 | .018 | .018 | .022 | .013 | .031 | .018 | .040 | .040 | .031 | .036 | .015 | .029 | .022 | .009 | .024 | .024 | .013 |
| 53. DereA8 | .036 | .036 | .040 | .052 | .045 | .045 | .054 | .054 | .049 | .045 | .040 | .045 | .040 | .045 | .040 | .045 | .045 | .049 | .040 | .059 | .045 | .068 | .068 | .059 | .064 | .042 | .047 | .050 | .036 | .052 | .052 | .040 |
| 54. DereB1 | .043 | .043 | .056 | .059 | .052 | .052 | .061 | .061 | .056 | .061 | .047 | .052 | .058 | .052 | .047 | .052 | .052 | .056 | .047 | .066 | .052 | .075 | .076 | .066 | .071 | .049 | .054 | .057 | .043 | .059 | .059 | .047 |
| 55. DereB8 | .031 | .031 | .036 | .047 | .040 | .040 | .049 | .050 | .045 | .040 | .036 | .040 | .036 | .040 | .036 | .040 | .040 | .045 | .036 | .054 | .040 | .064 | .064 | .054 | .059 | .038 | .052 | .045 | .031 | .047 | .047 | .036 |
| 56. DereB10 | .045 | .045 | .050 | .061 | .050 | .055 | .064 | .064 | .059 | .050 | .050 | .055 | .059 | .055 | .050 | .054 | .057 | .059 | .050 | .069 | .055 | .078 | .078 | .069 | .074 | .052 | .066 | .059 | .045 | .062 | .061 | .050 |
| 57. Ztub2 | .031 | .031 | .045 | .047 | .040 | .040 | .040 | .049 | .045 | .049 | .036 | .040 | .045 | .040 | .036 | .040 | .040 | .045 | .036 | .054 | .040 | .063 | .064 | .054 | .059 | .038 | .052 | .045 | .031 | .047 | .047 | .036 |
| 58. Ztub3 | .031 | .031 | .045 | .047 | .040 | .040 | .040 | .049 | .045 | .049 | .036 | .040 | .045 | .040 | .036 | .040 | .040 | .045 | .036 | .054 | .040 | .063 | .064 | .054 | .059 | .038 | .052 | .045 | .031 | .047 | .047 | .036 |
| 59. Zcam1 | .031 | .031 | .036 | .047 | .040 | .040 | .049 | .050 | .045 | .040 | .036 | .040 | .045 | .040 | .036 | .040 | .040 | .045 | .036 | .054 | .040 | .064 | .064 | .054 | .059 | .038 | .052 | .045 | .031 | .047 | .047 | .027 |
| 60. Zcam2 | .022 | .022 | .027 | .038 | .031 | .031 | .040 | .040 | .036 | .031 | .027 | .031 | .027 | .031 | .027 | .031 | .031 | .036 | .027 | .045 | .031 | .054 | .054 | .045 | .050 | .029 | .042 | .036 | .022 | .038 | .038 | .027 |
| 61. Zcam3 | .040 | .040 | .049 | .056 | .050 | .049 | .059 | .059 | .054 | .054 | .045 | .050 | .045 | .050 | .045 | .049 | .049 | .054 | .045 | .064 | .050 | .073 | .073 | .064 | .068 | .047 | .061 | .054 | .040 | .057 | .056 | .045 |
| 62. Zdav1 | .022 | .022 | .027 | .038 | .031 | .031 | .040 | .040 | .036 | .031 | .027 | .031 | .027 | .031 | .027 | .031 | .031 | .036 | .027 | .045 | .031 | .054 | .054 | .045 | .050 | .029 | .042 | .036 | .022 | .038 | .038 | .027 |

**Additional file 6, continuation.**

|  | 33 | 34 | 35 | 36 | 37 | 38 | 39 | 40 | 41 | 42 | 43 | 44 | 45 | 46 | 47 | 48 | 49 | 50 | 51 | 52 | 53 | 54 | 55 | 56 | 57 | 58 | 59 | 60 | 61 | 62 |
| --- | --- | --- | --- | --- | --- | --- | --- | --- | --- | --- | --- | --- | --- | --- | --- | --- | --- | --- | --- | --- | --- | --- | --- | --- | --- | --- | --- | --- | --- | --- |
| 1. DmelA7 | .042 | .087 | .103 | .072 | 1.466 | 1.362 | 1.384 | 1.367 | 1.454 | 1.231 | .425 | .477 | .399 | .422 | .399 | .399 | .399 | .425 | .425 | .425 | .453 | .491 | .380 | .418 | .507 | .507 | .422 | .450 | .429 | .381 |
| 2. DmelB5 | .028 | .072 | .087 | .057 | 1.378 | 1.284 | 1.304 | 1.367 | 1.367 | 1.165 | .401 | .452 | .376 | .399 | .376 | .376 | .376 | .401 | .401 | .401 | .429 | .465 | .358 | .442 | .481 | .481 | .399 | .426 | .406 | .359 |
| 3. DsimA2 | .135 | .152 | .170 | .136 | 1.181 | 1.078 | 1.184 | 1.171 | 1.171 | 1.009 | .560 | .508 | .528 | .556 | .528 | .528 | .528 | .560 | .560 | .560 | .595 | .522 | .506 | .551 | .540 | .540 | .556 | .591 | .567 | .535 |
| 4. DsimA3 | .096 | .112 | .128 | .096 | 1.470 | 1.324 | 1.387 | 1.458 | 1.458 | 1.231 | .495 | .446 | .466 | .491 | .466 | .466 | .466 | .495 | .495 | .495 | .527 | .569 | .445 | .540 | .529 | .529 | .491 | .523 | .500 | .472 |
| 5. DsimA4 | .072 | .087 | .103 | .072 | 1.566 | 1.405 | 1.475 | 1.554 | 1.554 | 1.304 | .449 | .452 | .422 | .446 | .422 | .422 | .422 | .449 | .449 | .449 | .479 | .517 | .403 | .492 | .535 | .535 | .446 | .475 | .454 | .427 |
| 6. DsimB2 | .135 | .186 | .204 | .169 | 1.303 | 1.184 | 1.234 | 1.292 | 1.292 | 1.107 | .555 | .504 | .524 | .552 | .524 | .524 | .524 | .555 | .555 | .555 | .621 | .604 | .502 | .604 | .535 | .535 | .552 | .586 | .562 | .531 |
| 7. DsimB3 | .058 | .073 | .089 | .058 | 1.480 | 1.332 | 1.396 | 1.468 | 1.468 | 1.238 | .433 | .435 | .406 | .430 | .406 | .406 | .406 | .433 | .433 | .433 | .462 | .500 | .387 | .475 | .517 | .517 | .430 | .459 | .437 | .411 |
| 8. DsimB4 | .057 | .103 | .119 | .088 | 1.481 | 1.335 | 1.398 | 1.300 | 1.470 | 1.242 | .427 | .429 | .401 | .424 | .401 | .401 | .401 | .427 | .427 | .427 | .455 | .493 | .381 | .444 | .510 | .510 | .424 | .452 | .431 | .383 |
| 9. DsimB6 | .087 | .103 | .120 | .088 | 1.603 | 1.433 | 1.507 | 1.590 | 1.590 | 1.328 | .453 | .456 | .426 | .450 | .426 | .426 | .426 | .453 | .453 | .453 | .457 | .522 | .406 | .497 | .540 | .540 | .450 | .480 | .458 | .431 |
| 10. DsimB7 | .135 | .152 | .170 | .136 | 1.181 | 1.078 | 1.121 | 1.171 | 1.171 | 1.009 | .505 | .456 | .475 | .501 | .475 | .475 | .475 | .505 | .505 | .505 | .537 | .522 | .455 | .551 | .540 | .540 | .501 | .534 | .510 | .482 |
| 11. DsecA1 | .087 | .135 | .152 | .119 | 1.227 | 1.119 | 1.165 | 1.217 | 1.217 | 1.047 | .474 | .477 | .446 | .471 | .446 | .446 | .446 | .474 | .474 | .474 | .532 | .517 | .426 | .518 | .455 | .455 | .471 | .501 | .479 | .452 |
| 12. DsecA2 | .102 | .151 | .168 | .135 | 1.255 | 1.143 | 1.191 | 1.245 | 1.245 | 1.070 | .525 | .528 | .495 | .522 | .495 | .495 | .495 | .525 | .525 | .525 | .558 | .543 | .474 | .572 | .506 | .506 | .522 | .554 | .531 | .501 |
| 13. DsecA3 | .103 | .152 | .170 | .136 | 1.362 | 1.234 | 1.289 | 1.351 | 1.351 | 1.152 | .505 | .456 | .475 | .501 | .475 | .475 | .475 | .505 | .505 | .505 | .566 | .543 | .455 | .551 | .485 | .485 | .501 | .534 | .510 | .482 |
| 14. DsecA4 | .072 | .118 | .135 | .103 | 1.102 | 1.009 | 1.047 | 1.093 | 1.093 | .946 | .500 | .503 | .471 | .497 | .471 | .471 | .471 | .500 | .500 | .500 | .532 | .517 | .450 | .545 | .481 | .481 | .497 | .528 | .506 | .477 |
| 15. DsecA5 | .057 | .103 | .119 | .087 | 1.299 | 1.181 | 1.231 | 1.288 | 1.288 | 1.104 | .425 | .477 | .399 | .422 | .399 | .399 | .399 | .425 | .425 | .425 | .479 | .465 | .380 | .467 | .455 | .455 | .422 | .450 | .429 | .404 |
| 16. DsecA7 | .057 | .104 | .120 | .088 | 1.508 | 1.356 | 1.421 | 1.496 | 1.496 | 1.260 | .480 | .483 | .452 | .477 | .452 | .452 | .452 | .480 | .480 | .480 | .511 | .552 | .431 | .525 | .514 | .514 | .477 | .508 | .485 | .457 |
| 17. DsecA8 | .103 | .119 | .136 | .104 | 1.405 | 1.270 | 1.328 | 1.393 | 1.393 | 1.328 | .505 | .508 | .475 | .501 | .475 | .475 | .475 | .505 | .505 | .505 | .537 | .579 | .455 | .537 | .599 | .599 | .501 | .534 | .510 | .482 |
| 18. DsecA9 | .073 | .081 | .096 | .065 | 1.308 | 1.187 | 1.238 | 1.297 | 1.297 | 1.108 | .457 | .435 | .430 | .455 | .430 | .430 | .430 | .457 | .457 | .457 | .488 | .528 | .410 | .501 | .490 | .490 | .455 | .485 | .463 | .435 |
| 19. DsecA10 | .057 | .103 | .119 | .087 | 1.299 | 1.181 | 1.231 | 1.288 | 1.288 | 1.104 | .425 | .477 | .399 | .422 | .399 | .399 | .399 | .425 | .425 | .425 | .479 | .465 | .380 | .467 | .455 | .455 | .422 | .450 | .429 | .404 |
| 20. DsecA11 | .057 | .103 | .119 | .087 | 1.307 | 1.187 | 1.238 | 1.296 | 1.296 | 1.109 | .426 | .429 | .400 | .424 | .400 | .400 | .400 | .426 | .426 | .426 | .455 | .492 | .381 | .420 | .509 | .509 | .424 | .452 | .431 | .382 |
| 21. DsecB11 | .057 | .103 | .119 | .087 | 1.299 | 1.181 | 1.231 | 1.288 | 1.288 | 1.104 | .425 | .477 | .399 | .422 | .399 | .399 | .399 | .425 | .425 | .425 | .479 | .465 | .380 | .467 | .455 | .455 | .422 | .450 | .429 | .404 |
| 22. DsecB12 | .057 | .104 | .120 | .088 | 1.255 | 1.207 | 1.190 | 1.245 | 1.245 | 1.068 | .430 | .433 | .404 | .427 | .404 | .404 | .404 | .430 | .430 | .430 | .459 | .497 | .384 | .473 | .461 | .461 | .427 | .456 | .435 | .385 |
| 23. DsecB17 | .042 | .087 | .103 | .072 | 1.382 | 1.252 | 1.308 | 1.371 | 1.371 | 1.168 | .402 | .404 | .377 | .400 | .377 | .377 | .377 | .402 | .402 | .402 | .429 | .466 | .358 | .443 | .482 | .482 | .400 | .427 | .406 | .359 |
| 24. DsecB18 | .057 | .103 | .119 | .087 | 1.307 | 1.187 | 1.238 | 1.296 | 1.296 | 1.109 | .426 | .429 | .400 | .424 | .400 | .400 | .400 | .426 | .426 | .426 | .455 | .492 | .381 | .420 | .509 | .509 | .424 | .452 | .431 | .382 |
| 25. DsecB19 | .042 | .087 | .103 | .072 | 1.387 | 1.255 | 1.312 | 1.375 | 1.375 | 1.171 | .403 | .405 | .378 | .400 | .378 | .378 | .378 | .403 | .403 | .403 | .430 | .466 | .359 | .444 | .482 | .482 | .400 | .427 | .407 | .360 |
| 26. Zdav2 | .057 | .103 | .088 | .088 | 1.449 | 1.347 | 1.369 | 1.438 | 1.438 | 1.152 | .441 | .495 | .414 | .438 | .414 | .414 | .414 | .441 | .441 | .441 | .470 | .509 | .395 | .484 | .472 | .526 | .438 | .418 | .398 | .351 |
| 27. Zdav3 | .043 | .088 | .104 | .073 | 1.470 | 1.364 | 1.387 | 1.458 | 1.458 | 1.231 | .419 | .472 | .393 | .417 | .393 | .393 | .393 | .419 | .419 | .419 | .448 | .485 | .374 | .461 | .502 | .502 | .417 | .445 | .424 | .375 |
| 28. Zind1 | .102 | .118 | .135 | .135 | 1.682 | 1.548 | 1.577 | 1.668 | 1.668 | 1.384 | .474 | .530 | .446 | .471 | .446 | .446 | .446 | .474 | .474 | .474 | .505 | .545 | .426 | .518 | .563 | .563 | .471 | .501 | .479 | .427 |
| 29. Zind2 | .014 | .087 | .103 | .072 | 1.466 | 1.362 | 1.384 | 1.454 | 1.454 | 1.231 | .425 | .477 | .355 | .376 | .355 | .355 | .355 | .425 | .425 | .425 | .453 | .491 | .380 | .418 | .455 | .455 | .422 | .450 | .429 | .381 |
| 30. Zind3 | .072 | .072 | .057 | .087 | 1.515 | 1.405 | 1.428 | 1.502 | 1.502 | 1.197 | .461 | .516 | .411 | .434 | .411 | .411 | .411 | .461 | .461 | .461 | .491 | .531 | .414 | .479 | .468 | .521 | .459 | .438 | .417 | .392 |
| 31. Zafr1 | .073 | .120 | .104 | .137 | 1.519 | 1.407 | 1.431 | 1.507 | 1.507 | 1.197 | .469 | .525 | .393 | .417 | .393 | .393 | .393 | .469 | .469 | .469 | .499 | .540 | .421 | .461 | .450 | .502 | .466 | .445 | .424 | .375 |
| 32. Zafr2 | .014 | .118 | .102 | .102 | 1.356 | 1.266 | 1.284 | 1.345 | 1.345 | 1.090 | .470 | .526 | .396 | .419 | .396 | .396 | .396 | .470 | .470 | .470 | .501 | .540 | .423 | .463 | .404 | .452 | .467 | .447 | .426 | .378 |
| 33. Zafr3 | - | .102 | .118 | .087 | 1.369 | 1.277 | 1.296 | 1.358 | 1.358 | 1.158 | .448 | .501 | .375 | .398 | .375 | .375 | .375 | .448 | .448 | .448 | .477 | .516 | .401 | .441 | .429 | .429 | .445 | .474 | .452 | .403 |
| 34. Zgab1 | .031 | - | .014 | .014 | 1.515 | 1.497 | 1.428 | 1.502 | 1.502 | 1.266 | .449 | .503 | .422 | .446 | .422 | .422 | .422 | .449 | .449 | .449 | .479 | .517 | .403 | .492 | .592 | .592 | .446 | .475 | .454 | .427 |
| 35. Zgab2 | .031 | .009 | - | .028 | 1.525 | 1.414 | 1.438 | 1.513 | 1.513 | 1.204 | .475 | .532 | .448 | .473 | .448 | .448 | .448 | .475 | .475 | .475 | .506 | .547 | .427 | .520 | .565 | .625 | .473 | .452 | .431 | .405 |
| 36. Zgab3 | .040 | .022 | .022 | - | 1.430 | 1.331 | 1.351 | 1.419 | 1.419 | 1.204 | .426 | .479 | .400 | .424 | .400 | .400 | .400 | .426 | .426 | .426 | .455 | .492 | .381 | .468 | .565 | .565 | .424 | .452 | .431 | .405 |
| 37. DmelA1 | .086 | .093 | .103 | .119 | - | .054 | .013 | .097 | .013 | .143 | 1.056 | 1.123 | .995 | .995 | 1.046 | 1.101 | .995 | 1.056 | 1.056 | 1.056 | 1.129 | .955 | .984 | .947 | .988 | 1.039 | .948 | 1.009 | 1.021 | 1.067 |
| 38. DmelA2 | .083 | .091 | .101 | .116 | .018 | - | .069 | .143 | .068 | .193 | 1.006 | 1.068 | .949 | .949 | .997 | 1.048 | .949 | 1.006 | 1.006 | 1.006 | 1.194 | .992 | .938 | .892 | .977 | 1.027 | .904 | .961 | .972 | 1.015 |
| 39. DmelA3 | .098 | .106 | .116 | .131 | .036 | .027 | - | .083 | .000 | .129 | 1.128 | 1.204 | 1.061 | 1.061 | 1.117 | 1.178 | 1.061 | 1.128 | 1.128 | 1.128 | 1.210 | 1.084 | 1.049 | 1.072 | 1.013 | 1.067 | 1.008 | 1.076 | 1.090 | 1.140 |
| 40. DsimA1 | .074 | .081 | .091 | .101 | .041 | .036 | .055 | - | .083 | .083 | 1.178 | 1.259 | 1.106 | 1.106 | 1.166 | 1.230 | 1.106 | 1.178 | 1.178 | 1.178 | 1.266 | 1.046 | 1.094 | .985 | .979 | 1.030 | 1.050 | 1.123 | 1.137 | 1.191 |
| 41. DereA1 | .074 | .081 | .091 | .106 | .032 | .022 | .041 | .041 | - | .129 | 1.178 | 1.259 | 1.106 | 1.106 | 1.166 | 1.230 | 1.106 | 1.178 | 1.178 | 1.178 | 1.266 | 1.046 | 1.094 | 1.036 | .979 | 1.030 | 1.050 | 1.123 | 1.137 | 1.191 |
| 42. Ztub1 | .088 | .096 | .105 | .121 | .050 | .045 | .064 | .041 | .050 | - | 1.128 | 1.204 | 1.061 | 1.061 | 1.117 | 1.178 | 1.061 | 1.128 | 1.128 | 1.128 | 1.210 | 1.004 | 1.049 | 1.020 | .894 | .988 | 1.008 | 1.022 | 1.035 | 1.082 |
| 43. DmelA5 | .009 | .031 | .031 | .045 | .088 | .084 | .098 | .074 | .074 | .089 | - | .056 | .055 | .055 | .041 | .055 | .055 | .055 | .055 | .055 | .148 | .281 | .070 | .230 | .334 | .334 | .115 | .131 | .101 | .100 |
| 44. DmelA6 | .009 | .031 | .031 | .045 | .088 | .084 | .098 | .074 | .074 | .088 | .000 | - | .085 | .085 | .070 | .085 | .085 | .085 | .085 | .085 | .182 | .323 | .100 | .268 | .336 | .336 | .147 | .164 | .133 | .132 |
| 45. DyakA1 | .013 | .036 | .036 | .050 | .088 | .079 | .094 | .074 | .069 | .089 | .004 | .004 | - | .027 | .013 | .027 | .000 | .055 | .055 | .055 | .115 | .242 | .041 | .160 | .253 | .253 | .084 | .099 | .070 | .070 |
| 46. DyakA2 | .013 | .036 | .036 | .050 | .088 | .079 | .094 | .074 | .069 | .089 | .004 | .004 | .000 | - | .013 | .027 | .027 | .084 | .084 | .084 | .147 | .242 | .070 | .160 | .253 | .253 | .114 | .130 | .100 | .100 |
| 47. DyakA3 | .013 | .036 | .036 | .050 | .088 | .079 | .094 | .074 | .069 | .089 | .004 | .004 | .000 | .000 | - | .013 | .013 | .069 | .069 | .069 | .131 | .261 | .055 | .177 | .272 | .272 | .099 | .115 | .085 | .085 |
| 48. DyakA4 | .013 | .036 | .036 | .050 | .088 | .079 | .094 | .074 | .069 | .089 | .004 | .004 | .000 | .000 | .000 | - | .027 | .084 | .084 | .084 | .147 | .280 | .070 | .194 | .291 | .291 | .114 | .130 | .100 | .100 |
| 49. DyakA5 | .013 | .036 | .036 | .050 | .088 | .079 | .094 | .074 | .069 | .089 | .004 | .004 | .000 | .000 | .000 | .000 | - | .055 | .055 | .055 | .115 | .242 | .041 | .160 | .253 | .253 | .084 | .099 | .070 | .070 |
| 50. DereA3 | .009 | .031 | .031 | .045 | .088 | .084 | .098 | .074 | .074 | .089 | .000 | .000 | .004 | .004 | .004 | .004 | .004 | - | .000 | .000 | .132 | .281 | .055 | .230 | .334 | .334 | .099 | .115 | .070 | .085 |
| 51. DereA4 | .013 | .036 | .036 | .040 | .093 | .089 | .103 | .074 | .079 | .093 | .004 | .004 | .009 | .009 | .009 | .009 | .009 | .004 | - | .000 | .132 | .281 | .055 | .230 | .334 | .334 | .099 | .115 | .070 | .085 |
| 52. DereA5 | .009 | .031 | .031 | .045 | .088 | .084 | .098 | .074 | .074 | .089 | .000 | .000 | .004 | .004 | .004 | .004 | .004 | .000 | .004 | - | .132 | .281 | .055 | .230 | .334 | .334 | .099 | .115 | .070 | .085 |
| 53. DereA8 | .036 | .059 | .059 | .073 | .097 | .088 | .103 | .079 | .079 | .098 | .027 | .027 | .022 | .022 | .022 | .022 | .022 | .027 | .031 | .027 | - | .204 | .070 | .307 | .427 | .427 | .115 | .132 | .117 | .101 |
| 54. DereB1 | .043 | .066 | .066 | .080 | .109 | .101 | .113 | .096 | .101 | .111 | .043 | .043 | .038 | .038 | .038 | .038 | .038 | .043 | .047 | .043 | .053 | - | .258 | .122 | .246 | .210 | .280 | .297 | .260 | .298 |
| 55. DereB8 | .031 | .054 | .054 | .068 | .110 | .101 | .116 | .096 | .091 | .111 | .022 | .022 | .018 | .018 | .018 | .018 | .018 | .022 | .027 | .022 | .031 | .058 | - | .212 | .314 | .314 | .041 | .056 | .042 | .027 |
| 56. DereB10 | .045 | .069 | .069 | .083 | .122 | .114 | .127 | .109 | .104 | .122 | .036 | .036 | .032 | .032 | .032 | .032 | .032 | .036 | .041 | .036 | .055 | .052 | .050 | - | .147 | .115 | .228 | .248 | .214 | .249 |
| 57. Ztub2 | .031 | .054 | .054 | .068 | .101 | .093 | .106 | .088 | .083 | .103 | .031 | .031 | .027 | .027 | .027 | .027 | .027 | .031 | .036 | .031 | .050 | .038 | .045 | .041 | - | .028 | .332 | .314 | .276 | .315 |
| 58. Ztub3 | .031 | .054 | .054 | .068 | .101 | .093 | .106 | .088 | .083 | .103 | .031 | .031 | .027 | .027 | .027 | .027 | .027 | .031 | .036 | .031 | .050 | .038 | .045 | .041 | .000 | - | .332 | .356 | .316 | .357 |
| 59. Zcam1 | .031 | .054 | .054 | .069 | .108 | .099 | .114 | .094 | .089 | .109 | .022 | .022 | .018 | .018 | .018 | .018 | .018 | .022 | .027 | .022 | .041 | .057 | .036 | .050 | .045 | .045 | - | .070 | .056 | .070 |
| 60. Zcam2 | .022 | .045 | .045 | .059 | .098 | .089 | .103 | .084 | .079 | .098 | .013 | .013 | .009 | .009 | .009 | .009 | .009 | .013 | .018 | .013 | .022 | .049 | .018 | .041 | .036 | .036 | .027 | - | .042 | .056 |
| 61. Zcam3 | .040 | .064 | .064 | .078 | .118 | .108 | .124 | .103 | .098 | .118 | .036 | .036 | .031 | .031 | .031 | .031 | .031 | .036 | .040 | .036 | .045 | .067 | .040 | .064 | .054 | .054 | .050 | .031 | - | .042 |
| 62. Zdav1 | .022 | .045 | .045 | .059 | .097 | .089 | .103 | .084 | .079 | .098 | .013 | .013 | .009 | .009 | .009 | .009 | .009 | .013 | .018 | .013 | .022 | .049 | .018 | .041 | .036 | .036 | .027 | .009 | .031 | - |

Symbols for species names: Ztub: *Z. tuberculatus*; Zcam: *Z. camerounensis*; Zdav: *Z. davidi*; Zgab: *Z. gabonicus*; Zafr: *Z. africanus*; Zind: *Z. indianus*; Dmel: *D. melanogaster*; Dsim: *D. simulans*; Dsec: *D. sechellia*; Dyak: *D. yakuba*; Dere: *D. erecta*.
