# Supplementary material for: Multiple invasions of Gypsy and Micropia retroelements in genus Zaprionus and melanogaster subgroup of the genus Drosophila
Source: BMC Evol Biol. 2009 Dec 2;9:279. doi: 10.1186/1471-2148-9-279 (PMC2797524; doi:10.1186/1471-2148-9-279)
Supplement: Additional file 7 — Z values (above) and significance P-values (below) for the Codon-Based Z-test of neutrality between Gypsy sequences of Zaprionus and melanogaster species. Test performed using alternative hypothesis of non-neutrality (dN ≠ dS) and Nei-Gojobori distance (Jukes-Cantor's correction). Gray cells correspond to significant (p < 0.05) pairwise comparisons. [file 1471-2148-9-279-S7.DOC]

**Additional file 7. Z values (above) and significance P-values (below) for the Codon Based Z-test of neutrality between *Gypsy* sequences of *Zaprionus* and *melanogaster* species.**

|  | 1 | 2 | 3 | 4 | 5 | 6 | 7 | 8 | 9 | 10 | 11 | 12 | 13 | 14 | 15 | 16 | 17 | 18 | 19 | 20 | 21 | 22 | 23 | 24 | 25 | 26 | 27 | 28 | 29 | 30 | 31 | 32 |
| --- | --- | --- | --- | --- | --- | --- | --- | --- | --- | --- | --- | --- | --- | --- | --- | --- | --- | --- | --- | --- | --- | --- | --- | --- | --- | --- | --- | --- | --- | --- | --- | --- |
| 1. DmelA7 | - | -.998 | -2.775 | -1.821 | -1.638 | -2.872 | -.959 | -.479 | -1.760 | -2.676 | -2.471 | -2.536 | -2.425 | -2.138 | -2.055 | -1.908 | -2.140 | -1.481 | -2.055 | -.765 | -1.905 | -.426 | .135 | -.765 | -.064 | -1.520 | -.489 | -2.003 | -1.407 | -1.756 | -2.009 | -1.467 |
| 2. DmelB5 | .320 | - | -2.607 | -1.550 | -1.322 | -2.711 | -.491 | -.479 | -1.476 | -2.501 | -2.274 | -2.346 | -2.226 | -1.905 | -1.808 | -1.643 | -1.908 | -1.142 | -1.808 | -.267 | -1.638 | .121 | .945 | -.267 | .718 | -1.162 | .214 | -1.756 | -.998 | -1.471 | -1.763 | -1.126 |
| 3. DsimA2 | .006 | .010 | - | -1.690 | -2.425 | -2.007 | -1.995 | -2.399 | -2.109 | -1.645 | -1.908 | -2.005 | -1.320 | -1.476 | -2.349 | -2.231 | -2.428 | -1.626 | -2.349 | -2.293 | -2.226 | -2.094 | -1.866 | -2.293 | -1.978 | -2.841 | -2.392 | -3.105 | -2.775 | -2.652 | -3.112 | -2.474 |
| 4. DsimA3 | .071 | .124 | .094 | - | -1.232 | -1.822 | -.473 | -1.253 | -1.406 | -1.555 | -1.704 | -1.819 | -1.070 | -1.232 | -2.185 | -1.557 | -1.238 | .923 | -2.185 | -1.252 | -2.059 | -.970 | -.572 | -1.252 | -.735 | -1.939 | -1.296 | -2.341 | -1.821 | -1.684 | -2.351 | -1.919 |
| 5. DsimA4 | .104 | .189 | .017 | .220 | - | -2.537 | .211 | -.948 | -1.137 | -2.312 | -2.471 | -2.536 | -2.007 | -2.138 | -2.055 | -1.327 | -1.641 | -1.142 | -2.055 | -.765 | -1.905 | -.426 | .135 | -.765 | -.064 | -1.760 | -1.003 | -2.194 | -1.638 | -1.463 | -2.203 | -1.731 |
| 6. DsimB2 | .005 | .008 | .047 | .071 | .012 | - | -2.110 | -2.640 | -2.744 | -1.875 | -1.519 | -2.137 | -.702 | -1.639 | -2.055 | -2.350 | -2.539 | -1.765 | -2.055 | -2.537 | -1.905 | -2.340 | -2.133 | -2.537 | -2.243 | -2.931 | -2.484 | -3.044 | -2.872 | -2.742 | -3.186 | -2.570 |
| 7. DsimB3 | .340 | .624 | .048 | .637 | .833 | .037 | - | -.087 | -.293 | -1.877 | -2.011 | -2.107 | -1.485 | -1.620 | -1.481 | -.502 | -.966 | -.302 | -1.481 | .109 | -1.320 | .465 | 1.335 | .109 | 1.131 | -1.170 | -.286 | -1.751 | -.959 | -.852 | -1.765 | -1.189 |
| 8. DsimB4 | .633 | .633 | .018 | .213 | .345 | .009 | .931 | - | -1.479 | -2.295 | -2.423 | -2.498 | -1.986 | -2.102 | -2.005 | -1.319 | -1.617 | -1.166 | -2.005 | -.272 | -1.872 | .115 | .939 | -.272 | .713 | -1.158 | -.267 | -1.478 | -.948 | -1.478 | -1.493 | -1.174 |
| 9. DsimB6 | .081 | .142 | .037 | .162 | .258 | .007 | .770 | .142 | - | -2.401 | -2.538 | -2.606 | -2.109 | -2.226 | -2.140 | -1.483 | -1.764 | -1.327 | -2.140 | -1.643 | -2.007 | -1.322 | -.953 | -1.643 | -1.139 | -1.879 | -1.203 | -2.087 | -1.760 | -1.612 | -2.098 | -1.857 |
| 10. DsimB7 | .008 | .014 | .103 | .123 | .022 | .063 | .063 | .023 | .018 | - | -1.760 | -1.872 | -1.162 | -1.314 | -2.226 | -2.111 | -2.052 | -1.485 | -2.226 | -2.189 | -2.104 | -1.991 | -1.755 | -2.189 | -1.867 | -2.749 | -2.295 | -3.026 | -2.676 | -2.560 | -3.034 | -2.375 |
| 11. DsecA1 | .015 | .025 | .059 | .091 | .015 | .131 | .047 | .017 | .012 | .081 | - | -1.518 | -.340 | -.651 | -1.407 | -2.276 | -2.472 | -1.330 | -1.407 | -2.309 | -1.160 | -2.089 | -1.857 | -2.309 | -1.979 | -2.538 | -1.987 | -2.835 | -2.471 | -2.307 | -2.840 | -2.095 |
| 12. DsecA2 | .013 | .021 | .047 | .071 | .013 | .035 | .037 | .014 | .010 | .064 | .132 | - | -1.809 | -.921 | -2.055 | -2.349 | -2.538 | -1.480 | -2.055 | -2.391 | -1.904 | -2.187 | -1.967 | -2.391 | -2.081 | -2.606 | -2.089 | -2.896 | -2.536 | -2.389 | -2.902 | -2.187 |
| 13. DsecA3 | .017 | .028 | .189 | .287 | .047 | .484 | .140 | .049 | .037 | .248 | .734 | .073 | - | -.701 | -1.326 | -1.766 | -2.011 | -.966 | -1.326 | -1.865 | -1.137 | -1.636 | -1.356 | -1.865 | -1.486 | -2.505 | -1.988 | -2.814 | -2.425 | -2.291 | -2.821 | -2.082 |
| 14. DsecA4 | .035 | .059 | .142 | .220 | .035 | .104 | .108 | .038 | .028 | .191 | .516 | .359 | .485 | - | -1.518 | -1.908 | -2.140 | -.707 | -1.518 | -1.979 | -1.322 | -1.985 | -1.475 | -1.979 | -1.607 | -2.226 | -1.617 | -2.573 | -2.138 | -1.977 | -2.580 | -1.731 |
| 15. DsecA5 | .042 | .073 | .020 | .031 | .042 | .042 | .141 | .047 | .034 | .028 | .162 | .042 | .187 | .132 | - | -1.810 | -2.472 | -1.910 | .000 | -1.871 | .999 | -1.615 | -1.321 | -1.871 | -1.466 | -2.140 | -1.475 | -2.497 | -2.055 | -1.869 | -2.504 | -1.604 |
| 16. DsecA7 | .059 | .103 | .028 | .122 | .187 | .020 | .616 | .190 | .141 | .037 | .025 | .020 | .080 | .059 | .073 | - | -1.911 | -1.488 | -1.810 | -1.159 | -1.643 | -.859 | -.427 | -1.159 | -.601 | -2.012 | -1.345 | -2.398 | -1.908 | -1.745 | -2.406 | -1.467 |
| 17. DsecA8 | .034 | .059 | .017 | .218 | .103 | .012 | .336 | .108 | .080 | .042 | .015 | .012 | .047 | .034 | .015 | .058 | - | -1.148 | -2.472 | -1.473 | -2.349 | -1.200 | -.844 | -1.473 | -1.000 | -2.230 | -1.625 | -2.577 | -2.140 | -1.983 | -2.585 | -2.195 |
| 18. DsecA9 | .141 | .256 | .107 | .358 | .256 | .080 | .763 | .246 | .187 | .140 | .186 | .142 | .336 | .481 | .058 | .139 | .253 | - | -1.910 | -1.009 | -1.764 | -.716 | -.267 | -1.009 | -.437 | -1.626 | -.869 | -2.094 | -1.481 | -1.336 | -2.105 | -1.613 |
| 19. DsecA10 | .042 | .073 | .020 | .031 | .042 | .042 | .141 | .047 | .034 | .028 | .162 | .042 | .187 | .132 | 1.000 | .073 | .015 | .058 | - | -1.871 | .999 | -1.615 | -1.321 | -1.871 | -1.466 | -2.140 | -1.475 | -2.497 | -2.055 | -1.869 | -2.504 | -1.604 |
| 20. DsecA11 | .446 | .790 | .024 | .213 | .446 | .012 | .914 | .786 | .103 | .031 | .023 | .018 | .065 | .050 | .064 | .249 | .143 | .315 | .064 | - | -1.738 | -.489 | -.337 | 1.412 | -.654 | -1.000 | -.101 | -1.348 | -.765 | -1.348 | -1.365 | -1.032 |
| 21. DsecB11 | .059 | .104 | .028 | .042 | .059 | .059 | .189 | .064 | .047 | .037 | .248 | .059 | .258 | .189 | .320 | .103 | .020 | .080 | .320 | .085 | - | -1.486 | -1.179 | -1.738 | -1.322 | -2.007 | -1.334 | -2.391 | -1.905 | -1.736 | -2.400 | -1.457 |
| 22. DsecB12 | .671 | .904 | .038 | .334 | .671 | .021 | .643 | .909 | .189 | .049 | .039 | .031 | .104 | .049 | .109 | .392 | .233 | .475 | .109 | .626 | .140 | - | .216 | -.489 | -.055 | -.705 | .202 | -1.105 | -.426 | -1.105 | -1.125 | -.767 |
| 23. DsecB17 | .893 | .346 | .065 | .568 | .893 | .035 | .184 | .349 | .343 | .082 | .066 | .052 | .178 | .143 | .189 | .670 | .401 | .790 | .189 | .736 | .241 | .830 | - | .225 | .999 | -.255 | .836 | -.763 | .135 | -.763 | -.785 | -.368 |
| 24. DsecB18 | .446 | .790 | .024 | .213 | .446 | .012 | .914 | .786 | .103 | .031 | .023 | .018 | .065 | .050 | .064 | .249 | .143 | .315 | .064 | .160 | .085 | .626 | .822 | - | -.047 | -1.000 | -.101 | -1.348 | -.765 | -1.348 | -1.564 | -1.032 |
| 25. DsecB19 | .949 | .474 | .050 | .464 | .949 | .027 | .260 | .477 | .257 | .064 | .050 | .040 | .140 | .111 | .145 | .549 | .319 | .663 | .145 | .515 | .189 | .956 | .320 | .962 | - | -.426 | .661 | -.902 | -.064 | -.902 | -.922 | -.520 |
| 26. Zdav2 | .131 | .247 | .005 | .055 | .081 | .004 | .244 | .249 | .063 | .007 | .012 | .010 | .014 | .028 | .034 | .046 | .028 | .107 | .034 | .319 | .047 | .482 | .799 | .319 | .671 | - | -.063 | -2.104 | -1.520 | -1.314 | -1.332 | -.946 |
| 27. Zdav3 | .626 | .831 | .018 | .197 | .318 | .014 | .775 | .790 | .231 | .023 | .049 | .039 | .049 | .108 | .143 | .181 | .107 | .386 | .143 | .920 | .185 | .840 | .405 | .920 | .510 | .950 | - | -1.489 | -.489 | -1.194 | -1.172 | -.840 |
| 28. Zind1 | .047 | .082 | .002 | .021 | .030 | .003 | .082 | .142 | .039 | .003 | .005 | .004 | .006 | .011 | .014 | .018 | .011 | .038 | .014 | .180 | .018 | .271 | .447 | .180 | .369 | .037 | .139 | - | -2.003 | -1.855 | -1.617 | -2.075 |
| 29. Zind2 | .162 | .320 | .006 | .071 | .104 | .005 | .340 | .345 | .081 | .008 | .015 | .013 | .017 | .035 | .042 | .059 | .034 | .141 | .042 | .446 | .059 | .671 | .893 | .446 | .949 | .131 | .626 | .047 | - | -1.471 | -1.480 | -.689 |
| 30. Zind3 | .082 | .144 | .009 | .095 | .146 | .007 | .396 | .142 | .110 | .012 | .023 | .018 | .024 | .050 | .064 | .084 | .050 | .184 | .064 | .180 | .085 | .271 | .447 | .180 | .369 | .191 | .235 | .066 | .144 | - | -1.003 | -.980 |
| 31. Zafr1 | .047 | .080 | .002 | .020 | .030 | .002 | .080 | .138 | .038 | .003 | .005 | .004 | .006 | .011 | .014 | .018 | .011 | .037 | .014 | .175 | .018 | .263 | .434 | .120 | .358 | .185 | .243 | .108 | .142 | .318 | - | -.995 |
| 32. Zafr2 | .145 | .262 | .015 | .057 | .086 | .011 | .237 | .243 | .066 | .019 | .038 | .031 | .039 | .086 | .111 | .145 | .030 | .109 | .111 | .304 | .148 | .444 | .713 | .304 | .604 | .346 | .403 | .040 | .492 | .329 | .322 | - |
| 33. Zafr3 | .189 | .359 | .018 | .073 | .111 | .014 | .318 | .325 | .084 | .024 | .048 | .038 | .050 | .111 | .144 | .191 | .038 | .143 | .144 | .407 | .194 | .591 | .943 | .407 | .810 | .143 | .551 | .051 | .740 | .147 | .143 | .974 |
| 34. Zgab1 | .085 | .146 | .024 | .092 | .143 | .007 | .358 | .136 | .107 | .031 | .024 | .019 | .024 | .051 | .066 | .083 | .050 | .252 | .066 | .170 | .085 | .249 | .403 | .170 | .336 | .065 | .219 | .066 | .085 | .302 | .064 | .067 |
| 35. Zgab2 | .050 | .085 | .015 | .056 | .085 | .005 | .217 | .082 | .064 | .019 | .015 | .012 | .015 | .031 | .039 | .050 | .031 | .151 | .039 | .104 | .051 | .155 | .254 | .104 | .208 | .109 | .132 | .040 | .050 | .497 | .105 | .110 |
| 36. Zgab3 | .300 | .497 | .078 | .282 | .446 | .025 | .903 | .398 | .329 | .097 | .083 | .065 | .078 | .170 | .225 | .270 | .167 | .689 | .225 | .471 | .277 | .621 | .916 | .471 | .812 | .221 | .603 | .080 | .300 | .336 | .077 | .213 |
| 37. DmelA1 | .001 | 0 | 0 | .001 | .001 | 0 | .001 | .001 | .001 | 0 | 0 | 0 | 0 | 0 | 0 | .001 | 0 | 0 | 0 | 0 | 0 | 0 | 0 | 0 | 0 | .001 | .001 | .002 | .001 | .001 | .001 | 0 |
| 38. DmelA2 | 0 | 0 | 0 | 0 | 0 | 0 | 0 | 0 | .001 | 0 | 0 | 0 | 0 | 0 | 0 | 0 | 0 | 0 | 0 | 0 | 0 | 0 | 0 | 0 | 0 | 0 | 0 | .001 | 0 | 0 | 0 | 0 |
| 39. DmelA3 | 0 | 0 | 0 | 0 | .001 | 0 | .001 | .001 | .001 | 0 | 0 | 0 | 0 | 0 | 0 | .001 | 0 | 0 | 0 | 0 | 0 | 0 | 0 | 0 | 0 | 0 | 0 | .001 | 0 | .001 | .001 | 0 |
| 40. DsimA1 | 0 | 0 | 0 | .001 | .001 | 0 | .001 | 0 | .001 | 0 | 0 | 0 | 0 | 0 | 0 | .001 | 0 | 0 | 0 | 0 | 0 | 0 | 0 | 0 | 0 | 0 | .001 | .002 | 0 | .001 | .001 | 0 |
| 41. DereA1 | 0 | 0 | 0 | .001 | .001 | 0 | .001 | .001 | .001 | 0 | 0 | 0 | 0 | 0 | 0 | .001 | 0 | 0 | 0 | 0 | 0 | 0 | 0 | 0 | 0 | 0 | .001 | .002 | 0 | .001 | .001 | 0 |
| 42. Ztub1 | 0 | 0 | 0 | 0 | 0 | 0 | 0 | 0 | 0 | 0 | 0 | 0 | 0 | 0 | 0 | 0 | 0 | 0 | 0 | 0 | 0 | 0 | 0 | 0 | 0 | 0 | 0 | 0 | 0 | 0 | 0 | 0 |
| 43. DmelA5 | 0 | 0 | 0 | 0 | 0 | 0 | 0 | 0 | 0 | 0 | 0 | 0 | 0 | 0 | 0 | 0 | 0 | 0 | 0 | 0 | 0 | 0 | 0 | 0 | 0 | 0 | 0 | 0 | 0 | 0 | 0 | 0 |
| 44. DmelA6 | 0 | 0 | 0 | 0 | 0 | 0 | 0 | 0 | 0 | 0 | 0 | 0 | 0 | 0 | 0 | 0 | 0 | 0 | 0 | 0 | 0 | 0 | 0 | 0 | 0 | 0 | 0 | 0 | 0 | 0 | 0 | 0 |
| 45. DyakA1 | 0 | 0 | 0 | 0 | 0 | 0 | 0 | 0 | 0 | 0 | 0 | 0 | 0 | 0 | 0 | 0 | 0 | 0 | 0 | 0 | 0 | 0 | 0 | 0 | 0 | 0 | 0 | 0 | 0 | 0 | 0 | 0 |
| 46. DyakA2 | 0 | 0 | 0 | 0 | 0 | 0 | 0 | 0 | 0 | 0 | 0 | 0 | 0 | 0 | 0 | 0 | 0 | 0 | 0 | 0 | 0 | 0 | 0 | 0 | 0 | 0 | 0 | 0 | 0 | 0 | 0 | 0 |
| 47. DyakA3 | 0 | 0 | 0 | 0 | 0 | 0 | 0 | 0 | 0 | 0 | 0 | 0 | 0 | 0 | 0 | 0 | 0 | 0 | 0 | 0 | 0 | 0 | 0 | 0 | 0 | 0 | 0 | 0 | 0 | 0 | 0 | 0 |
| 48. DyakA4 | 0 | 0 | 0 | 0 | 0 | 0 | 0 | 0 | 0 | 0 | 0 | 0 | 0 | 0 | 0 | 0 | 0 | 0 | 0 | 0 | 0 | 0 | 0 | 0 | 0 | 0 | 0 | 0 | 0 | 0 | 0 | 0 |
| 49. DyakA5 | 0 | 0 | 0 | 0 | 0 | 0 | 0 | 0 | 0 | 0 | 0 | 0 | 0 | 0 | 0 | 0 | 0 | 0 | 0 | 0 | 0 | 0 | 0 | 0 | 0 | 0 | 0 | 0 | 0 | 0 | 0 | 0 |
| 50. DereA3 | 0 | 0 | 0 | 0 | 0 | 0 | 0 | 0 | 0 | 0 | 0 | 0 | 0 | 0 | 0 | 0 | 0 | 0 | 0 | 0 | 0 | 0 | 0 | 0 | 0 | 0 | 0 | 0 | 0 | 0 | 0 | 0 |
| 51. DereA4 | 0 | 0 | 0 | 0 | 0 | 0 | 0 | 0 | 0 | 0 | 0 | 0 | 0 | 0 | 0 | 0 | 0 | 0 | 0 | 0 | 0 | 0 | 0 | 0 | 0 | 0 | 0 | 0 | 0 | 0 | 0 | 0 |
| 52. DereA5 | 0 | 0 | 0 | 0 | 0 | 0 | 0 | 0 | 0 | 0 | 0 | 0 | 0 | 0 | 0 | 0 | 0 | 0 | 0 | 0 | 0 | 0 | 0 | 0 | 0 | 0 | 0 | 0 | 0 | 0 | 0 | 0 |
| 53. DereA8 | 0 | 0 | 0 | 0 | 0 | 0 | 0 | 0 | 0 | 0 | 0 | 0 | 0 | 0 | 0 | 0 | 0 | 0 | 0 | 0 | 0 | 0 | 0 | 0 | 0 | 0 | 0 | 0 | 0 | 0 | 0 | 0 |
| 54. DereB1 | 0 | 0 | 0 | 0 | 0 | 0 | 0 | 0 | 0 | 0 | 0 | 0 | 0 | 0 | 0 | 0 | 0 | 0 | 0 | 0 | 0 | 0 | 0 | 0 | 0 | 0 | 0 | 0 | 0 | 0 | 0 | 0 |
| 55. DereB8 | 0 | 0 | 0 | 0 | 0 | 0 | 0 | 0 | 0 | 0 | 0 | 0 | 0 | 0 | 0 | 0 | 0 | 0 | 0 | 0 | 0 | .001 | .001 | 0 | .001 | 0 | 0 | 0 | 0 | 0 | 0 | 0 |
| 56. DereB10 | 0 | 0 | 0 | 0 | 0 | 0 | 0 | 0 | 0 | 0 | 0 | 0 | 0 | 0 | 0 | 0 | 0 | 0 | 0 | 0 | 0 | 0 | 0 | 0 | 0 | 0 | 0 | 0 | 0 | 0 | 0 | 0 |
| 57. Ztub2 | 0 | 0 | 0 | 0 | 0 | 0 | 0 | 0 | 0 | 0 | 0 | 0 | 0 | 0 | 0 | 0 | 0 | 0 | 0 | 0 | 0 | 0 | 0 | 0 | 0 | 0 | 0 | 0 | 0 | 0 | 0 | 0 |
| 58. Ztub3 | 0 | 0 | 0 | 0 | 0 | 0 | 0 | 0 | 0 | 0 | 0 | 0 | 0 | 0 | 0 | 0 | 0 | 0 | 0 | 0 | 0 | 0 | 0 | 0 | 0 | 0 | 0 | 0 | 0 | 0 | 0 | 0 |
| 59. Zcam1 | 0 | 0 | 0 | 0 | 0 | 0 | 0 | 0 | 0 | 0 | 0 | 0 | 0 | 0 | 0 | 0 | 0 | 0 | 0 | 0 | 0 | 0 | 0 | 0 | 0 | 0 | 0 | 0 | 0 | 0 | 0 | 0 |
| 60. Zcam2 | 0 | 0 | 0 | 0 | 0 | 0 | 0 | 0 | 0 | 0 | 0 | 0 | 0 | 0 | 0 | 0 | 0 | 0 | 0 | 0 | 0 | 0 | 0 | 0 | 0 | 0 | 0 | 0 | 0 | 0 | 0 | 0 |
| 61. Zcam3 | 0 | 0 | 0 | 0 | 0 | 0 | 0 | 0 | 0 | 0 | 0 | 0 | 0 | 0 | 0 | 0 | 0 | 0 | 0 | 0 | 0 | 0 | .001 | 0 | .001 | 0 | 0 | 0 | 0 | 0 | 0 | 0 |
| 62. Zdav1 | 0 | 0 | 0 | 0 | 0 | 0 | 0 | 0 | 0 | 0 | 0 | 0 | 0 | 0 | 0 | 0 | 0 | 0 | 0 | 0 | 0 | 0 | .001 | 0 | 0 | 0 | 0 | 0 | 0 | 0 | 0 | 0 |

**Additional file 7, continuation.**

|  | 33 | 34 | 35 | 36 | 37 | 38 | 39 | 40 | 41 | 42 | 43 | 44 | 45 | 46 | 47 | 48 | 49 | 50 | 51 | 52 | 53 | 54 | 55 | 56 | 57 | 58 | 59 | 60 | 61 | 62 |
| --- | --- | --- | --- | --- | --- | --- | --- | --- | --- | --- | --- | --- | --- | --- | --- | --- | --- | --- | --- | --- | --- | --- | --- | --- | --- | --- | --- | --- | --- | --- |
| 1. DmelA7 | -1.321 | -1.736 | -1.979 | -1.041 | -3.563 | -3.745 | -3.654 | -3.764 | -3.603 | -3.931 | -4.330 | -4.444 | -4.212 | -4.281 | -4.212 | -4.212 | -4.212 | -4.330 | -4.279 | -4.330 | -4.097 | -4.128 | -3.912 | -3.915 | -4.271 | -4.271 | -4.074 | -4.244 | -3.971 | -4.024 |
| 2. DmelB5 | -.921 | -1.463 | -1.738 | -.682 | -3.723 | -3.876 | -3.787 | -3.764 | -3.764 | -4.024 | -4.263 | -4.388 | -4.137 | -4.212 | -4.137 | -4.137 | -4.137 | -4.263 | -4.210 | -4.263 | -4.022 | -4.059 | -3.822 | -3.995 | -4.211 | -4.211 | -3.995 | -4.176 | -3.889 | -3.939 |
| 3. DsimA2 | -2.395 | -2.283 | -2.469 | -1.775 | -3.967 | -4.073 | -3.894 | -4.012 | -4.012 | -4.109 | -4.537 | -4.445 | -4.458 | -4.501 | -4.458 | -4.458 | -4.458 | -4.537 | -4.497 | -4.537 | -4.335 | -4.057 | -4.227 | -4.215 | -4.195 | -4.195 | -4.338 | -4.455 | -4.208 | -4.366 |
| 4. DsimA3 | -1.806 | -1.696 | -1.931 | -1.081 | -3.493 | -3.730 | -3.581 | -3.533 | -3.533 | -3.850 | -4.309 | -4.182 | -4.203 | -4.266 | -4.203 | -4.203 | -4.203 | -4.309 | -4.264 | -4.309 | -4.100 | -4.125 | -3.935 | -4.077 | -4.143 | -4.143 | -4.082 | -4.232 | -3.990 | -4.106 |
| 5. DsimA4 | -1.607 | -1.474 | -1.737 | -.765 | -3.350 | -3.636 | -3.469 | -3.389 | -3.389 | -3.787 | -4.294 | -4.292 | -4.178 | -4.247 | -4.178 | -4.178 | -4.178 | -4.294 | -4.245 | -4.294 | -4.071 | -4.102 | -3.888 | -4.092 | -4.240 | -4.240 | -4.047 | -4.213 | -3.949 | -4.074 |
| 6. DsimB2 | -2.497 | -2.721 | -2.878 | -2.277 | -3.817 | -3.979 | -3.855 | -3.859 | -3.859 | -4.053 | -4.503 | -4.406 | -4.420 | -4.466 | -4.420 | -4.420 | -4.420 | -4.503 | -4.462 | -4.503 | -4.341 | -4.264 | -4.183 | -4.276 | -4.239 | -4.239 | -4.299 | -4.421 | -4.210 | -4.328 |
| 7. DsimB3 | -1.003 | -.922 | -1.242 | -.123 | -3.483 | -3.697 | -3.548 | -3.498 | -3.498 | -3.819 | -4.123 | -4.122 | -3.996 | -4.073 | -3.996 | -3.996 | -3.996 | -4.123 | -4.073 | -4.123 | -3.902 | -3.944 | -3.692 | -3.878 | -4.178 | -4.178 | -3.868 | -4.047 | -3.770 | -3.891 |
| 8. DsimB4 | -.989 | -1.500 | -1.752 | -.849 | -3.478 | -3.718 | -3.568 | -3.811 | -3.518 | -3.841 | -4.125 | -4.124 | -3.996 | -4.074 | -3.996 | -3.996 | -3.996 | -4.125 | -4.074 | -4.125 | -3.899 | -3.942 | -3.687 | -3.790 | -4.091 | -4.091 | -3.865 | -4.047 | -3.765 | -3.801 |
| 9. DsimB6 | -1.740 | -1.624 | -1.867 | -.980 | -3.259 | -3.561 | -3.388 | -3.298 | -3.298 | -3.721 | -4.243 | -4.241 | -4.125 | -4.196 | -4.125 | -4.125 | -4.125 | -4.243 | -4.194 | -4.243 | -3.950 | -4.057 | -3.835 | -3.999 | -4.195 | -4.195 | -3.997 | -4.164 | -3.901 | -4.022 |
| 1. DsimB7 | -2.289 | -2.185 | -2.377 | -1.673 | -3.948 | -4.051 | -3.946 | -3.993 | -3.993 | -4.086 | -4.405 | -4.289 | -4.307 | -4.364 | -4.307 | -4.307 | -4.307 | -4.405 | -4.361 | -4.405 | -4.196 | -4.012 | -4.047 | -4.215 | -4.152 | -4.152 | -4.184 | -4.326 | -4.046 | -4.210 |
| 11. DsecA1 | -2.001 | -2.285 | -2.475 | -1.747 | -3.951 | -4.082 | -3.968 | -3.995 | -3.995 | -4.136 | -4.401 | -4.398 | -4.296 | -4.358 | -4.296 | -4.296 | -4.296 | -4.401 | -4.355 | -4.401 | -4.241 | -4.146 | -4.022 | -4.158 | -4.096 | -4.096 | -4.167 | -4.319 | -4.071 | -4.195 |
| 12. DsecA2 | -2.096 | -2.372 | -2.553 | -1.865 | -3.905 | -4.047 | -3.929 | -3.949 | -3.949 | -4.108 | -4.462 | -4.458 | -4.369 | -4.422 | -4.369 | -4.369 | -4.369 | -4.462 | -4.419 | -4.462 | -4.253 | -4.163 | -4.119 | -4.229 | -4.182 | -4.182 | -4.246 | -4.381 | -4.155 | -4.274 |
| 13. DsecA3 | -1.981 | -2.283 | -2.469 | -1.775 | -3.701 | -3.890 | -3.758 | -3.743 | -3.743 | -3.981 | -4.361 | -4.241 | -4.260 | -4.319 | -4.260 | -4.260 | -4.260 | -4.361 | -4.317 | -4.361 | -4.290 | -4.093 | -4.096 | -4.129 | -4.070 | -4.070 | -4.138 | -4.368 | -4.136 | -4.256 |
| 14. DsecA4 | -1.607 | -1.968 | -2.182 | -1.380 | -4.094 | -4.168 | -4.070 | -4.141 | -4.141 | -4.184 | -4.410 | -4.407 | -4.310 | -4.368 | -4.310 | -4.310 | -4.310 | -4.410 | -4.365 | -4.410 | -4.198 | -4.102 | -4.047 | -4.173 | -4.118 | -4.118 | -4.185 | -4.330 | -4.092 | -4.212 |
| 15. DsecA5 | -1.470 | -1.855 | -2.083 | -1.220 | -3.841 | -4.004 | -3.880 | -3.883 | -3.883 | -4.079 | -4.279 | -4.398 | -4.158 | -4.230 | -4.158 | -4.158 | -4.158 | -4.279 | -4.228 | -4.279 | -4.118 | -4.011 | -3.855 | -4.021 | -4.096 | -4.096 | -4.022 | -4.196 | -3.920 | -4.049 |
| 16. DsecA7 | -1.314 | -1.746 | -1.981 | -1.109 | -3.445 | -3.704 | -3.547 | -3.485 | -3.485 | -3.839 | -4.349 | -4.347 | -4.244 | -4.306 | -4.244 | -4.244 | -4.244 | -4.349 | -4.304 | -4.349 | -4.136 | -4.160 | -3.971 | -4.112 | -4.179 | -4.179 | -4.118 | -4.270 | -4.024 | -4.144 |
| 17. DsecA8 | -2.103 | -1.976 | -2.189 | -1.391 | -3.636 | -3.845 | -3.706 | -3.676 | -3.676 | -3.736 | -4.405 | -4.402 | -4.307 | -4.364 | -4.307 | -4.307 | -4.307 | -4.405 | -4.361 | -4.405 | -4.196 | -4.214 | -4.047 | -4.121 | -4.332 | -4.332 | -4.184 | -4.326 | -4.091 | -4.210 |
| 18. DsecA9 | -1.475 | -1.151 | -1.445 | -.401 | -3.763 | -3.925 | -3.802 | -3.805 | -3.805 | -3.997 | -4.240 | -4.172 | -4.124 | -4.194 | -4.124 | -4.124 | -4.124 | -4.240 | -4.192 | -4.240 | -4.024 | -4.057 | -3.838 | -4.000 | -4.070 | -4.070 | -3.998 | -4.163 | -3.903 | -4.022 |
| 19. DsecA10 | -1.470 | -1.855 | -2.083 | -1.220 | -3.841 | -4.004 | -3.880 | -3.883 | -3.883 | -4.079 | -4.279 | -4.398 | -4.158 | -4.230 | -4.158 | -4.158 | -4.158 | -4.279 | -4.228 | -4.279 | -4.118 | -4.011 | -3.855 | -4.021 | -4.096 | -4.096 | -4.022 | -4.196 | -3.920 | -4.049 |
| 20. DsecA11 | -.831 | -1.380 | -1.640 | -.723 | -3.761 | -3.917 | -3.795 | -3.803 | -3.803 | -4.007 | -4.074 | -4.073 | -3.941 | -4.022 | -3.941 | -3.941 | -3.941 | -4.074 | -4.022 | -4.074 | -3.849 | -3.894 | -3.629 | -3.645 | -4.046 | -4.046 | -3.811 | -3.998 | -3.713 | -3.744 |
| 21. DsecB11 | -1.305 | -1.734 | -1.970 | -1.093 | -3.825 | -3.985 | -3.862 | -3.868 | -3.868 | -4.058 | -4.228 | -4.352 | -4.104 | -4.178 | -4.104 | -4.104 | -4.104 | -4.228 | -4.177 | -4.228 | -4.071 | -3.962 | -3.799 | -3.972 | -4.047 | -4.047 | -3.969 | -4.146 | -3.868 | -3.996 |
| 22. DsecB12 | -.539 | -1.159 | -1.433 | -.496 | -3.790 | -3.843 | -3.807 | -3.834 | -3.834 | -3.969 | -3.971 | -3.972 | -3.834 | -3.919 | -3.834 | -3.834 | -3.834 | -3.971 | -3.920 | -3.971 | -3.753 | -3.803 | -3.520 | -3.727 | -3.804 | -3.804 | -3.709 | -3.900 | -3.613 | -3.635 |
| 23. DsecB17 | -.072 | -.840 | -1.147 | -.106 | -3.611 | -3.797 | -3.666 | -3.653 | -3.653 | -3.904 | -3.887 | -3.888 | -3.739 | -3.831 | -3.739 | -3.739 | -3.739 | -3.887 | -3.833 | -3.887 | -3.658 | -3.715 | -3.406 | -3.631 | -3.881 | -3.881 | -3.609 | -3.814 | -3.508 | -3.528 |
| 24. DsecB18 | -.831 | -1.380 | -1.640 | -.723 | -3.761 | -3.917 | -3.795 | -3.803 | -3.803 | -3.986 | -4.074 | -4.073 | -3.941 | -4.022 | -3.941 | -3.941 | -3.941 | -4.074 | -4.022 | -4.074 | -3.849 | -3.894 | -3.629 | -3.645 | -4.046 | -4.046 | -3.811 | -3.998 | -3.713 | -3.744 |
| 25. DsecB19 | -.240 | -.967 | -1.266 | -.238 | -3.617 | -3.808 | -3.675 | -3.659 | -3.659 | -3.918 | -3.942 | -3.942 | -3.797 | -3.886 | -3.797 | -3.797 | -3.797 | -3.942 | -3.888 | -3.942 | -3.712 | -3.766 | -3.467 | -3.685 | -3.929 | -3.929 | -3.665 | -3.867 | -3.564 | -3.588 |
| 26. Zdav2 | -1.475 | -1.863 | -1.615 | -1.231 | -3.562 | -3.737 | -3.647 | -3.603 | -3.603 | -4.000 | -4.284 | -4.398 | -4.166 | -4.236 | -4.166 | -4.166 | -4.166 | -4.284 | -4.234 | -4.284 | -4.059 | -4.092 | -3.873 | -4.034 | -4.106 | -4.231 | -4.035 | -4.061 | -3.766 | -3.807 |
| 27. Zdav3 | -.598 | -1.235 | -1.516 | -.521 | -3.506 | -3.686 | -3.595 | -3.546 | -3.520 | -3.867 | -4.061 | -4.200 | -3.927 | -4.009 | -3.927 | -3.927 | -3.927 | -4.061 | -4.009 | -4.061 | -3.936 | -3.977 | -3.614 | -3.811 | -4.035 | -4.035 | -3.798 | -3.985 | -3.699 | -3.729 |
| 28. Zind1 | -1.975 | -1.856 | -2.077 | -1.764 | -3.117 | -3.368 | -3.270 | -3.156 | -3.156 | -3.640 | -4.308 | -4.414 | -4.197 | -4.263 | -4.197 | -4.197 | -4.197 | -4.308 | -4.261 | -4.308 | -4.092 | -4.120 | -3.918 | -4.066 | -4.251 | -4.251 | -4.071 | -4.229 | -3.976 | -4.022 |
| 29. Zind2 | -.333 | -1.736 | -1.979 | -1.041 | -3.563 | -3.745 | -3.654 | -3.603 | -3.603 | -3.931 | -4.330 | -4.444 | -4.057 | -4.137 | -4.057 | -4.057 | -4.057 | -4.330 | -4.279 | -4.330 | -4.097 | -4.128 | -3.912 | -3.915 | -4.145 | -4.145 | -4.074 | -4.244 | -3.971 | -4.024 |
| 30. Zind3 | -1.461 | -1.037 | -.682 | -.967 | -3.429 | -3.622 | -3.530 | -3.469 | -3.469 | -3.917 | -4.253 | -4.367 | -4.062 | -4.137 | -4.062 | -4.062 | -4.062 | -4.253 | -4.205 | -4.253 | -4.035 | -4.067 | -3.850 | -3.936 | -4.011 | -4.146 | -4.010 | -4.035 | -3.745 | -3.872 |
| 31. Zafr1 | -1.473 | -1.870 | -1.633 | -1.783 | -3.397 | -3.591 | -3.500 | -3.437 | -3.437 | -3.887 | -4.249 | -4.359 | -3.982 | -4.061 | -3.982 | -3.982 | -3.982 | -4.249 | -4.202 | -4.249 | -4.035 | -4.067 | -3.853 | -3.861 | -3.937 | -4.080 | -4.011 | -4.035 | -3.751 | -3.785 |
| 32. Zafr2 | -.033 | -1.848 | -1.610 | -1.252 | -3.725 | -3.866 | -3.778 | -3.767 | -3.767 | -4.063 | -4.405 | -4.503 | -4.159 | -4.232 | -4.159 | -4.159 | -4.159 | -4.405 | -4.358 | -4.405 | -4.184 | -4.207 | -4.022 | -4.020 | -3.942 | -4.097 | -4.265 | -4.197 | -3.918 | -3.968 |
| 33. Zafr3 | - | -1.732 | -1.968 | -1.216 | -3.713 | -3.859 | -3.771 | -3.754 | -3.754 | -3.999 | -4.393 | -4.497 | -4.138 | -4.213 | -4.138 | -4.138 | -4.138 | -4.393 | -4.344 | -4.393 | -4.165 | -4.191 | -3.996 | -3.995 | -4.073 | -4.073 | -4.148 | -4.308 | -4.047 | -4.103 |
| 34. Zgab1 | .086 | - | -.337 | .480 | -3.429 | -3.456 | -3.530 | -3.469 | -3.469 | -3.820 | -4.147 | -4.273 | -4.022 | -4.097 | -4.022 | -4.022 | -4.022 | -4.147 | -4.097 | -4.147 | -3.927 | -3.966 | -3.724 | -3.901 | -4.220 | -4.220 | -3.895 | -4.071 | -3.799 | -3.919 |
| 35. Zgab2 | .051 | .737 | - | -.270 | -3.381 | -3.574 | -3.483 | -3.421 | -3.421 | -3.867 | -4.213 | -4.327 | -4.097 | -4.167 | -4.097 | -4.097 | -4.097 | -4.213 | -4.166 | -4.213 | -4.001 | -4.033 | -3.814 | -3.975 | -4.168 | -4.263 | -3.974 | -3.998 | -3.713 | -3.835 |
| 36. Zgab3 | .226 | .632 | .787 | - | -3.509 | -3.664 | -3.576 | -3.564 | -3.550 | -3.812 | -3.918 | -4.071 | -3.776 | -3.864 | -3.776 | -3.776 | -3.776 | -3.918 | -3.970 | -3.918 | -3.698 | -3.751 | -3.456 | -3.671 | -4.043 | -4.043 | -3.651 | -3.847 | -3.554 | -3.671 |
| 37. DmelA1 | 0 | .001 | .001 | .001 | - | -1.253 | 1.252 | -1.407 | 1.043 | -1.897 | -4.160 | -4.081 | -4.218 | -4.218 | -4.177 | -4.123 | -4.218 | -4.160 | -4.138 | -4.160 | -4.030 | -4.114 | -4.101 | -4.079 | -4.129 | -4.094 | -4.141 | -4.148 | -4.034 | -4.098 |
| 38. DmelA2 | 0 | .001 | .001 | 0 | .213 | - | -1.264 | -2.218 | -1.411 | -2.569 | -4.207 | -4.144 | -4.278 | -4.278 | -4.247 | -4.202 | -4.278 | -4.207 | -4.184 | -4.207 | -3.971 | -4.122 | -4.154 | -4.117 | -4.160 | -4.125 | -4.188 | -4.208 | -4.091 | -4.168 |
| 39. DmelA3 | 0 | .001 | .001 | .001 | .213 | .209 | - | -.746 | 2.979 | -1.368 | -4.015 | -3.915 | -4.113 | -4.113 | -4.057 | -3.984 | -4.113 | -4.015 | -3.995 | -4.015 | -3.884 | -3.986 | -4.009 | -3.969 | -4.066 | -4.026 | -4.060 | -4.043 | -3.933 | -3.974 |
| 40. DsimA1 | 0 | .001 | .001 | .001 | .162 | .028 | .457 | - | -1.137 | -1.144 | -4.059 | -3.931 | -4.160 | -4.160 | -4.084 | -3.991 | -4.160 | -4.059 | -4.059 | -4.059 | -3.899 | -4.104 | -4.067 | -4.117 | -4.181 | -4.144 | -4.131 | -4.087 | -3.979 | -3.996 |
| 41. DereA1 | 0 | .001 | .001 | .001 | .299 | .161 | .004 | .258 | - | -1.688 | -4.059 | -3.931 | -4.180 | -4.180 | -4.103 | -4.008 | -4.180 | -4.059 | -4.040 | -4.059 | -3.899 | -4.082 | -4.088 | -4.109 | -4.205 | -4.167 | -4.153 | -4.107 | -3.999 | -4.014 |
| 42. Ztub1 | 0 | 0 | 0 | 0 | .060 | .011 | .174 | .255 | .094 | - | -4.056 | -3.952 | -4.136 | -4.136 | -4.077 | -4.003 | -4.136 | -4.056 | -4.035 | -4.056 | -3.902 | -4.056 | -4.032 | -4.028 | -4.125 | -4.094 | -4.084 | -4.110 | -3.997 | -4.055 |
| 43. DmelA5 | 0 | 0 | 0 | 0 | 0 | 0 | 0 | 0 | 0 | 0 | - | -1.981 | -1.800 | -1.800 | -1.509 | -1.800 | -1.800 | -1.981 | -1.801 | -1.981 | -2.456 | -3.279 | -1.437 | -3.051 | -3.725 | -3.725 | -2.168 | -2.593 | -1.587 | -2.211 |
| 44. DmelA6 | 0 | 0 | 0 | 0 | 0 | 0 | 0 | 0 | 0 | 0 | .050 | - | -2.269 | -2.269 | -2.050 | -2.269 | -2.269 | -2.412 | -2.270 | -2.412 | -2.797 | -3.515 | -1.959 | -3.316 | -3.726 | -3.726 | -2.554 | -2.922 | -2.056 | -2.596 |
| 45. DyakA1 | 0 | 0 | 0 | 0 | 0 | 0 | 0 | 0 | 0 | 0 | .074 | .025 | - | -1.408 | -.998 | -1.408 | .000 | -1.800 | -1.622 | -1.800 | -2.173 | -3.090 | -.913 | -2.513 | -3.349 | -3.349 | -1.840 | -2.336 | -1.144 | -1.893 |
| 46. DyakA2 | 0 | 0 | 0 | 0 | 0 | 0 | 0 | 0 | 0 | 0 | .074 | .025 | .162 | - | -.998 | -1.408 | -1.408 | -2.268 | -2.126 | -2.268 | -2.555 | -3.090 | -1.583 | -2.513 | -3.349 | -3.349 | -2.284 | -2.703 | -1.705 | -2.337 |
| 47. DyakA3 | 0 | 0 | 0 | 0 | 0 | 0 | 0 | 0 | 0 | 0 | .134 | .043 | .320 | .320 | - | -.998 | -.998 | -2.049 | -1.891 | -2.049 | -2.372 | -3.222 | -1.279 | -2.682 | -3.468 | -3.468 | -2.073 | -2.527 | -1.442 | -2.127 |
| 48. DyakA4 | 0 | 0 | 0 | 0 | 0 | 0 | 0 | 0 | 0 | 0 | .074 | .025 | .162 | .162 | .320 | - | -1.408 | -2.268 | -2.126 | -2.268 | -2.555 | -3.346 | -1.583 | -2.841 | -3.580 | -3.580 | -2.284 | -2.703 | -1.705 | -2.337 |
| 49. DyakA5 | 0 | 0 | 0 | 0 | 0 | 0 | 0 | 0 | 0 | 0 | .074 | .025 | 1.000 | .162 | .320 | .162 | - | -1.800 | -1.622 | -1.800 | -2.173 | -3.090 | -.913 | -2.513 | -3.349 | -3.349 | -1.840 | -2.336 | -1.144 | -1.893 |
| 50. DereA3 | 0 | 0 | 0 | 0 | 0 | 0 | 0 | 0 | 0 | 0 | .050 | .017 | .074 | .025 | .043 | .025 | .074 | - | .999 | .000 | -2.267 | -3.279 | -1.117 | -3.051 | -3.725 | -3.725 | -1.949 | -2.410 | -1.006 | -1.989 |
| 51. DereA4 | 0 | 0 | 0 | 0 | 0 | 0 | 0 | 0 | 0 | 0 | .074 | .025 | .107 | .036 | .061 | .036 | .107 | .320 | - | .999 | -2.158 | -3.209 | -.954 | -2.970 | -3.662 | -3.662 | -1.821 | -2.291 | -.864 | -1.851 |
| 52. DereA5 | 0 | 0 | 0 | 0 | 0 | 0 | 0 | 0 | 0 | 0 | .050 | .017 | .074 | .025 | .043 | .025 | .074 | 1.000 | .320 | - | -2.267 | -3.279 | -1.117 | -3.051 | -3.725 | -3.725 | -1.949 | -2.410 | -1.006 | -1.989 |
| 53. DereA8 | 0 | 0 | 0 | 0 | 0 | 0 | 0 | 0 | 0 | 0 | .015 | .006 | .032 | .012 | .019 | .012 | .032 | .025 | .033 | .025 | - | -2.497 | -1.152 | -3.281 | -3.866 | -3.866 | -1.707 | -2.377 | -1.612 | -1.963 |
| 54. DereB1 | 0 | 0 | 0 | 0 | 0 | 0 | 0 | 0 | 0 | 0 | .001 | .001 | .002 | .002 | .002 | .001 | .002 | .001 | .002 | .001 | .014 | - | -2.879 | -1.531 | -3.103 | -2.816 | -3.061 | -3.286 | -2.737 | -3.288 |
| 55. DereB8 | 0 | 0 | 0 | .001 | 0 | 0 | 0 | 0 | 0 | 0 | .153 | .052 | .363 | .116 | .203 | .116 | .363 | .266 | .342 | .266 | .252 | .005 | - | -2.655 | -3.428 | -3.428 | -.187 | -1.286 | -.040 | -.451 |
| 56. DereB10 | 0 | 0 | 0 | 0 | 0 | 0 | 0 | 0 | 0 | 0 | .003 | .001 | .013 | .013 | .008 | .005 | .013 | .003 | .004 | .003 | .001 | .128 | .009 | - | -2.139 | -1.702 | -2.802 | -3.110 | -2.409 | -3.112 |
| 57. Ztub2 | 0 | 0 | 0 | 0 | 0 | 0 | 0 | 0 | 0 | 0 | 0 | 0 | .001 | .001 | .001 | 0 | .001 | 0 | 0 | 0 | 0 | .002 | .001 | .034 | - | -1.408 | -3.534 | -3.559 | -3.050 | -3.560 |
| 58. Ztub3 | 0 | 0 | 0 | 0 | 0 | 0 | 0 | 0 | 0 | 0 | 0 | 0 | .001 | .001 | .001 | 0 | .001 | 0 | 0 | 0 | 0 | .006 | .001 | .091 | .162 | - | -3.534 | -3.762 | -3.303 | -3.763 |
| 59. Zcam1 | 0 | 0 | 0 | 0 | 0 | 0 | 0 | 0 | 0 | 0 | .032 | .012 | .068 | .024 | .040 | .024 | .068 | .054 | .071 | .054 | .090 | .003 | .852 | .006 | .001 | .001 | - | -1.283 | -.179 | -1.286 |
| 60. Zcam2 | 0 | 0 | 0 | 0 | 0 | 0 | 0 | 0 | 0 | 0 | .011 | .004 | .021 | .008 | .013 | .008 | .021 | .017 | .024 | .017 | .019 | .001 | .201 | .002 | .001 | 0 | .202 | - | -.382 | -1.628 |
| 61. Zcam3 | 0 | 0 | 0 | .001 | 0 | 0 | 0 | 0 | 0 | 0 | .115 | .042 | .255 | .091 | .152 | .091 | .255 | .316 | .389 | .316 | .109 | .007 | .968 | .017 | .003 | .001 | .858 | .703 | - | -.386 |
| 62. Zdav1 | 0 | 0 | 0 | 0 | 0 | 0 | 0 | 0 | 0 | 0 | .029 | .011 | .061 | .021 | .035 | .021 | .061 | .049 | .067 | .049 | .052 | .001 | .653 | .002 | .001 | 0 | .201 | .106 | .700 | - |

Symbols for species names: Ztub: *Z. tuberculatus*; Zcam: *Z. camerounensis*; Zdav: *Z. davidi*; Zgab: *Z. gabonicus*; Zafr: *Z. africanus*; Zind: *Z. indianus*; Dmel: *D. melanogaster*; Dsim: *D. simulans*; Dsec: *D. sechellia*; Dyak: *D. yakuba*; Dere: *D. erecta*.
