# Supplementary material for: Multiple invasions of Gypsy and Micropia retroelements in genus Zaprionus and melanogaster subgroup of the genus Drosophila
Source: BMC Evol Biol. 2009 Dec 2;9:279. doi: 10.1186/1471-2148-9-279 (PMC2797524; doi:10.1186/1471-2148-9-279)
Supplement: Additional file 9 — Pairwise genetic distance among Micropia sequences of Zaprionus, melanogaster and repleta species. Distances calculated by the MCL method as implemented by MEGA 4.1. [file 1471-2148-9-279-S9.DOC]

**Additional file 9. Pairwise genetic distance among *Micropia* sequences of *Zaprionus*, *melanogaster* and *repleta* species.**

|  | 1 | 2 | 3 | 4 | 5 | 6 | 7 | 8 | 9 | 10 | 11 | 12 | 13 | 14 | 15 | 16 | 17 | 18 | 19 | 20 | 21 | 22 | 23 | 24 | 25 | 26 |
| --- | --- | --- | --- | --- | --- | --- | --- | --- | --- | --- | --- | --- | --- | --- | --- | --- | --- | --- | --- | --- | --- | --- | --- | --- | --- | --- |
| 1. Dser |  |  |  |  |  |  |  |  |  |  |  |  |  |  |  |  |  |  |  |  |  |  |  |  |  |  |
| 2. Dhyd | .065 |  |  |  |  |  |  |  |  |  |  |  |  |  |  |  |  |  |  |  |  |  |  |  |  |  |
| 3. Dkoe | .595 | .578 |  |  |  |  |  |  |  |  |  |  |  |  |  |  |  |  |  |  |  |  |  |  |  |  |
| 4. Dmer | .051 | .037 | .557 |  |  |  |  |  |  |  |  |  |  |  |  |  |  |  |  |  |  |  |  |  |  |  |
| 5. Dspe | .724 | .716 | .592 | .656 |  |  |  |  |  |  |  |  |  |  |  |  |  |  |  |  |  |  |  |  |  |  |
| 6. Dsem | .069 | .087 | .717 | .072 | .825 |  |  |  |  |  |  |  |  |  |  |  |  |  |  |  |  |  |  |  |  |  |
| 7. Dant | .105 | .137 | .753 | .123 | .814 | .106 |  |  |  |  |  |  |  |  |  |  |  |  |  |  |  |  |  |  |  |  |
| 8. Dgou | .071 | .055 | 1.198 | .050 | 1.112 | .050 | .087 |  |  |  |  |  |  |  |  |  |  |  |  |  |  |  |  |  |  |  |
| 9. Dbuz | .025 | .018 | .581 | .007 | .702 | .033 | .089 | .050 |  |  |  |  |  |  |  |  |  |  |  |  |  |  |  |  |  |  |
| 10. Dpar | .035 | .013 | .536 | .000 | .657 | .055 | .104 | .050 | .007 |  |  |  |  |  |  |  |  |  |  |  |  |  |  |  |  |  |
| 11. DmelA1 | .532 | .517 | 1.108 | .503 | 1.037 | .512 | .592 | .548 | .494 | .492 |  |  |  |  |  |  |  |  |  |  |  |  |  |  |  |  |
| 12. DmelA3 | .527 | .511 | 1.100 | .497 | 1.047 | .507 | .585 | .539 | .488 | .487 | .003 |  |  |  |  |  |  |  |  |  |  |  |  |  |  |  |
| 13. DsimA1 | .532 | .517 | 1.108 | .503 | 1.037 | .512 | .592 | .548 | .494 | .492 | .000 | .003 |  |  |  |  |  |  |  |  |  |  |  |  |  |  |
| 14. DsimA2 | .532 | .517 | 1.108 | .503 | 1.037 | .512 | .599 | .548 | .494 | .492 | .006 | .010 | .006 |  |  |  |  |  |  |  |  |  |  |  |  |  |
| 15. DsimA3 | .547 | .532 | 1.149 | .517 | 1.071 | .528 | .601 | .560 | .509 | .507 | .010 | .013 | .010 | .016 |  |  |  |  |  |  |  |  |  |  |  |  |
| 16. DsimA4 | .536 | .526 | 1.123 | .511 | 1.062 | .513 | .595 | .550 | .503 | .501 | .003 | .007 | .003 | .010 | .013 |  |  |  |  |  |  |  |  |  |  |  |
| 17. DsimA5 | .558 | .542 | 1.123 | .527 | 1.059 | .538 | .606 | .587 | .520 | .517 | .016 | .020 | .016 | .023 | .020 | .020 |  |  |  |  |  |  |  |  |  |  |
| 18. DsimA6 | .547 | .532 | 1.149 | .517 | 1.071 | .528 | .601 | .560 | .509 | .507 | .013 | .016 | .013 | .019 | .003 | .016 | .023 |  |  |  |  |  |  |  |  |  |
| 19. DsecA1 | .532 | .517 | 1.108 | .503 | 1.037 | .512 | .592 | .548 | .494 | .492 | .000 | .003 | .000 | .006 | .010 | .003 | .016 | .013 |  |  |  |  |  |  |  |  |
| 20. DsecA2 | .532 | .517 | 1.108 | .503 | 1.037 | .512 | .592 | .548 | .494 | .492 | .000 | .003 | .000 | .006 | .010 | .003 | .016 | .013 | .000 |  |  |  |  |  |  |  |
| 21. DsecA5 | .549 | .533 | 1.081 | .518 | 1.057 | .529 | .614 | .573 | .510 | .508 | .006 | .010 | .006 | .013 | .016 | .010 | .023 | .020 | .006 | .006 |  |  |  |  |  |  |
| 22. DsecA7 | .538 | .523 | 1.117 | .508 | 1.047 | .518 | .599 | .548 | .500 | .498 | .003 | .006 | .003 | .010 | .013 | .007 | .020 | .016 | .003 | .003 | .010 |  |  |  |  |  |
| 23. DsecA8 | .532 | .517 | 1.119 | .503 | 1.043 | .512 | .592 | .548 | .494 | .492 | .000 | .003 | .000 | .006 | .010 | .003 | .016 | .013 | .000 | .000 | .006 | .003 |  |  |  |  |
| 24. DsecA9 | .547 | .532 | 1.149 | .517 | 1.071 | .528 | .601 | .560 | .509 | .507 | .013 | .016 | .013 | .019 | .003 | .016 | .023 | .000 | .013 | .013 | .020 | .016 | .013 |  |  |  |
| 25. DsecA11 | .547 | .532 | 1.149 | .517 | 1.071 | .528 | .584 | .560 | .509 | .507 | .016 | .019 | .016 | .023 | .013 | .020 | .026 | .010 | .016 | .016 | .023 | .019 | .016 | .010 |  |  |
| 26. DsecA18 | .610 | .593 | 1.183 | .595 | 1.062 | .610 | .663 | .671 | .591 | .576 | .058 | .062 | .058 | .065 | .062 | .063 | .069 | .065 | .058 | .058 | .066 | .062 | .058 | .065 | .069 |  |
| 27. DsecA19 | .546 | .514 | 1.151 | .505 | 1.135 | .532 | .596 | .549 | .490 | .497 | .043 | .047 | .043 | .050 | .054 | .047 | .061 | .057 | .043 | .043 | .050 | .047 | .043 | .057 | .061 | .107 |
| 28. DsecA20 | .580 | .553 | 1.098 | .546 | 1.043 | .581 | .630 | .620 | .561 | .533 | .105 | .101 | .105 | .112 | .116 | .110 | .121 | .116 | .105 | .105 | .105 | .108 | .105 | .116 | .120 | .156 |
| 29. DsecB8 | .543 | .528 | 1.135 | .513 | 1.066 | .525 | .588 | .548 | .505 | .503 | .010 | .013 | .010 | .016 | .020 | .013 | .026 | .023 | .010 | .010 | .016 | .013 | .010 | .023 | .023 | .070 |
| 30. DsecB19 | .531 | .515 | 1.113 | .501 | 1.062 | .511 | .581 | .539 | .492 | .490 | .010 | .013 | .010 | .016 | .020 | .013 | .026 | .023 | .010 | .010 | .016 | .013 | .010 | .023 | .026 | .069 |
| 31. DyakA1 | .548 | .519 | 1.016 | .505 | 1.027 | .535 | .585 | .547 | .516 | .494 | .078 | .074 | .078 | .085 | .081 | .082 | .092 | .085 | .078 | .078 | .085 | .081 | .078 | .085 | .095 | .132 |
| 32. DyakA2 | .572 | .543 | 1.101 | .528 | 1.089 | .548 | .629 | .560 | .532 | .518 | .088 | .085 | .088 | .095 | .092 | .093 | .103 | .095 | .088 | .088 | .096 | .092 | .088 | .095 | .106 | .144 |
| 33. DyakA3 | .579 | .552 | 1.122 | .534 | 1.097 | .569 | .627 | .568 | .551 | .527 | .095 | .092 | .095 | .103 | .099 | .100 | .111 | .103 | .095 | .095 | .103 | .099 | .095 | .103 | .114 | .160 |
| 34. DyakB3 | .562 | .521 | .979 | .514 | .977 | .547 | .637 | .541 | .527 | .500 | .118 | .114 | .118 | .118 | .122 | .123 | .135 | .122 | .118 | .118 | .118 | .122 | .118 | .122 | .134 | .163 |
| 35. Ztub1 | .558 | .542 | 1.130 | .527 | 1.053 | .538 | .614 | .579 | .519 | .517 | .020 | .023 | .020 | .026 | .030 | .023 | .037 | .033 | .020 | .020 | .026 | .023 | .020 | .033 | .036 | .080 |
| 36. Ztub2 | .522 | .507 | 1.104 | .493 | 1.052 | .508 | .570 | .536 | .489 | .482 | .013 | .016 | .013 | .020 | .023 | .016 | .030 | .026 | .013 | .013 | .020 | .016 | .013 | .026 | .030 | .073 |
| 37. Ztub3 | .539 | .523 | 1.119 | .509 | 1.086 | .519 | .599 | .552 | .500 | .498 | .016 | .016 | .016 | .023 | .026 | .020 | .033 | .030 | .016 | .016 | .023 | .020 | .016 | .030 | .033 | .073 |
| 38. Zcam1 | .544 | .515 | 1.063 | .500 | 1.062 | .530 | .597 | .540 | .511 | .490 | .067 | .064 | .067 | .067 | .078 | .071 | .085 | .081 | .067 | .067 | .074 | .070 | .067 | .081 | .085 | .129 |
| 39. Zcam2 | .551 | .521 | 1.075 | .507 | 1.076 | .537 | .605 | .550 | .518 | .496 | .071 | .067 | .071 | .071 | .081 | .075 | .089 | .085 | .071 | .071 | .078 | .074 | .071 | .085 | .088 | .133 |
| 40. Zcam3 | .551 | .521 | 1.051 | .507 | 1.076 | .537 | .605 | .550 | .518 | .496 | .071 | .067 | .071 | .071 | .081 | .075 | .089 | .085 | .071 | .071 | .078 | .074 | .071 | .085 | .088 | .133 |
| 41. Zdav1 | .540 | .511 | 1.066 | .497 | 1.096 | .526 | .595 | .518 | .507 | .487 | .081 | .078 | .081 | .088 | .092 | .086 | .096 | .095 | .081 | .081 | .089 | .085 | .081 | .095 | .099 | .144 |
| 42. Zdav2 | .518 | .490 | 1.023 | .476 | 1.046 | .504 | .568 | .514 | .485 | .466 | .070 | .067 | .070 | .077 | .081 | .075 | .085 | .084 | .070 | .070 | .078 | .074 | .070 | .084 | .088 | .132 |
| 43. Zdav3 | .524 | .495 | 1.032 | .482 | 1.056 | .510 | .575 | .506 | .491 | .471 | .074 | .070 | .074 | .081 | .084 | .078 | .088 | .088 | .074 | .074 | .081 | .077 | .074 | .088 | .091 | .136 |
| 44. Zgab1 | .526 | .498 | 1.040 | .484 | 1.066 | .504 | .568 | .514 | .493 | .473 | .074 | .070 | .074 | .081 | .085 | .078 | .088 | .088 | .074 | .074 | .081 | .077 | .074 | .088 | .092 | .136 |
| 45. Zgab2 | .510 | .498 | 1.007 | .484 | 1.046 | .512 | .578 | .526 | .493 | .473 | .074 | .070 | .074 | .081 | .085 | .078 | .088 | .088 | .074 | .074 | .081 | .077 | .074 | .088 | .092 | .128 |
| 46. Zgab3 | .520 | .503 | 1.051 | .478 | 1.073 | .498 | .561 | .514 | .487 | .468 | .077 | .074 | .077 | .084 | .088 | .082 | .092 | .092 | .077 | .077 | .085 | .081 | .077 | .092 | .095 | .140 |
| 47. Zafr1 | .540 | .502 | 1.083 | .495 | 1.064 | .517 | .570 | .512 | .506 | .482 | .077 | .073 | .077 | .084 | .088 | .081 | .092 | .092 | .077 | .077 | .084 | .073 | .077 | .092 | .095 | .133 |
| 48. Zafr2 | .540 | .502 | 1.083 | .495 | 1.064 | .517 | .570 | .512 | .506 | .482 | .077 | .073 | .077 | .084 | .088 | .081 | .092 | .092 | .077 | .077 | .084 | .073 | .077 | .092 | .095 | .133 |
| 49. Zafr3 | .518 | .490 | 1.023 | .476 | 1.046 | .504 | .568 | .514 | .485 | .466 | .070 | .067 | .070 | .077 | .081 | .075 | .085 | .084 | .070 | .070 | .078 | .074 | .070 | .084 | .088 | .132 |
| 50. Zind1 | .519 | .491 | 1.028 | .477 | 1.052 | .505 | .570 | .514 | .486 | .467 | .070 | .067 | .070 | .077 | .081 | .075 | .085 | .085 | .070 | .070 | .078 | .074 | .070 | .085 | .088 | .133 |
| 51. Zind2 | .526 | .498 | 1.040 | .484 | 1.066 | .504 | .568 | .514 | .493 | .473 | .074 | .070 | .074 | .081 | .085 | .078 | .088 | .088 | .074 | .074 | .081 | .077 | .074 | .088 | .092 | .136 |
| 52. Zind3 | .518 | .490 | 1.023 | .476 | 1.046 | .504 | .568 | .514 | .485 | .466 | .070 | .067 | .070 | .077 | .081 | .075 | .085 | .084 | .070 | .070 | .078 | .074 | .070 | .084 | .088 | .132 |

**Additional file 9, continuation.**

|  | 27 | 28 | 29 | 30 | 31 | 32 | 33 | 34 | 35 | 36 | 37 | 38 | 39 | 40 | 41 | 42 | 43 | 44 | 45 | 46 | 47 | 48 | 49 | 50 | 51 |
| --- | --- | --- | --- | --- | --- | --- | --- | --- | --- | --- | --- | --- | --- | --- | --- | --- | --- | --- | --- | --- | --- | --- | --- | --- | --- |
| 28. DsecA20 | .150 |  |  |  |  |  |  |  |  |  |  |  |  |  |  |  |  |  |  |  |  |  |  |  |  |
| 29. DsecB8 | .043 | .117 |  |  |  |  |  |  |  |  |  |  |  |  |  |  |  |  |  |  |  |  |  |  |  |
| 30. DsecB19 | .030 | .117 | .013 |  |  |  |  |  |  |  |  |  |  |  |  |  |  |  |  |  |  |  |  |  |  |
| 31. DyakA1 | .115 | .083 | .085 | .085 |  |  |  |  |  |  |  |  |  |  |  |  |  |  |  |  |  |  |  |  |  |
| 32. DyakA2 | .134 | .094 | .100 | .099 | .043 |  |  |  |  |  |  |  |  |  |  |  |  |  |  |  |  |  |  |  |  |
| 33. DyakA3 | .134 | .098 | .104 | .103 | .036 | .061 |  |  |  |  |  |  |  |  |  |  |  |  |  |  |  |  |  |  |  |
| 34. DyakB3 | .157 | .104 | .127 | .122 | .059 | .086 | .079 |  |  |  |  |  |  |  |  |  |  |  |  |  |  |  |  |  |  |
| 35. Ztub1 | .053 | .121 | .023 | .023 | .089 | .104 | .107 | .131 |  |  |  |  |  |  |  |  |  |  |  |  |  |  |  |  |  |
| 36. Ztub2 | .047 | .106 | .016 | .016 | .075 | .089 | .093 | .115 | .020 |  |  |  |  |  |  |  |  |  |  |  |  |  |  |  |  |
| 37. Ztub3 | .050 | .110 | .020 | .019 | .082 | .096 | .100 | .119 | .023 | .016 |  |  |  |  |  |  |  |  |  |  |  |  |  |  |  |
| 38. Zcam1 | .111 | .075 | .078 | .078 | .043 | .054 | .061 | .059 | .082 | .068 | .075 |  |  |  |  |  |  |  |  |  |  |  |  |  |  |
| 39. Zcam2 | .115 | .079 | .082 | .081 | .047 | .057 | .064 | .063 | .085 | .071 | .078 | .003 |  |  |  |  |  |  |  |  |  |  |  |  |  |
| 40. Zcam3 | .115 | .079 | .082 | .081 | .047 | .057 | .064 | .063 | .085 | .071 | .071 | .003 | .006 |  |  |  |  |  |  |  |  |  |  |  |  |
| 41. Zdav1 | .118 | .078 | .093 | .092 | .043 | .057 | .064 | .082 | .096 | .082 | .089 | .047 | .050 | .050 |  |  |  |  |  |  |  |  |  |  |  |
| 42. Zdav2 | .107 | .075 | .082 | .081 | .033 | .047 | .053 | .074 | .085 | .071 | .078 | .036 | .040 | .040 | .010 |  |  |  |  |  |  |  |  |  |  |
| 43. Zdav3 | .110 | .078 | .085 | .085 | .036 | .050 | .057 | .078 | .089 | .074 | .082 | .040 | .043 | .043 | .006 | .003 |  |  |  |  |  |  |  |  |  |
| 44. Zgab1 | .111 | .078 | .085 | .085 | .036 | .050 | .057 | .078 | .089 | .075 | .082 | .040 | .043 | .043 | .013 | .003 | .006 |  |  |  |  |  |  |  |  |
| 45. Zgab2 | .111 | .078 | .085 | .085 | .036 | .050 | .057 | .078 | .089 | .075 | .082 | .040 | .043 | .043 | .013 | .003 | .006 | .006 |  |  |  |  |  |  |  |
| 46. Zgab3 | .114 | .082 | .089 | .088 | .040 | .054 | .060 | .082 | .092 | .078 | .085 | .043 | .047 | .047 | .016 | .006 | .010 | .003 | .010 |  |  |  |  |  |  |
| 47. Zafr1 | .115 | .086 | .089 | .088 | .041 | .056 | .059 | .088 | .092 | .077 | .081 | .038 | .041 | .041 | .020 | .010 | .013 | .013 | .013 | .017 |  |  |  |  |  |
| 48. Zafr2 | .115 | .086 | .089 | .088 | .041 | .056 | .059 | .088 | .092 | .077 | .081 | .038 | .041 | .041 | .020 | .010 | .013 | .013 | .013 | .017 | .000 |  |  |  |  |
| 49. Zafr3 | .107 | .075 | .082 | .081 | .033 | .047 | .053 | .074 | .085 | .071 | .078 | .036 | .040 | .040 | .010 | .000 | .003 | .003 | .003 | .006 | .010 | .010 |  |  |  |
| 50. Zind1 | .107 | .082 | .082 | .081 | .040 | .054 | .060 | .082 | .092 | .078 | .085 | .043 | .047 | .047 | .016 | .006 | .010 | .010 | .010 | .013 | .017 | .017 | .006 |  |  |
| 51. Zind2 | .111 | .078 | .085 | .085 | .036 | .050 | .057 | .078 | .089 | .075 | .082 | .040 | .043 | .043 | .013 | .003 | .006 | .006 | .006 | .010 | .013 | .013 | .003 | .010 |  |
| 52. Zind3 | .107 | .075 | .082 | .081 | .033 | .047 | .053 | .074 | .085 | .071 | .078 | .036 | .040 | .040 | .010 | .000 | .003 | .003 | .003 | .006 | .010 | .010 | .000 | .006 | .003 |

Symbols for species names: Dser: *D. serido*; Dhyd: *D. hydei*; Dkoe: *D. koepferae*; Dmer: *D. mercatorum*; Dspe: *D. spenceri*; Dsem: *D. seriema*; Dant: *D. antonietae*; Dgou: *D. gouveai*; Dbuz: *D. buzzatii*; Dpar: *D. paranaensis*; Dmel: *D. melanogaster*; Dsim: *D. simulans*; Dsec: *D. sechellia*; Dyak: *D. yakuba*; Ztub: *Z. tuberculatus*; Zcam: *Z. camerounensis*; Zdav: *Z. davidi*; Zgab: *Z. gabonicus*; Zafr: *Z. africanus*; Zind: *Z. indianus*.
