# Supplementary material for: Multiple invasions of Gypsy and Micropia retroelements in genus Zaprionus and melanogaster subgroup of the genus Drosophila
Source: BMC Evol Biol. 2009 Dec 2;9:279. doi: 10.1186/1471-2148-9-279 (PMC2797524; doi:10.1186/1471-2148-9-279)
Supplement: Additional file 10 — dN (below) and dS (above) values of pairwise comparisons between Micropia sequences of Zaprionus and melanogaster species. Distances calculated by the Nei-Gojobori method with Jukes-Cantor's correction as implemented by MEGA 4.1. [file 1471-2148-9-279-S10.DOC]

**Additional file 10. dN (below) and dS (above) values of pairwise comparisons between *Micropia* sequences of *Zaprionus* and *melanogaster* species.**

|  | 1 | 2 | 3 | 4 | 5 | 6 | 7 | 8 | 9 | 10 | 11 | 12 | 13 | 14 | 15 | 16 | 17 | 18 | 19 | 20 |
| --- | --- | --- | --- | --- | --- | --- | --- | --- | --- | --- | --- | --- | --- | --- | --- | --- | --- | --- | --- | --- |
| 1. DmelA1 | - | .000 | .000 | .000 | .000 | .000 | .016 | .000 | .000 | .000 | .000 | .000 | .065 | .099 | .209 | .000 | .032 | .155 | .154 | .187 |
| 2. DmelA3 | .005 | - | .000 | .000 | .000 | .000 | .016 | .000 | .000 | .000 | .000 | .000 | .065 | .099 | .209 | .000 | .032 | .155 | .154 | .187 |
| 3. DdmelA4 | .000 | .005 | - | .000 | .000 | .000 | .016 | .000 | .000 | .000 | .000 | .000 | .065 | .099 | .209 | .000 | .032 | .155 | .154 | .187 |
| 4. DsimA1 | .000 | .005 | .000 | - | .000 | .000 | .016 | .000 | .000 | .000 | .000 | .000 | .065 | .099 | .209 | .000 | .032 | .155 | .154 | .187 |
| 5. DsimA2 | .005 | .009 | .005 | .005 | - | .000 | .016 | .000 | .000 | .000 | .000 | .000 | .065 | .099 | .209 | .000 | .032 | .155 | .154 | .187 |
| 6. DsimA4 | .000 | .005 | .000 | .000 | .005 | - | .016 | .000 | .000 | .000 | .000 | .000 | .065 | .099 | .209 | .000 | .032 | .155 | .154 | .187 |
| 7. DsimA5 | .009 | .014 | .009 | .009 | .014 | .009 | - | .016 | .016 | .016 | .016 | .016 | .048 | .116 | .234 | .016 | .048 | .175 | .174 | .209 |
| 8. DsecA1 | .000 | .005 | .000 | .000 | .005 | .000 | .009 | - | .000 | .000 | .000 | .000 | .065 | .099 | .209 | .000 | .032 | .155 | .154 | .187 |
| 9. DsecA2 | .000 | .005 | .000 | .000 | .005 | .000 | .009 | .000 | - | .000 | .000 | .000 | .065 | .099 | .209 | .000 | .032 | .155 | .154 | .187 |
| 10. DsecA5 | .009 | .014 | .009 | .009 | .014 | .009 | .019 | .009 | .009 | - | .000 | .000 | .065 | .099 | .209 | .000 | .032 | .156 | .155 | .198 |
| 11. DsecA7 | .005 | .009 | .005 | .005 | .009 | .005 | .014 | .005 | .005 | .014 | - | .000 | .065 | .099 | .210 | .000 | .032 | .156 | .155 | .188 |
| 12. DsecA8 | .005 | .009 | .005 | .005 | .009 | .005 | .014 | .005 | .005 | .014 | .009 | - | .066 | .099 | .209 | .000 | .032 | .156 | .155 | .188 |
| 13. DsecA11 | .005 | .009 | .005 | .005 | .009 | .005 | .014 | .005 | .005 | .014 | .009 | .009 | - | .173 | .287 | .066 | .100 | .238 | .236 | .255 |
| 14. DsecA19 | .019 | .024 | .019 | .019 | .024 | .019 | .029 | .019 | .019 | .029 | .024 | .024 | .024 | - | .287 | .099 | .064 | .255 | .253 | .274 |
| 15. DsecA20 | .097 | .090 | .097 | .097 | .103 | .097 | .110 | .097 | .097 | .090 | .103 | .097 | .103 | .124 | - | .210 | .233 | .077 | .086 | .130 |
| 16. DsecB8 | .009 | .014 | .009 | .009 | .014 | .009 | .019 | .009 | .009 | .019 | .014 | .014 | .009 | .029 | .110 | - | .032 | .156 | .155 | .188 |
| 17. DsecB19 | .000 | .005 | .000 | .000 | .005 | .000 | .009 | .000 | .000 | .009 | .005 | .005 | .005 | .019 | .097 | .009 | - | .195 | .193 | .209 |
| 18. DyakA1 | .053 | .048 | .053 | .053 | .058 | .053 | .063 | .053 | .053 | .063 | .058 | .058 | .058 | .074 | .090 | .063 | .053 | - | .048 | .070 |
| 19. DyakA2 | .058 | .053 | .058 | .058 | .063 | .058 | .068 | .058 | .058 | .068 | .063 | .063 | .063 | .079 | .107 | .068 | .058 | .048 | - | .051 |
| 20. DyakB3 | .074 | .069 | .074 | .074 | .08 | .074 | .086 | .074 | .074 | .072 | .080 | .080 | .080 | .097 | .087 | .086 | .074 | .052 | .075 | - |
| 21. Ztub1 | .014 | .019 | .014 | .014 | .019 | .014 | .024 | .014 | .014 | .024 | .019 | .019 | .019 | .033 | .116 | .024 | .014 | .068 | .073 | .091 |
| 22. Ztub2 | .009 | .014 | .009 | .009 | .014 | .009 | .019 | .009 | .009 | .019 | .014 | .014 | .014 | .029 | .103 | .019 | .009 | .063 | .068 | .086 |
| 23. Ztub3 | .014 | .014 | .014 | .014 | .019 | .014 | .024 | .014 | .014 | .024 | .019 | .019 | .019 | .036 | .100 | .024 | .014 | .063 | .068 | .078 |
| 24. Zcam1 | .033 | .029 | .033 | .033 | .038 | .033 | .043 | .033 | .033 | .043 | .038 | .038 | .038 | .053 | .071 | .043 | .033 | .024 | .033 | .042 |
| 25. Zcam2 | .038 | .033 | .038 | .038 | .043 | .038 | .048 | .038 | .038 | .048 | .043 | .043 | .043 | .058 | .078 | .048 | .038 | .028 | .038 | .047 |
| 26. Zcam3 | .038 | .033 | .038 | .038 | .043 | .038 | .048 | .038 | .038 | .048 | .043 | .043 | .043 | .058 | .075 | .048 | .038 | .029 | .038 | .045 |
| 27. Zdav1 | .053 | .048 | .053 | .053 | .058 | .053 | .063 | .053 | .053 | .063 | .058 | .058 | .058 | .073 | .090 | .063 | .053 | .036 | .053 | .055 |
| 28. Zdav2 | .038 | .033 | .038 | .038 | .043 | .038 | .048 | .038 | .038 | .048 | .043 | .043 | .043 | .058 | .084 | .048 | .038 | .021 | .038 | .050 |
| 29. Zdav3 | .043 | .038 | .043 | .043 | .048 | .043 | .053 | .043 | .043 | .053 | .048 | .048 | .048 | .063 | .090 | .053 | .043 | .026 | .043 | .055 |
| 30. Zgab1 | .038 | .033 | .038 | .038 | .043 | .038 | .048 | .038 | .038 | .048 | .043 | .043 | .043 | .058 | .084 | .048 | .038 | .021 | .038 | .050 |
| 31. Zgab2 | .038 | .033 | .038 | .038 | .043 | .038 | .048 | .038 | .038 | .048 | .043 | .043 | .043 | .058 | .084 | .048 | .038 | .021 | .038 | .050 |
| 32. Zgab3 | .041 | .036 | .041 | .041 | .046 | .041 | .051 | .041 | .041 | .051 | .046 | .046 | .046 | .062 | .087 | .05 | .041 | .026 | .043 | .055 |
| 33. Zafr1 | .059 | .054 | .059 | .059 | .064 | .059 | .07 | .059 | .059 | .07 | .054 | .064 | .064 | .080 | .100 | .070 | .059 | .041 | .059 | .072 |
| 34. Zafr2 | .054 | .049 | .054 | .054 | .059 | .054 | .065 | .054 | .054 | .064 | .049 | .059 | .059 | .075 | .100 | .064 | .054 | .037 | .054 | .067 |
| 35. Zafr3 | .038 | .033 | .038 | .038 | .043 | .038 | .048 | .038 | .038 | .048 | .043 | .043 | .043 | .058 | .084 | .048 | .038 | .021 | .038 | .050 |
| 36. Zind1 | .043 | .038 | .043 | .043 | .048 | .043 | .053 | .043 | .043 | .053 | .048 | .048 | .048 | .063 | .090 | .053 | .043 | .026 | .043 | .055 |
| 37. Zind2 | .038 | .033 | .038 | .038 | .043 | .038 | .048 | .038 | .038 | .048 | .043 | .043 | .043 | .058 | .084 | .048 | .038 | .021 | .038 | .050 |
| 38. Zind3 | .038 | .033 | .038 | .038 | .043 | .038 | .048 | .038 | .038 | .048 | .043 | .043 | .043 | .058 | .084 | .048 | .038 | .021 | .038 | .050 |

**Additional file 10, continuation.**

|  | 21 | 22 | 23 | 24 | 25 | 26 | 27 | 28 | 29 | 30 | 31 | 32 | 33 | 34 | 35 | 36 | 37 | 38 |
| --- | --- | --- | --- | --- | --- | --- | --- | --- | --- | --- | --- | --- | --- | --- | --- | --- | --- | --- |
| 1. DmelA1 | .016 | .032 | .016 | .154 | .155 | .154 | .137 | .137 | .137 | .137 | .156 | .146 | .141 | .140 | .137 | .118 | .137 | .137 |
| 2. DmelA3 | .016 | .032 | .016 | .154 | .155 | .154 | .137 | .137 | .137 | .137 | .156 | .146 | .141 | .140 | .137 | .118 | .137 | .137 |
| 3. DdmelA4 | .016 | .032 | .016 | .154 | .155 | .154 | .137 | .137 | .137 | .137 | .156 | .146 | .141 | .140 | .137 | .118 | .137 | .137 |
| 4. DsimA1 | .016 | .032 | .016 | .154 | .155 | .154 | .137 | .137 | .137 | .137 | .156 | .146 | .141 | .140 | .137 | .118 | .137 | .137 |
| 5. DsimA2 | .016 | .032 | .016 | .154 | .155 | .154 | .137 | .137 | .137 | .137 | .156 | .146 | .141 | .140 | .137 | .118 | .137 | .137 |
| 6. DsimA4 | .016 | .032 | .016 | .154 | .155 | .154 | .137 | .137 | .137 | .137 | .156 | .146 | .141 | .140 | .137 | .118 | .137 | .137 |
| 7. DsimA5 | .032 | .048 | .032 | .174 | .175 | .173 | .156 | .156 | .156 | .156 | .175 | .165 | .160 | .159 | .156 | .137 | .156 | .156 |
| 8. DsecA1 | .016 | .032 | .016 | .154 | .155 | .154 | .137 | .137 | .137 | .137 | .156 | .146 | .141 | .140 | .137 | .118 | .137 | .137 |
| 9. DsecA2 | .016 | .032 | .016 | .154 | .155 | .154 | .137 | .137 | .137 | .137 | .156 | .146 | .141 | .140 | .137 | .118 | .137 | .137 |
| 10. DsecA5 | .016 | .032 | .016 | .155 | .156 | .154 | .137 | .137 | .137 | .137 | .156 | .146 | .141 | .140 | .137 | .118 | .137 | .137 |
| 11. DsecA7 | .016 | .032 | .016 | .155 | .156 | .154 | .137 | .137 | .137 | .137 | .156 | .146 | .141 | .140 | .137 | .118 | .137 | .137 |
| 12. DsecA8 | .016 | .032 | .016 | .155 | .156 | .154 | .138 | .137 | .138 | .137 | .156 | .146 | .141 | .140 | .137 | .119 | .137 | .137 |
| 13. DsecA11 | .084 | .100 | .083 | .236 | .238 | .235 | .217 | .217 | .217 | .217 | .238 | .227 | .224 | .222 | .217 | .196 | .217 | .217 |
| 14. DsecA19 | .118 | .134 | .107 | .253 | .255 | .252 | .234 | .234 | .234 | .234 | .255 | .236 | .241 | .239 | .234 | .213 | .234 | .234 |
| 15. DsecA20 | .188 | .162 | .197 | .057 | .057 | .066 | .057 | .057 | .057 | .057 | .077 | .067 | .067 | .067 | .057 | .077 | .057 | .057 |
| 16. DsecB8 | .016 | .032 | .016 | .155 | .156 | .154 | .138 | .137 | .138 | .137 | .156 | .146 | .141 | .140 | .137 | .119 | .137 | .137 |
| 17. DsecB19 | .049 | .065 | .048 | .193 | .195 | .192 | .175 | .175 | .175 | .175 | .195 | .184 | .180 | .179 | .175 | .156 | .175 | .175 |
| 18. DyakA1 | .138 | .117 | .136 | .048 | .048 | .048 | .040 | .040 | .040 | .040 | .057 | .040 | .041 | .041 | .040 | .057 | .040 | .040 |
| 19. DyakA2 | .137 | .117 | .135 | .032 | .032 | .031 | .016 | .016 | .016 | .016 | .032 | .016 | .016 | .016 | .016 | .032 | .016 | .016 |
| 20. DyakB3 | .169 | .145 | .173 | .034 | .034 | .039 | .061 | .043 | .043 | .043 | .061 | .043 | .043 | .043 | .043 | .061 | .043 | .043 |
| 21. Ztub1 | - | .016 | .000 | .138 | .138 | .137 | .120 | .120 | .120 | .12 | .139 | .129 | .123 | .123 | .120 | .139 | .120 | .120 |
| 22. Ztub2 | .024 | - | .016 | .117 | .117 | .116 | .100 | .100 | .100 | .100 | .118 | .108 | .102 | .102 | .100 | .118 | .100 | .100 |
| 23. Ztub3 | .028 | .024 | - | .136 | .136 | .135 | .128 | .118 | .118 | .118 | .137 | .127 | .121 | .121 | .118 | .137 | .118 | .118 |
| 24. Zcam1 | .048 | .043 | .043 | - | .000 | .000 | .016 | .016 | .016 | .016 | .032 | .016 | .000 | .000 | .016 | .032 | .016 | .016 |
| 25. Zcam2 | .053 | .048 | .048 | .005 | - | .000 | .016 | .016 | .016 | .016 | .032 | .016 | .000 | .000 | .016 | .032 | .016 | .016 |
| 26. Zcam3 | .053 | .048 | .038 | .005 | .009 | - | .024 | .016 | .016 | .016 | .032 | .016 | .000 | .000 | .016 | .032 | .016 | .016 |
| 27. Zdav1 | .068 | .063 | .06 | .028 | .033 | .031 | - | .000 | .000 | .000 | .016 | .000 | .000 | .000 | .000 | .016 | .000 | .000 |
| 28. Zdav2 | .053 | .048 | .048 | .014 | .019 | .019 | .014 | - | .000 | .000 | .016 | .000 | .000 | .000 | .000 | .016 | .000 | .000 |
| 29. Zdav3 | .058 | .053 | .053 | .019 | .024 | .024 | .009 | .005 | - | .000 | .016 | .000 | .000 | .000 | .000 | .016 | .000 | .000 |
| 30. Zgab1 | .053 | .048 | .048 | .014 | .019 | .019 | .014 | .000 | .005 | - | .016 | .000 | .000 | .000 | .000 | .016 | .000 | .000 |
| 31. Zgab2 | .053 | .048 | .048 | .014 | .019 | .019 | .014 | .000 | .005 | .000 | - | .016 | .016 | .016 | .016 | .032 | .016 | .016 |
| 32. Zgab3 | .055 | .051 | .051 | .019 | .024 | .024 | .019 | .005 | .009 | .005 | .005 | - | .000 | .000 | .000 | .016 | .000 | .000 |
| 33. Zafr1 | .075 | .07 | .064 | .034 | .039 | .039 | .034 | .019 | .024 | .019 | .019 | .024 | - | .000 | .000 | .016 | .000 | .000 |
| 34. Zafr2 | .069 | .065 | .059 | .029 | .034 | .034 | .029 | .014 | .019 | .014 | .014 | .019 | .005 | - | .000 | .016 | .000 | .000 |
| 35. Zafr3 | .053 | .048 | .048 | .014 | .019 | .019 | .014 | .000 | .005 | .000 | .000 | .005 | .019 | .014 | - | .016 | .000 | .000 |
| 36. Zind1 | .058 | .053 | .053 | .019 | .024 | .024 | .019 | .005 | .009 | .005 | .005 | .009 | .024 | .019 | .005 | - | .016 | .016 |
| 37. Zind2 | .053 | .048 | .048 | .014 | .019 | .019 | .014 | .000 | .005 | .000 | .000 | .005 | .019 | .014 | .000 | .005 | - | .000 |
| 38. Zind3 | .053 | .048 | .048 | .014 | .019 | .019 | .014 | .000 | .005 | .000 | .000 | .005 | .019 | .014 | .000 | .005 | .000 | - |

Symbols for species names: Dmel: *D. melanogaster*; Dsim: *D. simulans*; Dsec: *D. sechellia*; Dyak: *D. yakuba*; Ztub: *Z. tuberculatus*; Zcam: *Z. camerounensis*; Zdav: *Z. davidi*; Zgab: *Z. gabonicus*; Zafr: *Z. africanus*; Zind: *Z. indianus*.
