# Supplementary material for: Multiple invasions of Gypsy and Micropia retroelements in genus Zaprionus and melanogaster subgroup of the genus Drosophila
Source: BMC Evol Biol. 2009 Dec 2;9:279. doi: 10.1186/1471-2148-9-279 (PMC2797524; doi:10.1186/1471-2148-9-279)
Supplement: Additional file 11 — Z values (above) and significance P-values (below) for the Codon-Based Z-test of selection between Micropia sequences of Zaprionus and melanogaster species. Test performed using alternative hypothesis of non-neutrality (dN ≠ dS) and Nei-Gojobori distance (Jukes-Cantor's correction). Gray cells correspond to significant (p < 0.05) pairwise comparisons. [file 1471-2148-9-279-S11.DOC]

**Additional file 11. Z values (above) and significance P-values (below) for the Codon-Based Z-test of selection between *Micropia* sequences of *Zaprionus* and *melanogaster* species.**

|  | 1 | 2 | 3 | 4 | 5 | 6 | 7 | 8 | 9 | 10 | 11 | 12 | 13 | 14 | 15 | 16 | 17 | 18 | 19 | 20 |
| --- | --- | --- | --- | --- | --- | --- | --- | --- | --- | --- | --- | --- | --- | --- | --- | --- | --- | --- | --- | --- |
| 1. DmelA1 | - | .999 | .000 | .000 | .999 | .000 | -.372 | .000 | .000 | 1.412 | .999 | .999 | -1.818 | -1.893 | -1.533 | 1.412 | -1.407 | -1.835 | -1.727 | -1.743 |
| 2. DmelA3 | .320 | - | .999 | .999 | 1.412 | .999 | -.092 | .999 | .999 | 1.728 | 1.412 | 1.412 | -1.660 | -1.767 | -1.627 | 1.728 | -1.175 | -1.933 | -1.825 | -1.837 |
| 3. DdmelA4 | 1.000 | .320 | - | .000 | .999 | .000 | -.372 | .000 | .000 | 1.412 | .999 | .999 | -1.818 | -1.893 | -1.533 | 1.412 | -1.407 | -1.835 | -1.727 | -1.743 |
| 4. DsimA1 | 1.000 | .320 | 1.000 | - | .999 | .000 | -.372 | .000 | .000 | 1.412 | .999 | .999 | -1.818 | -1.893 | -1.533 | 1.412 | -1.407 | -1.835 | -1.727 | -1.743 |
| 5. DsimA2 | .320 | .161 | .320 | .320 | - | .999 | -.092 | .999 | .999 | 1.728 | 1.412 | 1.412 | -1.660 | -1.767 | -1.438 | 1.728 | -1.175 | -1.738 | -1.629 | -1.650 |
| 6. DsimA4 | 1.000 | .320 | 1.000 | 1.000 | .320 | - | -.372 | .000 | .000 | 1.412 | .999 | .999 | -1.818 | -1.893 | -1.533 | 1.412 | -1.407 | -1.835 | -1.727 | -1.743 |
| 7. DsimA5 | .711 | .927 | .711 | .711 | .927 | .711 | - | -.372 | -.372 | .167 | -.094 | -.096 | -1.171 | -1.891 | -1.580 | .165 | -1.347 | -1.867 | -1.764 | -1.781 |
| 8. DsecA1 | 1.000 | .320 | 1.000 | 1.000 | .320 | 1.000 | .711 | - | .000 | 1.412 | .999 | .999 | -1.818 | -1.893 | -1.533 | 1.412 | -1.407 | -1.835 | -1.727 | -1.743 |
| 9. DsecA2 | 1.000 | .320 | 1.000 | 1.000 | .320 | 1.000 | .711 | 1.000 | - | 1.412 | .999 | .999 | -1.818 | -1.893 | -1.533 | 1.412 | -1.407 | -1.835 | -1.727 | -1.743 |
| 10. DsecA5 | .161 | .087 | .161 | .161 | .087 | .161 | .868 | .161 | .161 | - | 1.728 | 1.728 | -1.507 | -1.645 | -1.627 | 1.994 | -.953 | -1.645 | -1.536 | -1.902 |
| 11. DsecA7 | .320 | .161 | .320 | .320 | .161 | .320 | .925 | .320 | .320 | .087 | - | 1.412 | -1.661 | -1.770 | -1.445 | 1.728 | -1.175 | -1.742 | -1.634 | -1.655 |
| 12. DsecA8 | .320 | .161 | .320 | .320 | .161 | .320 | .924 | .320 | .320 | .087 | .161 | - | -1.662 | -1.771 | -1.533 | 1.728 | -1.176 | -1.744 | -1.636 | -1.658 |
| 13. DsecA11 | .072 | .099 | .072 | .072 | .099 | .072 | .244 | .072 | .072 | .134 | .099 | .099 | - | -2.594 | -2.081 | -1.662 | -2.279 | -2.522 | -2.436 | -2.262 |
| 14. DsecA19 | .061 | .080 | .061 | .061 | .080 | .061 | .061 | .061 | .061 | .103 | .079 | .079 | .011 | - | -1.799 | -1.646 | -1.338 | -2.438 | -2.352 | -2.176 |
| 15. DsecA20 | .128 | .106 | .128 | .128 | .153 | .128 | .117 | .128 | .128 | .106 | .151 | .128 | .040 | .074 | - | -1.354 | -1.756 | .291 | .414 | -.754 |
| 16. DsecB8 | .161 | .087 | .161 | .161 | .087 | .161 | .869 | .161 | .161 | .048 | .087 | .087 | .099 | .102 | .178 | - | -.953 | -1.648 | -1.539 | -1.565 |
| 17. DsecB19 | .162 | .242 | .162 | .162 | .242 | .162 | .181 | .162 | .162 | .343 | .242 | .242 | .024 | .183 | .082 | .342 | - | -2.244 | -2.148 | -1.955 |
| 18. DyakA1 | .069 | .056 | .069 | .069 | .085 | .069 | .064 | .069 | .069 | .102 | .084 | .084 | .013 | .016 | .771 | .102 | .027 | - | .001 | -.441 |
| 19. DyakA2 | .087 | .070 | .087 | .087 | .106 | .087 | .080 | .087 | .087 | .127 | .105 | .104 | .016 | .020 | .680 | .126 | .034 | .999 | - | .649 |
| 20. DyakB3 | .084 | .069 | .084 | .084 | .102 | .084 | .077 | .084 | .084 | .060 | .100 | .100 | .026 | .032 | .452 | .120 | .053 | .660 | .517 | - |
| 21. Ztub1 | .917 | .877 | .917 | .917 | .877 | .917 | .732 | .917 | .917 | .693 | .879 | .881 | .100 | .076 | .306 | .694 | .242 | .190 | .231 | .215 |
| 22. Ztub2 | .345 | .466 | .345 | .345 | .466 | .345 | .326 | .345 | .345 | .601 | .464 | .464 | .045 | .037 | .362 | .600 | .100 | .268 | .324 | .301 |
| 23. Ztub3 | .928 | .928 | .928 | .928 | .864 | .928 | .745 | .928 | .928 | .679 | .866 | .868 | .102 | .114 | .175 | .680 | .246 | .166 | .204 | .129 |
| 24. Zcam1 | .028 | .022 | .028 | .028 | .036 | .028 | .028 | .028 | .028 | .045 | .036 | .036 | .006 | .007 | .705 | .045 | .011 | .416 | .942 | .780 |
| 25. Zcam2 | .035 | .028 | .035 | .035 | .045 | .035 | .034 | .035 | .035 | .055 | .044 | .044 | .007 | .009 | .605 | .055 | .014 | .514 | .805 | .653 |
| 26. Zcam3 | .036 | .029 | .036 | .036 | .046 | .036 | .035 | .036 | .036 | .057 | .046 | .045 | .007 | .009 | .835 | .057 | .014 | .523 | .790 | .843 |
| 27. Zdav1 | .111 | .090 | .111 | .111 | .135 | .111 | .102 | .111 | .111 | .162 | .134 | .133 | .020 | .025 | .423 | .161 | .042 | .873 | .101 | .877 |
| 28. Zdav2 | .058 | .046 | .058 | .058 | .073 | .058 | .055 | .058 | .058 | .090 | .072 | .072 | .011 | .014 | .508 | .089 | .022 | .492 | .281 | .829 |
| 29. Zdav3 | .072 | .057 | .072 | .072 | .090 | .072 | .068 | .072 | .072 | .110 | .089 | .089 | .013 | .017 | .423 | .109 | .027 | .611 | .204 | .707 |
| 30. Zgab1 | .058 | .046 | .058 | .058 | .073 | .058 | .055 | .058 | .058 | .090 | .072 | .072 | .011 | .014 | .508 | .089 | .022 | .492 | .281 | .829 |
| 31. Zgab2 | .035 | .028 | .035 | .035 | .044 | .035 | .034 | .035 | .035 | .055 | .044 | .044 | .007 | .009 | .881 | .055 | .014 | .272 | .806 | .765 |
| 32. Zgab3 | .051 | .040 | .051 | .051 | .064 | .051 | .049 | .051 | .051 | .079 | .063 | .063 | .010 | .015 | .637 | .078 | .019 | .615 | .201 | .699 |
| 33. Zafr1 | .134 | .110 | .134 | .134 | .162 | .134 | .122 | .134 | .134 | .193 | .109 | .160 | .024 | .030 | .457 | .192 | .052 | .995 | .071 | .393 |
| 34. Zafr2 | .112 | .091 | .112 | .112 | .136 | .112 | .103 | .112 | .112 | .164 | .090 | .135 | .020 | .025 | .457 | .163 | .043 | .878 | .100 | .475 |
| 35. Zafr3 | .058 | .046 | .058 | .058 | .073 | .058 | .055 | .058 | .058 | .090 | .072 | .072 | .011 | .014 | .508 | .089 | .022 | .492 | .281 | .829 |
| 36. Zind1 | .120 | .096 | .120 | .120 | .148 | .120 | .111 | .120 | .120 | .180 | .147 | .147 | .021 | .026 | .774 | .179 | .044 | .347 | .672 | .882 |
| 37. Zind2 | .058 | .046 | .058 | .058 | .073 | .058 | .055 | .058 | .058 | .090 | .072 | .072 | .011 | .014 | .508 | .089 | .022 | .492 | .281 | .829 |
| 38. Zind3 | .058 | .046 | .058 | .058 | .073 | .058 | .055 | .058 | .058 | .090 | .072 | .072 | .011 | .014 | .508 | .089 | .022 | .492 | .281 | .829 |

**Additional file 11, continuation**.

|  | 21 | 22 | 23 | 24 | 25 | 26 | 27 | 28 | 29 | 30 | 31 | 32 | 33 | 34 | 35 | 36 | 37 | 38 |
| --- | --- | --- | --- | --- | --- | --- | --- | --- | --- | --- | --- | --- | --- | --- | --- | --- | --- | --- |
| 1. DmelA1 | -.104 | -.949 | -.090 | -2.221 | -2.128 | -2.116 | -1.608 | -1.915 | -1.814 | -1.915 | -2.129 | -1.971 | -1.509 | -1.603 | -1.915 | -1.567 | -1.915 | -1.915 |
| 2. DmelA3 | .155 | -.732 | -.090 | -2.319 | -2.225 | -2.216 | -1.711 | -2.019 | -1.918 | -2.019 | -2.227 | -2.072 | -1.611 | -1.706 | -2.019 | -1.678 | -2.019 | -2.019 |
| 3. DdmelA4 | -.104 | -.949 | -.090 | -2.221 | -2.128 | -2.116 | -1.608 | -1.915 | -1.814 | -1.915 | -2.129 | -1.971 | -1.509 | -1.603 | -1.915 | -1.567 | -1.915 | -1.915 |
| 4. DsimA1 | -.104 | -.949 | -.090 | -2.221 | -2.128 | -2.116 | -1.608 | -1.915 | -1.814 | -1.915 | -2.129 | -1.971 | -1.509 | -1.603 | -1.915 | -1.567 | -1.915 | -1.915 |
| 5. DsimA2 | .155 | -.732 | .172 | -2.122 | -2.030 | -2.017 | -1.505 | -1.811 | -1.711 | -1.811 | -2.032 | -1.870 | -1.406 | -1.499 | -1.811 | -1.455 | -1.811 | -1.811 |
| 6. DsimA4 | -.104 | -.949 | -.090 | -2.221 | -2.128 | -2.116 | -1.608 | -1.915 | -1.814 | -1.915 | -2.129 | -1.971 | -1.509 | -1.603 | -1.915 | -1.567 | -1.915 | -1.915 |
| 7. DsimA5 | -.343 | -.987 | -.326 | -2.227 | -2.142 | -2.128 | -1.650 | -1.936 | -1.843 | -1.936 | -2.143 | -1.991 | -1.558 | -1.645 | -1.936 | -1.606 | -1.936 | -1.936 |
| 8. DsecA1 | -.104 | -.949 | -.090 | -2.221 | -2.128 | -2.116 | -1.608 | -1.915 | -1.814 | -1.915 | -2.129 | -1.971 | -1.509 | -1.603 | -1.915 | -1.567 | -1.915 | -1.915 |
| 9. DsecA2 | -.104 | -.949 | -.090 | -2.221 | -2.128 | -2.116 | -1.608 | -1.915 | -1.814 | -1.915 | -2.129 | -1.971 | -1.509 | -1.603 | -1.915 | -1.567 | -1.915 | -1.915 |
| 10. DsecA5 | .396 | -.525 | .415 | -2.027 | -1.936 | -1.922 | -1.407 | -1.711 | -1.612 | -1.711 | -1.938 | -1.773 | -1.309 | -1.401 | -1.711 | -1.349 | -1.711 | -1.711 |
| 11. DsecA7 | .152 | -.734 | .169 | -2.125 | -2.033 | -2.020 | -1.509 | -1.814 | -1.715 | -1.814 | -2.035 | -1.874 | -1.616 | -1.710 | -1.814 | -1.459 | -1.814 | -1.814 |
| 12. DsecA8 | .150 | -.735 | .167 | -2.126 | -2.035 | -2.022 | -1.511 | -1.816 | -1.717 | -1.816 | -2.037 | -1.875 | -1.414 | -1.506 | -1.816 | -1.461 | -1.816 | -1.816 |
| 13. DsecA11 | -1.658 | -2.026 | -1.648 | -2.825 | -2.753 | -2.743 | -2.356 | -2.595 | -2.517 | -2.595 | -2.754 | -2.634 | -2.278 | -2.351 | -2.595 | -2.339 | -2.595 | -2.595 |
| 14. DsecA19 | -1.789 | -2.108 | -1.590 | -2.733 | -2.664 | -2.651 | -2.272 | -2.504 | -2.429 | -2.504 | -2.665 | -2.463 | -2.196 | -2.266 | -2.504 | -2.248 | -2.504 | -2.504 |
| 15. DsecA20 | -1.028 | -.915 | -1.365 | .830 | .518 | .208 | .804 | .665 | .804 | .665 | .150 | .473 | .746 | .746 | .665 | .287 | .665 | .665 |
| 16. DsecB8 | .394 | -.526 | .413 | -2.029 | -1.938 | -1.924 | -1.409 | -1.713 | -1.614 | -1.713 | -1.940 | -1.775 | -1.312 | -1.404 | -1.713 | -1.351 | -1.713 | -1.713 |
| 17. DsecB19 | -1.176 | -1.656 | -1.166 | -2.586 | -2.504 | -2.494 | -2.054 | -2.325 | -2.236 | -2.325 | -2.505 | -2.370 | -1.966 | -2.050 | -2.325 | -2.032 | -2.325 | -2.325 |
| 18. DyakA1 | -1.318 | -1.114 | -1.395 | -.817 | -.655 | -.640 | -.160 | -.690 | -.511 | -.690 | -1.104 | -.504 | .006 | -.154 | -.690 | -.944 | -.690 | -.690 |
| 19. DyakA2 | -1.203 | -.990 | -1.278 | .072 | .248 | .267 | 1.651 | 1.082 | 1.277 | 1.082 | .246 | 1.287 | 1.824 | 1.659 | 1.082 | .425 | 1.082 | 1.082 |
| 20. DyakB3 | -1.248 | -1.038 | -1.527 | .280 | .451 | .199 | -.155 | .217 | .376 | .217 | -.299 | .388 | .858 | .717 | .217 | -.149 | .217 | .217 |
| 21. Ztub1 | - | .405 | 2.438 | -1.717 | -1.622 | -1.606 | -1.047 | -1.367 | -1.263 | -1.367 | -1.624 | -1.442 | -.946 | -1.042 | -1.367 | -1.523 | -1.367 | -1.367 |
| 22. Ztub2 | .686 | - | .424 | -1.552 | -1.447 | -1.431 | -.815 | -1.166 | -1.051 | -1.166 | -1.449 | -1.250 | -.702 | -.808 | -1.166 | -1.338 | -1.166 | -1.166 |
| 23. Ztub3 | .016 | .672 | - | -1.802 | -1.705 | -1.901 | -1.322 | -1.455 | -1.349 | -1.455 | -1.707 | -1.527 | -1.134 | -1.233 | -1.455 | -1.603 | -1.455 | -1.455 |
| 24. Zcam1 | .089 | .123 | .074 | - | .999 | .999 | .645 | -.092 | .167 | -.092 | -.736 | .174 | 2.630 | 2.437 | -.092 | -.528 | -.092 | -.092 |
| 25. Zcam2 | .107 | .150 | .091 | .320 | - | 1.412 | .857 | .164 | .405 | .164 | -.534 | .413 | 2.809 | 2.630 | .164 | -.333 | .164 | .164 |
| 26. Zcam3 | .111 | .155 | .060 | .320 | .161 | - | .317 | .177 | .421 | .177 | -.522 | .428 | 2.809 | 2.630 | .177 | -.319 | .177 | .177 |
| 27. Zdav1 | .297 | .417 | .189 | .520 | .393 | .752 | - | 1.728 | 1.412 | 1.728 | -.101 | 1.994 | 2.630 | 2.437 | 1.728 | .159 | 1.728 | 1.728 |
| 28. Zdav2 | .174 | .246 | .148 | .927 | .870 | .860 | .087 | - | .999 | .000 | -.997 | .999 | 1.993 | 1.728 | .000 | -.675 | .000 | .000 |
| 29. Zdav3 | .209 | .295 | .180 | .868 | .686 | .675 | .161 | .320 | - | .999 | -.676 | 1.412 | 2.227 | 1.993 | .999 | -.379 | .999 | .999 |
| 30. Zgab1 | .174 | .246 | .148 | .927 | .870 | .860 | .087 | 1.000 | .320 | - | -.997 | .999 | 1.993 | 1.728 | .000 | -.675 | .000 | .000 |
| 31. Zgab2 | .107 | .150 | .090 | .463 | .595 | .603 | .919 | .321 | .500 | .321 | - | -.674 | .156 | -.098 | -.997 | -1.177 | -.997 | -.997 |
| 32. Zgab3 | .152 | .214 | .129 | .862 | .680 | .669 | .048 | .320 | .161 | .320 | .502 | - | 2.227 | 1.993 | .999 | -.375 | .999 | .999 |
| 33. Zafr1 | .346 | .484 | .259 | .010 | .006 | .006 | .010 | .048 | .028 | .048 | .876 | .028 | - | .999 | 1.993 | .400 | 1.993 | 1.993 |
| 34. Zafr2 | .300 | .421 | .220 | .016 | .010 | .010 | .016 | .087 | .048 | .087 | .922 | .048 | .320 | - | 1.728 | .163 | 1.728 | 1.728 |
| 35. Zafr3 | .174 | .246 | .148 | .927 | .870 | .860 | .087 | 1.000 | .320 | 1.000 | .321 | .320 | .048 | .087 | - | -.675 | .000 | .000 |
| 36. Zind1 | .130 | .183 | .111 | .599 | .740 | .750 | .874 | .501 | .706 | .501 | .241 | .709 | .690 | .871 | .501 | - | -.675 | -.675 |
| 37. Zind2 | .174 | .246 | .148 | .927 | .870 | .860 | .087 | 1.000 | .320 | 1.000 | .321 | .320 | .048 | .087 | 1.000 | .501 | - | .000 |
| 38. Zind3 | .174 | .246 | .148 | .927 | .870 | .860 | .087 | 1.000 | .320 | 1.000 | .321 | .320 | .048 | .087 | 1.000 | .501 | 1.000 | - |

Symbols for species names: Dmel: *D. melanogaster*; Dsim: *D. simulans*; Dsec: *D. sechellia*; Dyak: *D. yakuba*; Ztub: *Z. tuberculatus*; Zcam: *Z. camerounensis*; Zdav: *Z. davidi*; Zgab: *Z. gabonicus*; Zafr: *Z. africanus*; Zind: *Z. indianus*.
